# Identification of the pre‐Bötzinger complex inspiratory center in calibrated “sandwich” slices from newborn mice with fluorescent Dbx1 interneurons

**DOI:** 10.14814/phy2.12111

**Published:** 2014-08-19

**Authors:** Araya Ruangkittisakul, Andrew Kottick, Maria C. D. Picardo, Klaus Ballanyi, Christopher A. Del Negro

**Affiliations:** 1 Department of Physiology, Faculty of Medicine & Dentistry, University of Alberta, Edmonton, Alberta, Canada; 2 Department of Applied Science, The College of William & Mary, Williamsburg, Virginia

**Keywords:** Breathing, central pattern generator, respiration

## Abstract

Inspiratory active pre‐Bötzinger complex (preBötC) networks produce the neural rhythm that initiates and controls breathing movements. We previously identified the preBötC in the newborn rat brainstem and established anatomically defined transverse slices in which the preBötC remains active when exposed at one surface. This follow‐up study uses a neonatal mouse model in which the preBötC as well as a genetically defined class of respiratory interneurons can be identified and selectively targeted for physiological recordings. The population of glutamatergic interneurons whose precursors express the transcription factor Dbx1 putatively comprises the core respiratory rhythmogenic circuit. Here, we used intersectional mouse genetics to identify the brainstem distribution of *Dbx1*‐derived neurons in the context of observable respiratory marker structures. This reference brainstem atlas enabled online histology for generating calibrated sandwich slices to identify the preBötC location, which was heretofore unspecified for perinatal mice. Sensitivity to opioids ensured that slice rhythms originated from preBötC neurons and not parafacial respiratory group/retrotrapezoid nucleus (pFRG/RTN) cells because opioids depress preBötC, but not pFRG/RTN rhythms. We found that the preBötC is centered ~0.4 mm caudal to the facial motor nucleus in this Cre/lox reporter mouse during postnatal days 0–4. Our findings provide the essential basis for future optically guided electrophysiological and fluorescence imaging‐based studies, as well as the application of other Cre‐dependent tools to record or manipulate respiratory rhythmogenic neurons. These resources will ultimately help elucidate the mechanisms that promote respiratory‐related oscillations of preBötC *Dbx1*‐derived neurons and thus breathing.

## Abstract (precis)

Breathing movements emanate from Dbx1‐derived interneurons of the brainstem pre‐Bötzinger complex (preBötC). We generated a histology atlas of the medulla in newborn Dbx1 Cre/lox reporter mice and performed physiological tests to pinpoint the preBötC location and map the Dbx1 neuron distribution, which will facilitate neurobiological studies of respiratory rhythm generation.

## Introduction

Inspiratory breathing movements in mammals are initiated by interneurons of the pre‐Bötzinger complex (preBötC) within the bilaterally organized ventral respiratory column (VRC) in the lower brainstem (Feldman et al. 2013). A central goal in respiratory neurobiology – and the major motivation for this study – is to accurately identify the preBötC within the heterogeneous VRC and study its principal class of rhythmogenic interneurons.

One evolving strategy to study the preBötC involves the use of in vitro preparations. The preBötC was identified by serial transection of the VRC in respiratory active newborn rat brainstem‐spinal cord preparations (Smith et al. 1991). Further transection experiments established that transverse brainstem slices capture essential circuitry to spontaneously generate inspiratory‐related neural rhythms and motor output. These “breathing slices” enable recording and imaging experiments from the respiratory neurons while maintaining behaviorally relevant function in vitro.

By systematically varying the rostral and caudal borders of such breathing slices, we found that the newborn rat preBötC is centered 0.5 mm caudal to the facial (VII) motor nucleus (Ruangkittisakul et al. 2006, 2008). These anatomically calibrated slices enable pharmacological and structure–function analyses of the isolated preBötC, but cannot per se identify its constituent rhythmogenic inspiratory neurons. For that purpose it is necessary to differentiate classes of preBötC neurons, target them for optically guided recordings (Ballanyi and Ruangkittisakul 2009), and then test their role(s) in the context of respiratory function.

Inspiratory active inhibitory GABAergic and glycinergic interneuron classes have been fluorescence‐tagged and visualized in preBötC slices using bacterial artificial chromosome transgenic and knock‐in mice (Kuwana et al. 2006; Winter et al. 2009). However, these neurons are not pivotal for rhythm generation because the preBötC remains active, both in vivo and in vitro, after blockade of GABA and glycine receptors (Feldman and Smith 1989; Shao and Feldman 1997; Brockhaus and Ballanyi 1998; Ren and Greer 2006; Janczewski et al. 2013). Instead, excitatory glutamatergic neurons are obligatory for rhythmogenesis as indicated by inhibitory actions of glutamate receptor antagonists on preBötC rhythm in vitro (Greer et al. 1991; Funk et al. 1993; Ge and Feldman 1998) and the absence of breathing ex utero and inspiratory rhythm in vitro in fetal mice lacking the vesicular glutamate transporter VGLUT2 (Wallén‐Mackenzie et al. 2006).

Glutamatergic interneurons in the preBötC are derived from precursors that express the homeodomain transcription factor protein Dbx1 (developing brain homeobox 1; Bouvier et al. 2010; Gray et al. 2010; Gray 2013). *Dbx1*‐derived neurons (henceforth Dbx1 neurons) can be visualized using *lacZ* knock‐in reporter mice (Pierani et al. 2001) but also intersectional mouse genetics, particularly Cre/lox recombination (Bouvier et al. 2010; Gray et al. 2010; Talpalar et al. 2013). *Dbx1*‐dependent Cre‐driver mice (Hirata et al. 2009) coupled with a widely available *floxed Rosa26*^*tdTomato*^ reporter (Madisen et al. 2010), which we refer to as Dbx1 reporter mice, allowed us to selectively record and characterize inspiratory Dbx1 neurons (Picardo et al. 2013). We proposed that the neurons were located within the preBötC, assuming that its location matched that in newborn rats, as described above. However, the preBötC has not yet been definitively identified in mice and thus we could not analyze whether these presumably rhythmogenic Dbx1 neurons form a specific cluster that distinguishes the inspiratory oscillator core from other areas within the VRC and neighboring brainstem regions.

Accordingly, we aimed here to determine the preBötC location in newborn mice using the same Cre/lox reporter that drives expression of tdTomato in Dbx1 neurons. Precisely knowing the preBötC location facilitates calibrated slices that expose the preBötC partly to one surface, for example, for optically guided electrophysiological recording and fluorescent (calcium) imaging analyses. To that end, we generated a brainstem atlas for Dbx1 reporter mice, which complements our previous work on neonatal Wistar and Sprague–Dawley rats (Ruangkittisakul et al. 2006, 2008) as well as C57BL/6 mice (Ruangkittisakul et al. 2011). Then, using the coordinates from the mouse atlas, we identify the preBötC physiologically based on two “sandwich” slices from a single brainstem, which each show rhythmic activity in the ventrolateral area. This approach in newborn rats shows that two inspiratory active slices (monitored via field potential recordings) can be generated from one brainstem provided the transection level is within approximately 100 *μ*m of the center of the preBötC (Ballanyi and Ruangkittisakul 2009). We used the established opioid sensitivity of the preBötC (Gray et al. 1999; Feldman and Del Negro 2006; Feldman et al. 2013) to verify that rhythmic oscillations in such slices are inspiratory‐related. Our analyses indicated that the preBötC center in Dbx1 reporter mice (at P0‐4) is located 0.4 mm caudal to the VII nucleus. This enabled us to map the distribution of fluorescent Dbx1 neurons in this functional network within the context of other brainstem areas and the rostral cervical spinal cord. The resources we provide will facilitate cellular‐level studies of respiratory rhythmogenesis in calibrated slice preparations from reporter mouse models in vitro.

## Materials and Methods

### Ethical approval

All experiments on live brain slices were performed using procedures conforming to ethical standards (Drummond 2009) and were approved by the Institutional Animal Care and Use Committee at The College of William & Mary in compliance with the U.S. National Institutes of Health, Office of Laboratory Animal Welfare guidelines.

### Mouse strains

We used mice that express Cre recombinase fused to the tamoxifen‐sensitive estrogen receptor (CreER^T2^) in cells expressing *Dbx1*, that is, *Dbx1*^*CreERT2*^ (Hirata et al. 2009). *Dbx1*^*CreERT2*^ mice were mated with *floxed* reporter mice in which the *Rosa26* locus was modified by targeted insertion of a *loxP*‐flanked STOP cassette followed by tandem dimer (td) Tomato (*Rosa26*^*tdTomato*^, strain name: B6;129S6‐*Gt(ROSA)26Sor*^*tm9(CAG‐tdTomato)Hze*^/J, Jax No. 007905; Madisen et al. 2010). Heterozygous *Dbx1*^*+/CreERT2*^ mice were bred in‐house using a CD‐1 background mouse strain. *Rosa26*^*tdTomato*^ mice were maintained as a homozygous line with C57BL/6J background. Animal genotypes were verified via real‐time polymerase chain reaction using primers for *Cre* and red fluorescent protein (Transnetyx, Cordova, TN).

**Figure 1.**
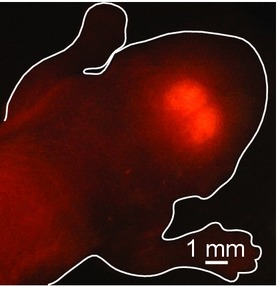
Native tdTomato fluorescence visible through the skin of a living P0 Dbx1 reporter (*Dbx1*^*ERC*^^*reT2*^*; Rosa26*^*tdTomato*^) mouse under epifluorescence stereomicroscopy. Tracing shows the contours of the animal.

Pregnancies were timed and monitored with embryonic day 0.5 defined 12 h after the initial mating. We administered tamoxifen (25 mg·kg^−1^ body mass) via oral gavage to pregnant *Dbx1*^*+/CreERT2*^ dams at embryonic day 10.5 to cause Cre‐Lox recombination. *Dbx1*^*+/CreERT2*^*; Rosa26*^*tdTomato*^ offspring mice were typically born after 20 days of gestation. Their screening was facilitated by the fact that fluorescent protein expression was visible through the skin and skull in ~50% of the pups at birth using a standard stereomicroscope equipped with epifluorescence and a rhodamine filter cube (Fig. 1). In total, we studied 15 Dbx1 reporter mouse pups and 13 wild‐type littermates.

### Brainstem dissection

Dbx1 reporter mouse pups and wild‐type littermates were anesthetized at postnatal days 0–4 (P0‐4) by immersion in crushed ice until disappearance of the tail pinch response. After isolation within 1–2 min, the neuraxis from the pons to the rostral cervical spinal cord was transferred to a dish filled with superfusate containing (in mmol·L^−1^): 124 NaCl, 3 KCl, 1.5 CaCl_2_, 1 MgSO_4_, 25 NaHCO_3_, 0.5 NaH_2_PO_4_, and 30 dextrose, equilibrated with carbogen, a mixture of 95% O_2_ and 5% CO_2_ to establish a pH of 7.4. After removing the arachnoidea, the neuraxis of P0‐4 mice was used for electrophysiological experiments, which were performed in the Del Negro laboratory at The College of William & Mary. Anatomical analyses of chemically fixed preparations from P0 or P4 mice in the Ballanyi laboratory at the University of Alberta.

### Neuroanatomy

First, we generated a brainstem atlas for Dbx1 reporter mice as previously done by us for Wistar/Sprague–Dawley rats (Ruangkittisakul et al. 2006) and C57BL/6 mice (Ruangkittisakul et al. 2011). In the latter study, the rostrocaudal extension of respiratory brainstem marker areas, but not the brainstem size, increased significantly in the C57BL/6 mice between P0 and P6 thus requiring the generation of two reference atlases. We assumed that this is also the case for Dbx1 reporter mice and therefore analyzed brainstems from P0 and P4 animals. We focused on this age range because imaging studies are optimal at early postnatal ages (P0‐4). To generate atlases for both P0 and P4 animals, the isolated neuraxis was transferred to phosphate buffer, that is, a 1:2 mixture of 0.1 mol·L^−1^ NaH_2_PO_4_ and 0.1 mol·L^−1^ Na_2_HPO_4_ in distilled water, pH 7.2, with 4% (by weight) paraformaldehyde added. After more than 12 h of storage at 4°C, the neuraxis was immersed in formaldehyde‐free phosphate buffer with 4% agar melted at 50°C. Following solidification of agar, the brainstem was transected caudal to rostral in cold buffer solution using a vibrating microtome (Leica VT1000s; Leica Microsystems, Richmond Hill, ON, Canada). For the first four Dbx1 reporter mouse brainstems, section thickness was 25 *μ*m; alternating sections were analyzed via thionin staining or native tdTomato fluorescence as described below. For a higher yield of complete sections, their thickness was increased to 50 *μ*m for four other Dbx1 reporter mouse brainstems from which sections were kept in phosphate buffer for tdTomato fluorescence imaging. Brainstem sections from seven wild type littermate mice were also cut at 50 *μ*m thickness for staining with thionin.

Thionin staining of cellular nucleic acid was used for bright‐field imaging of brainstem structures. Sections were mounted and dried on glass slides, then rehydrated in 70% ethanol (2 min), 50% ethanol (3 min), distilled water (3 min), followed by 40 sec immersion in thionin solution, that is, a 4:6:1 mixture of 0.1 mol·L^−1^ sodium acetate trihydrate, 0.1 mol·L^−1^ acetic acid and 1% (by weight) thionin acetate. Sections were then rinsed twice in distilled water and washed in 50% ethanol (2 min), 95% ethanol (1 min), and twice with 100% ethanol (1 min each). After clearing sections two times in xylene (3–4 min each), they were mounted and digitally photographed (PL‐A642‐1.3 Megapixel; PixeLINK, Ottawa, ON, Canada) at 32× magnification through a stereomicroscope with oblique bright‐field illumination. Native tdTomato fluorescence was imaged using 559 nm laser excitation and a Fluoview FV1000 confocal microscope (Olympus, Markham, ON, Canada) equipped with 4× (NA = 0.28) and 20× (NA = 1.0) objectives and a 575–675 nm band‐pass emission filter. Adobe Photoshop CS6 (Adobe Systems, San Jose, CA) was applied to merge, crop, enhance contrast, and adjust the background intensity of images. The rostrocaudal location of each section was expressed as the distance (in mm) from the caudal border of the VII motor nucleus (VII_c_) as in our earlier studies (Ruangkittisakul et al. 2006, 2008, 2011). The Dbx1 reporter mouse brainstem atlases are available online as Supporting Information.

### preBötC determination in calibrated sandwich slices

**Figure 2.**
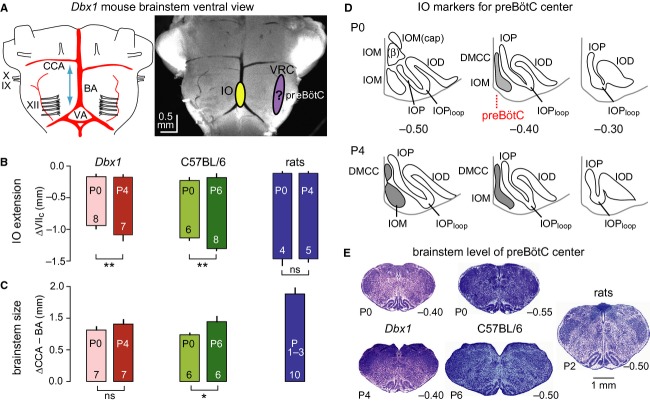
Comparative analyses of positions of inferior olive (IO), brainstem dimension, and preBötC location in Dbx1 reporter mice, C57BL/6 mice, and Wistar/Sprague–Dawley rats. (A) Ventral brainstem of a postnatal day 4 (P4) *Dbx1*^*ERC*^^*reT2*^*; Rosa26*^*tdTomato*^ mouse. BA, basilar artery; CCA, caudal cerebellar artery; IO, inferior olive; IX, glossopharyngus nerve; preBötC, pre‐Bötzinger complex; VA, vertebral artery; VRC, ventral respiratory column; X, vagus nerve; XII, hypoglossal nerve. The approximate positions of the medial IO (IOM) and preBötC within the rostrocaudal ventral respiratory column are projected onto the brainstem photograph. (B) IO extension referred to the distance from the caudal end of facial motor (VII) nucleus, VII_c_. Vertical bars show mean (±SD) of the rostral and caudal borders of the IO for each strain and species, as well as age. Asterisk and double asterisks indicate statistical significance at *P *<**0.05 and *P *<**0.01, respectively. Numerals indicate brainstems analyzed. (C) Brainstem dimension quantified as the distance from the mean value of the left and right CCA to the point at which the BA bifurcates to form the VAs. (D) Subgroups of the IO in transverse sections of P0 and P4 Dbx1 reporter mouse brainstems, which mark the location of the preBötC center (see red dotted line). Other abbreviations: IOD, dorsal IO; IOP, principal IO; IOP_loop_, lateral loop of IOP; DMCC, dorsomedial cell column of IO; IOM (ß), ß subgroup of IOM; IOM (cap), dorsal cap subgroup of IOM. We hypothesize that the preBötC center colocates with the level where the DMCC joins with the medial aspect of the IOM to form a vertical stalk. (E) Characteristic histological (thionin‐stained) brainstem sections at the presumed center of the preBötC. Calibration bar on right applies to all images. Data from C57BL/6 mice and newborn Wistar/Sprague–Dawley rats in (B) and thionin‐stained sections in (E) are taken from our previous studies Ruangkittisakul et al. (2006, 2011), respectively.

We developed calibrated slices in newborn rats to identify their necessary and sufficient thicknesses and borders for generation of preBötC rhythm (Ruangkittisakul et al. 2008). In newborn rats, two inspiratory active slices can be obtained from one brainstem provided their joint border is 100 *μ*m (or less) from the preBötC center (Ballanyi and Ruangkittisakul 2009). Thus, we planned here to vary the level of this joint border when cutting the sandwich slices from Dbx1 reporter mice, and then plot the position of the slice borders (in the parasagittal plane, with respect to distance from VII_c_) along with an index of their rhythmicity to indicate the brainstem level of the preBötC center (Ballanyi and Ruangkittisakul 2009). Based on a comparison of anatomical brainstem data from various animal species, we previously hypothesized that the preBötC center colocates with the brainstem level at which the principal loop of the IO (IOP_loop_) is fully developed while the medial IO (IOM) shows a sharp dorsomedial stalk‐like expression (Ruangkittisakul et al. 2011; Fig. 2). Here, we again used this anatomical landmark to estimate the common border for sandwich slices.

We tested whether the *μ*‐opioid receptor agonist DAMGO ([D‐Ala2, N‐MePhe4, Gly‐ol]‐enkephalin) blocked sandwich slice rhythms. *μ*‐opioids directly inhibit preBötC networks, but not networks of the neighboring parafacial respiratory group/retrotrapezoid nucleus (pFRG/RTN; Janczewski et al. 2002; Feldman and Del Negro 2006; Ballanyi and Ruangkittisakul 2009; Feldman et al. 2013).

To generate sandwich slices the isolated neuraxis was glued rostral side down to a cutting block that was then fixed on a vibrating microtome (Microm HM‐650‐V; Thermo‐Fisher Scientific, Waltham, MA) in a dish filled with superfusate (for composition, see above). We cut 100–150 *μ*m thick prerhythmic transverse sections from caudal to rostral while assessing respiratory brainstem marker areas. This enabled us to gage the appropriate level for cutting two consecutive 500 *μ*m thick sandwich slices containing (more or less) preBötC tissue for subsequent electrophysiological recording.

In four cases, the cutting level of the last prerhythmic slice was too rostral for generating two 500 *μ*m slices, so instead we cut two 250 *μ*m thick slices (*n* = 2) or one 250 *μ*m slice and one 500 *μ*m slice (*n* = 2). The entire sample consisted of ten 500 *μ*m thick and four 250 *μ*m thick slices from seven *Dbx1* mice as well as ten 500 *μ*m thick and two 250 *μ*m thick slices from six wild‐type littermates. Because the findings from 500 and 250 *μ*m slices were similar, the data were pooled. After cutting the sandwich slice pair in each experiment, we obtained one additional 100–150 *μ*m thick postrhythmic slice to further analyze brainstem marker areas.

Slices were mounted with the preBötC‐containing surface upward in a 2 mL recording chamber and exposed to carbogenated superfusate at a flow rate of 4 mL·min^−1^ maintained at 27–29°C with a heating unit (TC‐324B; Harvard Apparatus, Holliston, MA). Superfusate K^+^ was raised from 3 to 9 mmol·L^−1^ at the start of recording for sustained inspiratory‐related oscillations (Ballanyi and Ruangkittisakul 2009; Funk and Greer 2013). Superfusate‐filled suction electrodes with 100–200 *μ*m outer diameter tips were placed on the surface of the ventrolateral medulla to monitor preBötC population activity. A further electrode was used in eight of the 13 sandwich slice pairs to test for inspiratory‐related rhythmic activity in the hypoglossal (XII) motor nucleus. Suction electrodes were connected to the high input‐impedance head stage of a differential amplifier with a gain of 20,000 and a 0.3–1 kHz band‐pass filter (Dagan EX1, Minneapolis, MN). Signals were digitally acquired at 4 kHz using a 16‐bit analog‐to‐digital converter (PowerLab, ADInstruments, Colorado Springs, CO) and displayed in raw (AC‐coupled) and smoothed (root‐mean‐square) forms.

Sandwich slices as well as pre‐ and postrhythmic slices were chemically fixed as described above for more than 12 h, then washed in phosphate buffer (2 min), stained in thionin solution (40 sec), washed in 50% ethanol (4 min), and phosphate buffer (2 min) and finally photographed. We determined sandwich slice borders by comparing thionin‐stained surfaces with the atlases. The mean thickness was 370 ± 77 *μ*m for 500 *μ*m thick slices (*n* = 20) and 138 ± 59 *μ*m for 250 *μ*m thick slices (*n* = 6). These values were inconsistent with physical slice thickness determined by cutting a horizontal strip of the ventrolateral medulla, which measured 541 ± 41 *μ*m (500 *μ*m thick slices, *n* = 6) and 268 ± 7 *μ*m (250 *μ*m thick slices, *n* = 4). The underestimation of slice thicknesses by comparison to the atlases may be partly attributable to tissue lost during vibratome cutting or the penetration of thionin into the slice, which also stains deeper structures to cause an error of 50–70 *μ*m in estimating the surface. Alternatively, the imprecision regarding slice thickness may be due to the fact that the atlases are accurate only by up to 50 *μ*m due to sectioning thickness. As a result, the mean values between sandwich slices and their pre‐/postrhythmic slices are used for plotting slice borders. Consistent with the imprecision reported above, the borders between consecutive sandwich slices are separated by a gap of 50 ± 23 *μ*m.

### Salts and chemicals

Sources for chemicals were as follows: Sigma Aldrich (St. Louis, MO) for DAMGO (1 mmol·L^−1^ stock in distilled water), tamoxifen (dissolved at 10 mg·mL^−1^ in corn oil), agar, NaH_2_PO_4_, Na_2_HPO_4_, thionin acetate, and paraformaldehyde; Commercial Alcohol (Brampton, ON, Canada) for ethanol; Anachemia Chemicals (Rouses Point, NY) for xylene; Merck Millipore (Etobicoke, ON, Canada) for Entellen^®^ and Thermo‐Fisher Scientific for superfusate constituents.

### Data analysis

Inspiratory burst rate was analyzed for 2–4 min at steady state. We measured burst duration as the total elapsed time when the signal exceeded, to when it fell below, 10% of peak amplitude (ClampFit software; Molecular Devices, Chicago, IL). Mean values are reported with standard error for electrophysiological data, whereas histological means are reported with standard deviation. Statistical significance was determined using One‐way ANOVA with Tukey posttest or unpaired *t*‐test as appropriate (Prism Graphpad Software, La Jolla, CA). A single asterisk (*) reflects *P *<**0.05; Double asterisk (**) reflects *P *<**0.01. Mean values and significance values are not given in the text of the Results section, if evident in the illustrations and graphs.

## Results

### Marker nuclei of the respiratory brainstem

As a precondition for generating atlases to produce calibrated rhythmic slices for preBötC identification, we determined the rostrocaudal constancy of respiratory brainstem marker areas in thionin‐stained sections. We firstly found that the IO in Dbx1 reporter mice is smaller at P0 (*n* = 8) versus P4 (*n* = 7) and smaller than in C57BL/6 mice and Wistar/Sprague–Dawley rats (Fig. 2, Table 1). These differences in the IO extension were reflected by a smaller brainstem size in Dbx1 reporter mice, which was quantified as the distance between the caudal cerebellar artery and the point of bifurcation of the basilar artery (Fig. 2A–C, Table 1). Putting aside its neurophysiological role in sensorimotor integration, the IO can serve as a marker structure for comparative analysis of the respiratory brainstem because it consists of various subgroups that change their shape as a function of the caudal to rostral position. For example, at caudal brainstem levels, the medial IO (IOM) is divided into a, b, *β*, and dorsal cap subgroups. More rostrally, subgroups a and b join together and are referred to as the IOM, while the dorsal cap disappears and the *β* subgroup is displaced by another subgroup called the dorsomedial cell column (DMCC; Whitworth and Haines 1986; Li et al. 2001). Our previous study revealed that the level at which the DMCC joins the remaining IOM subgroups to form a narrow dorsomedial stalk, together with a fully developed IOP_loop_, colocates with the preBötC center in newborn Wistar/Sprague–Dawley rats (Ruangkittisakul et al. 2008). We hypothesized that this is also the case in C57BL/6 mice (Ruangkittisakul et al. 2011) and Dbx1 reporter mice studied here (Fig. 2D and E). In Dbx1 reporter mice, this particular brainstem level is located between 0.35 and 0.40 mm caudal to VII_c_, whereas it is located 0.50 mm caudal to VII_c_ in both newborn Wistar/Sprague–Dawley rats and P0 C57BL/6 mice (0.55 mm for P6 C57BL/6 mice; Fig. 2E).

**Table 1.** Comparison of IO and brainstem dimension between Dbx1 reporter mice, C57BL/6 mice, and Wistar/Sprague–Dawley rats.

	Distance (mm) from VII_c_	vs. P0 Dbx1	vs. P4 Dbx1
IO caudal border			
P0 Dbx1	−0.93 ± 0.07 (*n* = 8)		
P4 Dbx1	−1.10 ± 0.08 (*n* = 7)	^**^	
P0 C57BL/6	−1.13 ± 0.05 (*n* = 6)	^**^	ns
P6 C57BL/6	−1.30 ± 0.04 (*n* = 8)	^**^	^**^
P0 WS‐SD	−1.48 ± 0.09 (*n* = 4)	^**^	^**^
P4 WS‐SD	−1.47 ± 0.06 (*n* = 5)	^**^	^**^
IO rostral border			
P0 Dbx1	−0.17 ± 0.04 (*n* = 8)		
P4 Dbx1	−0.18 ± 0.04 (*n* = 7)	ns	
P0 C57BL/6	−0.23 ± 0.05 (*n* = 6)	ns	ns
P6 C57BL/6	−0.18 ± 0.06 (*n* = 8)	ns	ns
P0 WS‐SD	−0.13 ± 0.03 (*n* = 8)	ns	ns
P4 WS‐SD	−0.13 ± 0.03 (*n* = 8)	ns	ns
IO extension			
P0 Dbx1	0.76 ± 0.07 (*n* = 8)		
P4 Dbx1	0.92 ± 0.07 (*n* = 7)	^**^	
P0 C57BL/6	0.89 ± 0.10 (*n* = 6)	^*^	ns
P6 C57BL/6	1.12 ± 0.07 (*n* = 8)	^**^	^**^
P0 WS‐SD	1.35 ± 0.06 (*n* = 4)	^**^	^**^
P4 WS‐SD	1.33 ± 0.08 (*n* = 5)	^**^	^**^
BA to CCA distance			
P0 Dbx1	1.08 ± 0.07 (*n* = 7)		
P4 Dbx1	1.20 ± 0.10 (*n* = 7)	ns	
P0 C57BL/6	0.98 ± 0.04 (*n* = 6)	ns	ns
P6 C57BL/6	1.26 ± 0.11 (*n* = 6)	ns	ns
P0‐4 WS‐SD	1.84 ± 0.13 (*n* = 10)	^**^	^**^

A single asterisk *means *P* < 0.05, double asterisk **means *P* < 0.01, ns means not significant.

**Figure 3.**
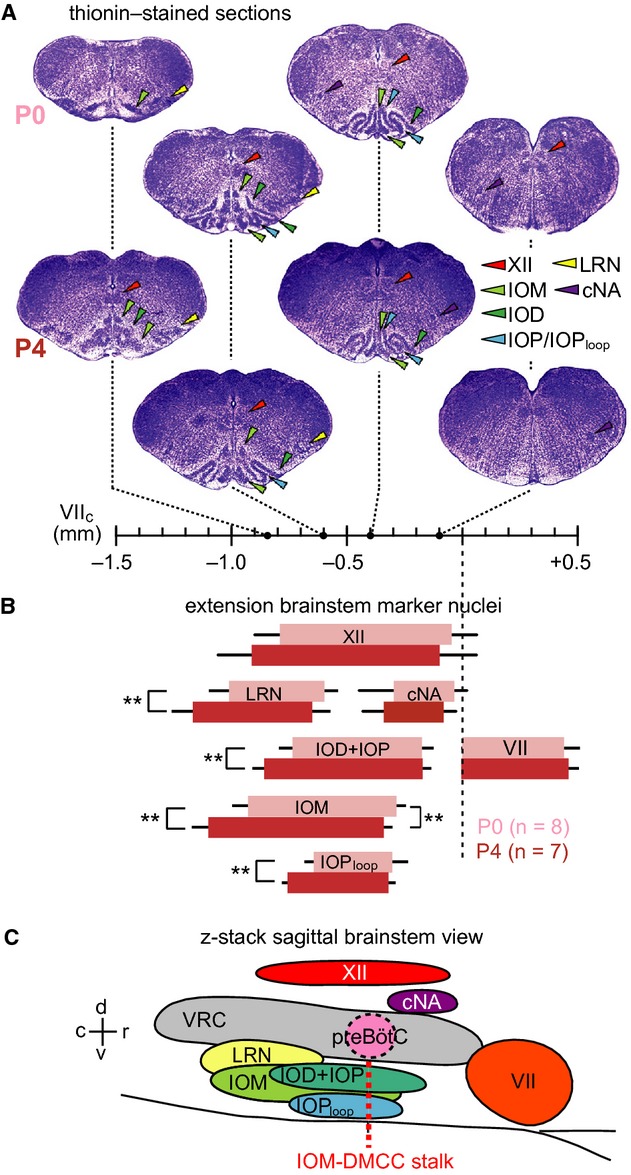
Relative position of respiratory marker nuclei in the brainstem of Dbx1 reporter mice. (A) Thionin‐stained transverse sections from the P0 and P4 reference brainstem atlases (see Supporting Information). Broken lines indicate rostrocaudal position along the abscissa referred to VII_c_. (B) Bars indicate mean ± SD of the rostrocaudal location and extension of marker sites and nuclei, specifically XII, LRN, cNA, VII, IOD, IOP, IOM, and IOP_loop_. Light and dark bars represent data from eight P0 and seven P4 mice, respectively. B has the same abscissa as in (A) Statistical significance was determined for caudal and rostral borders of marker nuclei in P0 versus P4 mice. Asterisk and double asterisks indicate statistical significance at *P *<**0.05 and *P *<**0.01, respectively. (C) Schematic parasagittal view of respiratory brainstem sites and marker nuclei. Red dotted line indicates the location of the preBötC center.

We also analyzed the extension of the other major IO subnuclei and further marker areas in the above eight P0 and seven P4 brainstems (Fig. 3, Table 2). Specifically, these areas were the IOM, the dorsal inferior olive (IOD), the principal inferior olive (IOP), and its loop (IOP_loop_), the VII, XII, and compact division of the ambiguus (cNA) motor nuclei, as well as the lateral reticular nucleus (LRN). No differences were found for the extension of the motor nuclei, whereas the LRN extended more caudally at P4. The IOD+IOP, IOM and IOP_loop_ also extended slightly more caudally at P4, whereas the rostral IOM extended more rostrally at P0 (Table 2).

**Table 2.** Rostrocaudal borders and extension of brainstem marker nuclei defined as distance from caudal end of VII (mm).

	P0 Dbx1	P4 Dbx1	P0 vs. P4
XII	(*n* = 8)	(*n* = 7)	
Caudal border	−0.79 ± 0.11	−0.90 ± 0.16	ns
Rostral border	−0.05 ± 0.11	−0.10 ± 0.16	ns
Extension	0.74 ± 0.10	0.81 ± 0.08	ns
LRN	(*n* = 8)	(*n* = 7)	
Caudal border	−1.01 ± 0.09	−1.16 ± 0.09	^**^
Rostral border	−0.60 ± 0.05	−0.65 ± 0.08	ns
Extension	0.41 ± 0.04	0.51 ± 0.06	^**^
IOM	(*n* = 8)	(*n* = 7)	
Caudal border	−0.93 ± 0.07	−1.09 ± 0.07	^**^
Rostral border	−0.28 ± 0.04	−0.34 ± 0.03	^**^
Extension	0.64 ± 0.07	0.75 ± 0.07	^**^
IOD + P	(*n* = 8)	(*n* = 7)	
Caudal border	−0.73 ± 0.05	−0.85 ± 0.06	^**^
Rostral border	−0.17 ± 0.04	−0.18 ± 0.04	ns
Extension	0.57 ± 0.04	0.68 ± 0.06	^**^
IOP_loop_	(*n* = 8)	(*n* = 7)	
Caudal border	−0.64 ± 0.04	−0.75 ± 0.03	^**^
Rostral border	−0.30 ± 0.06	−0.32 ± 0.03	ns
Extension	0.34 ± 0.06	0.43 ± 0.03	^**^
cNA	(*n* = 4)	(*n* = 4)	
Caudal border	−0.39 ± 0.07	−0.32 ± 0.10	ns
Rostral border	−0.09 ± 0.05	−0.09 ± 0.08	ns
Extension	0.30 ± 0.08	0.23 ± 0.03	ns
VII	(*n* = 8)	(*n* = 7)	
Caudal border	0	0	
Rostral border	0.45 ± 0.06	0.46 ± 0.04	ns
Extension	0.45 ± 0.06	0.46 ± 0.04	ns

A single asterisk *means *P* < 0.05, double asterisk **means *P* < 0.01, ns means not significant.

**Figure 4.**
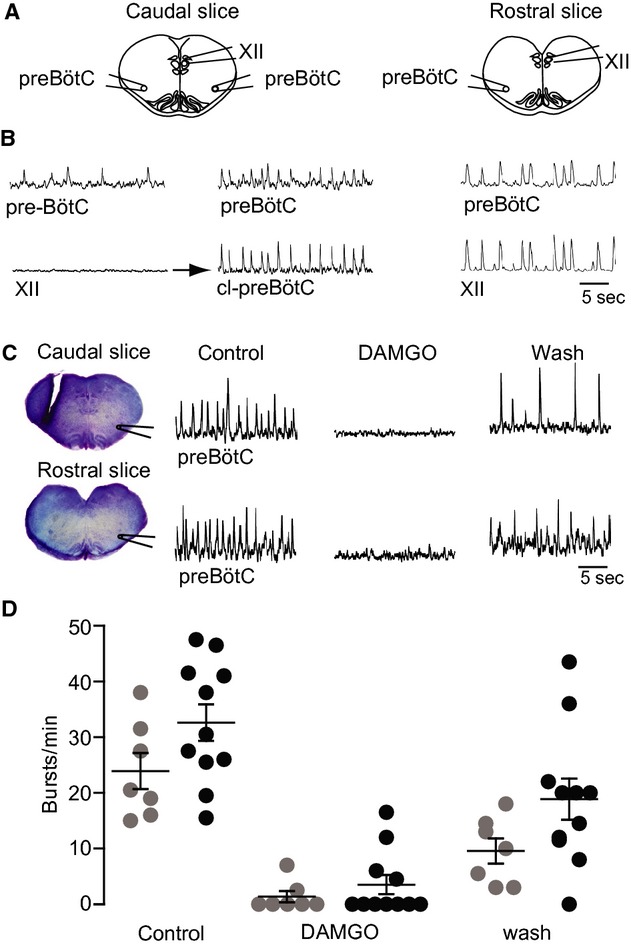
Opioid sensitivity of inspiratory rhythms in “sandwich” slice pairs from a single brainstem. (A) Schematics of adjacent sandwich slices and suction electrode positions. In some experiments the suction electrode in the XII nucleus was repositioned to record the contralateral preBötC, that is, cl‐preBötC. (B) Electrodes were initially positioned to record the preBötC area and the XII nucleus in a sandwich slice pair. Since the caudal slice showed no XII activity, the electrode was moved. Synchronous oscillations were present in the preBötC bilaterally. Right traces show synchronous preBötC and XII nucleus activity in the rostral slice of the pair. (C) In different sandwich slice pair, with the anterior border 0.40 mm and the posterior border 0.35 mm caudal to VII_c_, respectively, bath‐application of 1 *μ*mol·L^−1^ DAMGO reversibly abolished preBötC rhythm which partially recovered upon washout. (D) Scatter plot of burst rates in caudal (gray circles) and rostral (black circles) in superfusate (control), during bath‐application of DAMGO (1 *μ*mol·L^−1^) and washout.

**Figure 5.**
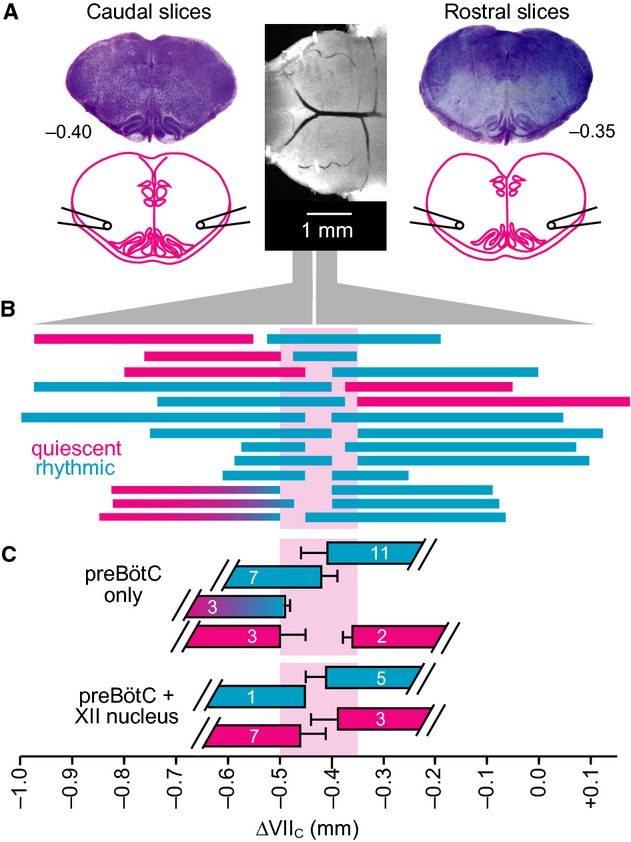
Electrophysiological recording of inspiratory rhythms in sandwich slices. (A) Central photograph shows the ventral brainstem prior to sectioning. Photographs in the left and right panels show consecutive “sandwich” slices exposed at the appropriate surface to obtain electrophysiological field recordings from the preBötC and XII. (B) Rostrocaudal borders of rhythmic and nonrhythmic sandwich slices. Abscissa at bottom shows distance relative to VII_c_. Thirteen slice pairs are illustrated: cyan represents respiratory active slices, whereas magenta indicates quiescent slices. Cyan‐to‐magenta blended bars reflect slices that started out rhythmically active but progressively ceased rhythmic activity over 30–70 min. (C) Mean (±SD) borders of rhythmic and arrhythmic preBötC field recordings are shown with corresponding mean (±SD) borders for slices with active or silent XII field recordings.

In summary, these findings of age‐dependent differences of respiratory marker areas in P0 versus P4 *Dbx1* mice confirmed the need for generating a brainstem atlas for both age groups. Also, the position of the full IO_loop_ and narrow stalk of the DMCC‐IOM area suggested that the preBötC center was located between 0.35 and 0.40 mm caudal to the VII motor nucleus (Figs. 2E, 3C), but note that physiological confirmation is demonstrated below (Figs. 4, 5).

### preBötC determination based on inspiratory‐related rhythms in sandwich slices

To identify the anterior–posterior borders of the preBötC, we measured respiratory rhythmogenic function and opioid sensitivity in contiguous pairs of sandwich slices while we intentionally varied their common border. Specifically, neuronal population activity in the ventrolateral medulla containing the VRC and the more or less exposed preBötC was monitored via suction electrodes positioned on the adjacent surfaces of the sandwich slice pairs from seven Dbx1 reporter mice and six wild‐type littermates. Slices with comparable borders generated similar behaviors, regardless of group, so the data were pooled. Eight of 13 slice pairs exhibited rhythm in both adjacent slices (Fig. 4A and B). However, in three cases the caudal slice rhythm subsided after 30–70 min of recording. The remaining five sandwich slice pairs exhibited rhythm only in either the rostral (*n* = 3) or caudal (*n* = 2) aspect. The mean burst rate after 30 min was 28.6 ± 6.1 min^−1^ for ten caudal slices and 32.6 ± 3.3 min^−1^ for eleven rostral slices (not significant, *P *=**0.56). We observed no indication of fictive sighs from caudal or rostral slices (Lieske et al. 2000; Ballanyi and Ruangkittisakul 2009). Burst duration measured 588 ± 72 ms (range 509–732 ms) for rostral slices and 675 ± 23 ms (range 634–713 ms) for caudal slices, and did not differ between both slice aspects as analyzed in three rhythmic sandwich slice pairs (*P *=**0.32).

For the eight sandwich pairs that showed rhythm in both adjacent slices, we monitored rhythms in the preBötC area and the XII motor nucleus simultaneously (Fig. 4A and B). Six of these pairs showed synchronized oscillations in the preBötC and the XII motor nucleus in either the caudal (*n* = 1) or the rostral slice (*n* = 5). However, we never detected activity in the XII motor nucleus of both adjoining rhythmic slices from the same brainstem. In nine of 10 slices with no XII motor nucleus activity, we moved the second suction electrode to the contralateral preBötC. In these nine slices, bilateral preBötC activity was synchronous (*n* = 4; Fig. 4A and B), asynchronous (*n* = 1), or unilateral (*n* = 3). One slice had experienced “rundown” and showed no further activity in the preBötC of either side. These results indicate that sandwich slices, which do not always show sustainable bilaterally synchronous activity in the preBötC as well as rhythmic activity in the XII nucleus, are compromised from a functional standpoint. Conventional slice preparations, which retain the preBötC in its entirety and XII nerve rootlets, typically exhibit bilaterally synchronous activity in the preBötC and further retain sufficient premotor neurons to spontaneously generate robust XII motor output (Greer et al. 1991; Funk and Greer 2013).

Bath‐application of DAMGO (1 *μ*mol·L^−1^) to seven caudal sandwich slices abolished rhythm in five cases and slowed it down in the other two. Altogether, mean burst rate decreased from 23.9 ± 3.3 min^−1^ to 1.4 ± 1.0 min^−1^ within ~10 min after the start of DAMGO exposure and recovered to 9.6 ± 2.2 min^−1^ after 15–20 min of washout (Fig. 4C and D). In eleven rostral slices, DAMGO stopped the rhythm in seven cases and slowed it down in the other four. Altogether, DAMGO decreased burst rate from 32.6 ± 3.3 min^−1^ to 3.6 ± 1.7 min^−1^, which recovered after 15–20 min to 18.9 ± 3.7 min^−1^ (Fig. 4C and D). As rhythmic bursting was attenuated by DAMGO in all slices, it very likely originates from more or less intact inspiratory preBötC circuits and not from the pFRG/RTN.

Offline histological analysis of thionin‐stained sandwich slice pairs allowed us to correlate the incidence of inspiratory rhythm with slice borders, and thus determine the anterior–posterior borders of the preBötC. The anterior border of caudal rhythmic slices (*n* = 7) was located 0.42 ± 0.03 mm caudal to VII_c_, whereas that for inactive caudal slices (*n* = 3) was located 0.50 ± 0.05 mm caudal to VII_c_ (Fig. 5A–C). Three caudal slices with the anterior border located 0.49 ± 0.01 mm caudal to VII_c_ were only transiently active. The mean posterior border of rostral rhythmic slices (*n* = 11) was 0.41 ± 0.05 mm caudal to VII_c_, whereas that of inactive rostral slices (*n* = 2) was 0.36 ± 0.02 mm caudal to VII_c_ (Fig. 5A–C). Synchronous preBötC and XII motor nucleus rhythms were recorded in one caudal slice whose anterior border was located 0.45 mm caudal to VII_c_ (*n* = 1), but not in the other seven slices with a very similar anterior border (0.46 ± 0.05 mm). Synchronous preBötC and XII motor nucleus rhythms were recorded in five rostral slices with the posterior border located 0.41 ± 0.04 mm caudal to VII_c_, but not in three other slices with a very similar posterior border (0.39 ± 0.05 mm; Fig. 5C). There was no correlation between the slice border and the rate of inspiratory rhythm or burst duration (data not shown).

These electrophysiological data indicate that the preBötC in Dbx1 reporter mice is centered ~0.43 mm caudal to VII_c_ and spans between 0.50 and 0.35 mm caudal to VII_c_.

### Fluorescent Dbx1 neuron distribution

**Figure 6.**
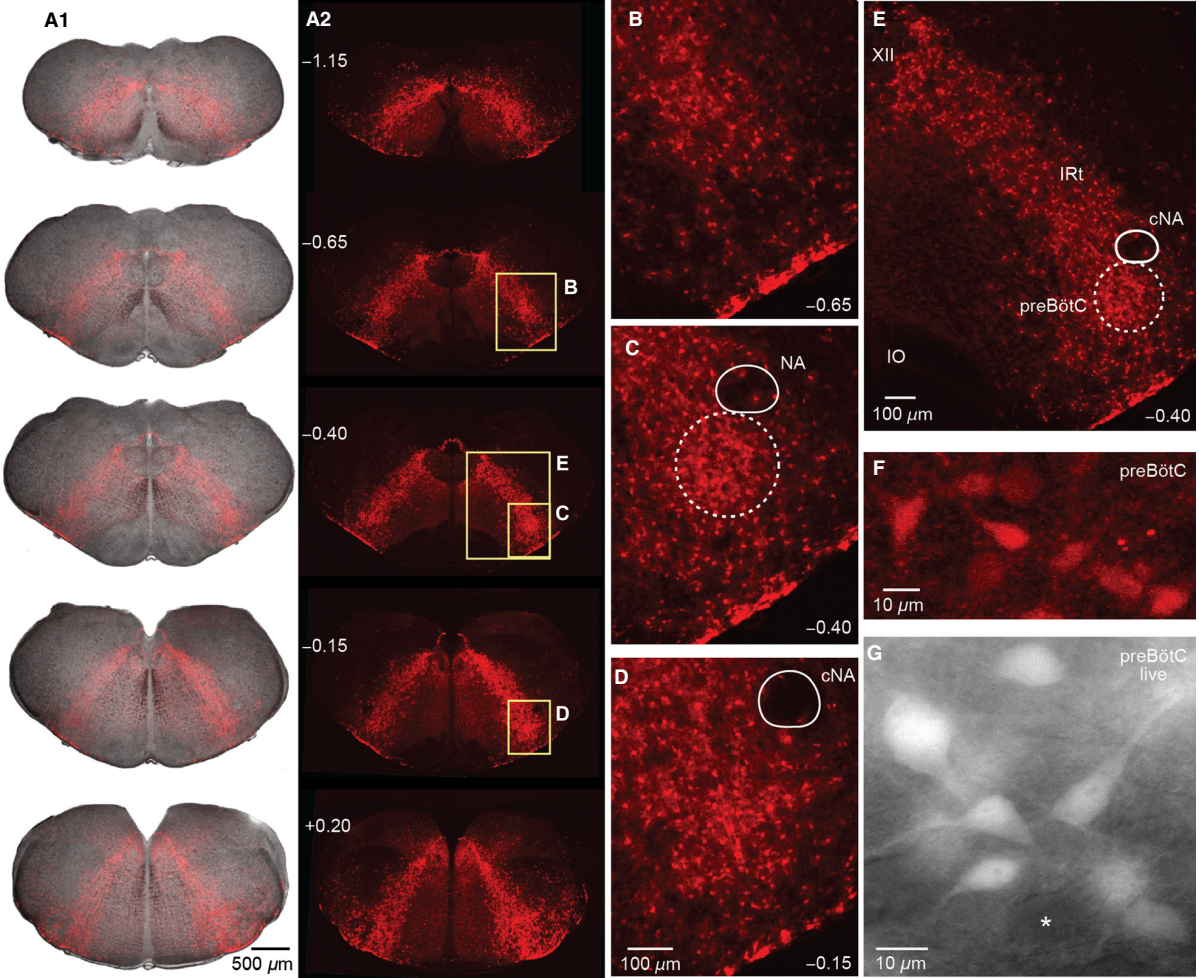
Distribution of Dbx1 neurons in transverse brainstem sections. (A) Overlay of bright field and tdTomato fluorescence images (A_1_) and fluorescence only (A_2_) from a fixed P4 Dbx1 reporter mouse brainstem at varied rostrocaudal levels indicated as distance from VII_c_. (B–E) Magnified inserts (see boxes) of the images in A_2_ show *Dbx1* neurons in the VRC caudal to (at −0.65 in (B), within (−0.40 in (C), and rostral to (−0.15 in D) the preBötC center. The ventrolateral slice surface shows a high‐density band of fluorescent cells and neuropil. The compact division of the nucleus ambiguus (cNA) is devoid of native fluorescence. (E) Shows higher density of Dbx1 neurons in the preBötC compared to the intermediate reticular formation (IRt). (F, G) Magnified view of Dbx1 preBötC neurons from a fixed brainstem section (F) and a live slice used for recording (G).

We analyzed transverse histological sections from the spinomedullary junction to the rostral end of VII motor nucleus. The somata of Dbx1 neurons form an inverted arched U‐shape that encompasses the VRC in the brainstem, with its thin‐layered vertex located dorsal to the central canal (Fig. 6A, e.g., −1.15 mm caudal to VII_c_). At brainstem levels where the XII motor nucleus is found, a dense array of Dbx1 neuron somata surrounds that nucleus and forms a distinct thin layer next to its dorsal border (Fig. 6A, e.g., −0.65 or −0.40 mm caudal to VII_c_). The ventrolateral slice surface contains Dbx1 cells that cluster within 30 *μ*m from the ventral surface (Fig. 6B–E). At brainstem levels rostral to the XII motor nucleus, the dorsal band of fluorescent cells becomes more compact and the inverted U‐shape transforms into a V‐shape (Fig. 6A). Diffuse low‐level fluorescence occupies the reticular formation medial to the band of Dbx1 neurons (Fig. 6A). The rostrocaudal distribution of Dbx1 neurons is uniform throughout the VRC with no apparent “hot spot” that colocates with the preBötC (compare ventrolateral area in Fig. 6C to those in Fig. 6B and D). Dbx1 neurons appear to be more numerous and densely arrayed in the VRC compared to the intermediate reticular formation, which is located between the XII motor nucleus and the preBötC and contains among other cell types inspiratory active XII premotor neurons (Koizumi et al. 2013), yet there is no apparent nuclear organization (Fig. 6B–E).

**Figure 7.**
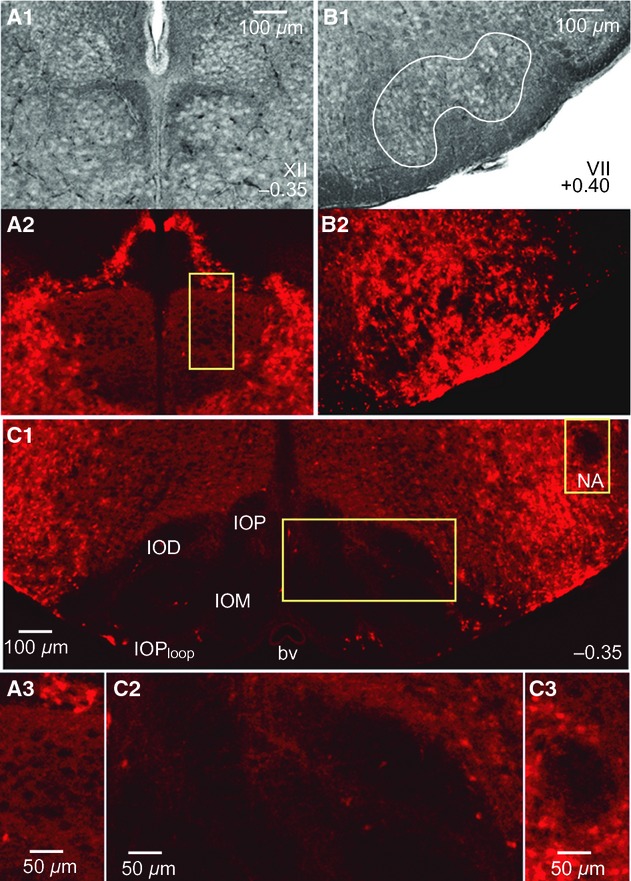
Lack of native tdTomato fluorescence in brainstem motor nuclei. (A) Bright field (A_1_) and corresponding fluorescence images (A_2_) of the XII nucleus at 0.35 mm caudal to VII_c_ from a P4 Dbx1 reporter mouse. Inset from A_2_ is shown in A_3_ at higher magnification. (B) Bright field (B_1_) and fluorescent view (B_2_) of the VII motor nucleus at 0.40 mm rostral to VII_c_. (C) Lack of native fluorescence in the IO and cNA of the same brainstem section as in (A). C_2_ and C_3_ show magnified images of the boxes highlighted in C_1_.

In fixed brainstem sections, Dbx1 preBötC neurons typically have 10–15 *μ*m diameter somata with stronger fluorescence than in the dendrites, for which only the proximal aspects are visible (Fig. 6F). Native tdTomato fluorescence reflects the neuronal morphology better in live slices used for recording (Fig. 6G), which may be experimentally advantageous for electrophysiological recording and imaging. Fluorescent Dbx1 neuron somata are not present in the LRN or in most of the motor nuclei (Fig. 7). In the XII motor nucleus, high magnification images show a lack of fluorescent motoneuron somata as black holes with a diameter of ~20 *μ*m that are embedded in diffuse low‐level fluorescence surrounding (Fig. 7A_1–3_). In the VII nucleus motoneurons do not express fluorescence, but tdTomato expression in neuropil is visible (Fig. 7B_1,2_). The parafacial region contains a great deal of respiration‐related neural circuitry (Smith et al. 1989; Mulkey et al. 2004; Dubreuil et al. 2008; Rose et al. 2009; Thoby‐Brisson et al. 2009; Pagliardini et al. 2011; Feldman et al. 2013) including an intermingled *Dbx1*‐derived population that is required to express expiratory related motor rhythms in vitro (Tupal et al. 2014a). Similarly, somata in IO subnuclei and the cNA motor nucleus are devoid of fluorescence, whereas a diffuse signal is visible between IO subgroups (Fig. 7C_1–3_). The P0 and P4 Dbx1 reporter mouse brainstem atlases are available (online) as Supporting Information.

## Discussion

We identified the preBötC location in Dbx1 reporter mice. Furthermore, we show the distribution of Dbx1 interneurons, which in hindbrain are predominantly glutamatergic and putatively respiratory rhythmogenic (Bouvier et al. 2010; Gray et al. 2010). These tools enable the generation of calibrated slices that expose the preBötC at one border for optically guided patch‐clamp recording or fluorescent (calcium) imaging, which will facilitate investigations of rhythmogenic preBötC neurons.

### preBötC identification in calibrated sandwich slices

The atlas for newborn Dbx1 reporter mice and functional assessment of sandwich slices determined the borders that are necessary and sufficient for preBötC slice rhythm, as previously shown for newborn rats slices (Ruangkittisakul et al. 2006, 2008). Two adjacent slices from one mouse brainstem can both show preBötC field rhythms provided their common section level is at – or close to – the middle of the block of tissue that transection experiments showed was necessary to preserve slice rhythmicity (see Fig. 5). These results suggest that the joint section of two rhythmically active sandwich slices is within 100 *μ*m of the preBötC center, as we previously showed in neonatal rats (Ballanyi and Ruangkittisakul 2009).

The inspiratory burst rate in conventional preBötC slices from newborn mice or rats, and also in newborn rat sandwich slices, is typically 5–15 min^−1^ (Ballanyi and Ruangkittisakul 2009). In contrast, the mean burst rates of 24–33 min^−1^ in both slice aspects are notably faster in Dbx1 reporter mice. Faster burst rates in the present context are probably not attributable to elevated K^+^ since both rat and mouse slices were exposed K^+^ concentrations as high as 9 mmol·L^−1^ in our prior work (Ruangkittisakul et al. 2006, 2011). In addition, the external Ca^2+^ concentration was 1.5 mmol·L^−1^ in the present context using Dbx1 reporters, whereas our prior work in C57BL/6 mice and Wistar/Sprague–Dawley rats utilized 1 mmol·L^−1^ Ca^2+^. The lower Ca^2+^ level generally accelerates slice rhythms (Ballanyi and Ruangkittisakul 2009), so disparity in extracellular Ca^2+^ cannot explain the present disparity in burst rates either.

It is possible that an overall reduction in connectivity, which results from sectioning the preBötC in the transverse plane, has the counterintuitive effect of accelerating the rhythm (see: Butera et al. 1999). Excitatory transmission in a coupled oscillator system can generally slow down the rhythm by augmenting the duration of both the active and quiescent parts of the cycle period; the net result being a slower rhythm. To diminish the coupling slightly can alleviate the coupling‐induced deceleration without breaking the entrainment that keeps the coupled oscillators unilaterally in sync (Del Negro et al. 2001).

Future studies will be required to resolve the mechanisms that underlie the disparity of inspiratory rhythms between Dbx1 reporter mice and newborn rats and C57BL/6 mice. Follow‐up studies may also determine whether both sandwich slice aspects generate rhythmic oscillations in physiological (3 mmol·L^−1^) superfusate K^+^ as in newborn rat slices (Ruangkittisakul et al. 2006, 2008, 2011).

Field preBötC rhythms could be bilaterally asynchronous with irregular burst activity. Furthermore, the rhythm in the XII nucleus was not measureable in all slices. Particularly in adjacent pairs of sandwich slices, the XII rhythm was only measurable in one slice aspect (never both). These findings are consistent with the proposal above that the sandwich slice procedure by its nature reduces excitatory connectivity overall. This reduction may then impact the inspiratory burst rate (accelerating it) and robustness of rhythm, while also desynchronizing bilateral coupling of the preBötC (Bouvier et al. 2010) and severing key inspiratory XII premotor neurons that ensure inspiratory‐related motor bursts in the XII nucleus and nerve (Koizumi et al. 2008, 2013).

We found for newborn rat sandwich slices that the caudal slice displayed in the ventrolateral area containing the preBötC a regular 3–15 min^−1^ burst rate and a burst duration of <1 sec. The corresponding rostral slice aspect showed a slightly slower (2–10 min^−1^) rhythm with similar burst duration. However, the rostral slice rhythm was intermingled with another oscillation at a rate of 5–10min^−1^ in which each burst lasted 2–5 sec in ~50% of cases (Ballanyi and Ruangkittisakul 2009). The latter long burst duration rhythms in rostral slices were enhanced by DAMGO, whereas that opioid blocked the short burst duration discharges in both sandwich slice aspects. Previous in vivo and in vitro studies established that preBötC inspiratory circuits are directly inhibited by opioids, whereas rhythms in the pFRG/RTN comprising a rhythmogenic expiratory center in newborn mammals persists or is even stimulated (Gray et al. 1999; Janczewski et al. 2002; Mellen et al. 2003; Feldman and Del Negro 2006; Onimaru et al. 2006; Ballanyi and Ruangkittisakul 2009; Feldman et al. 2013). As in our prior report (Ballanyi and Ruangkittisakul 2009), we hypothesize here that preBötC circuits generate rhythmic short bursts in both the rostral and caudal aspect of sandwich slices. The lack of DAMGO‐insensitive rhythms in the present context indicates that rhythmogenic pFRG/RTN neurons are not present in the brainstem areas of Dbx1 reporter mice nor retained in the sandwich slice pairs. Alternatively, the number of synchronously active pFRG/RTN neurons might be too small to generate a robust population signal studied here.

It is currently thought that the preBötC constitutes the inspiratory kernel, the long sought‐after “noeud vital” (Flourens 1858; Feldman 1986; Smith et al. 1991; Feldman et al. 2013). However, many studies (Guyenet and Wang 2001; Wang et al. 2001; Guyenet et al. 2002; Stornetta et al. 2003) – including our present results and the previous data on newborn rats (Ballanyi and Ruangkittisakul 2009) – indicate that preBötC is distributed, that is, the functionality of the preBötC network diminishes as more of it is cut off, or its constituent interneurons are ablated (Hayes et al. 2012; Wang et al. 2014). Evidence for the idea that the preBötC is a specialized region of the VRC was recognized several decades ago from in vivo brainstem lesion experiments (Feldman and Speck 1983; Feldman 1986). The present transections experiments help determine the rostrocaudal extension of the preBötC within the VRC. There were a sufficient number of cases with only one rhythmic sandwich slice to suggest that the preBötC extends 200 *μ*m (or less) as in newborn rats (Ruangkittisakul et al. 2008). Also in line with the latter study is our present finding that approximately 100 *μ*m of preBötC tissue is required for synchronized rhythms in the ventrolateral slice area, although those preBötC field rhythms may not show synchronized activity in the XII nucleus (also see: Kam et al. 2013, Wang et al. 2014). The graph in Figure 5 indicates that even 50 *μ*m of preBötC tissue might be able to generate rhythmic activity in field recordings of the preBötC networks. However rhythmic activity detected in field recordings does not represent a functionally intact preBötC, since that activity pattern is not always bilaterally in sync, and does not often couple to activity in the XII motor nucleus. Another caveat to keep in mind is that slice borders were estimated by reference to an atlas selected as most representative among various animals, but brainstem nuclei extensions do vary slightly from one animal to the next.

### Slice cutting strategies to expose preBötC interneurons

The brainstem atlas for Dbx1 reporter mice locates the preBötC within the heterogeneous VRC, among discernable marker nuclei. This tool provides a coordinate frame of reference to generate transverse slices that will facilitate visually targeted recordings of rhythmogenic inspiratory neurons as well as Dbx1 neurons outside the preBötC that may have auxiliary respiratory functions. Due to the early postnatal growth that particularly affects caudal structures such as the IO and its subnuclei, we produced an atlas for both P0 and P4 Dbx1 reporter mice (Supporting Information). Nevertheless, we propose that the preBötC center is located 0.4 mm caudal to VII_c_ for both ages. Our electrophysiology and opioid sensitivity data show that the critical extension of the preBötC was from 0.50 to 0.35 mm from VII_c_ and centered at 0.43 mm. However, since the atlas has an increment of 50 *μ*m, we estimate the preBötC center to be 0.40 mm from VII_c_. We argue that the preBötC position is equivalent at P0 and P4 because the IOP_loop_ in its entirety and the joint IOM‐DMCC, which we have established as reliable indicators of the preBötC center (Ruangkittisakul et al. 2011), are located in both age groups between 0.35 and 0.4 mm caudal to VII_c_.

The fact that the preBötC location in Dbx1 reporter mice is 100 *μ*m more rostral than in newborn rats is consistent with the smaller mouse brainstem. However, the preBötC location in Dbx1 reporter mice is also 100 *μ*m more rostral compared to C57BL/6 mice, which is not attributable to species‐related size disparity. The Dbx1 Cre‐driver mouse is bred on a CD‐1 outbred strain, which shifts the position of the preBötC more rostrally compared to the pure inbred C57BL/6 mouse. Although the absolute position is different, in both mouse strains the IO subnuclei are remarkably reliable indicators of the preBötC. This colocalization of the IO subnuclei and the preBötC holds for all rodent species and strains so far tested: Wistar/Sprague–Dawley rats (Ruangkittisakul et al. 2006), C57BL/6 mice (Ruangkittisakul et al. 2011), and now *Dbx1*^*CreERT2*^*; Rosa26*^*tdTomato*^ mice (this study). Although it has not be studied rigorously, the IO and subnuclei also appear to colocalize with the preBötC in cats, mole rats, and bats (Schwarzacher et al. 1995; Tupal et al. 2014b), and humans (Schwarzacher et al. 2011). Thus, the position of the IO and its subnuclei may also prove to be useful in identifying the preBötC in model organisms (e.g., hamsters, guinea pigs, rabbits, and other mouse strains) for which atlases are not yet available.

In the present context of Dbx1 reporter mice, to cut neonatal slices that optimally expose the preBötC at the rostral face we recommend a rostral border of −0.35 mm. This level corresponds to the emergence of the IOM as well as the presence of the IOD and the IOP_loop_. The IO and subnuclei are visible in bright field and can be inspected online during slice cutting using a stereomicroscope. The caudal border of the slice is a matter of investigator preference. A minimal slice thickness of 200 *μ*m retains the preBötC core and thus increases the probability of preBötC rhythmicity. However, we favor slice thickness of at least 400 *μ*m to optimally retain XII premotor neurons (Koizumi et al. 2008) and specifically Dbx1 neurons with XII premotor function (Wang et al. 2014), and thus boost the likelihood of recording inspiratory rhythms in the preBötC as well as XII motor output. In addition to visualizing marker nuclei in bright field during slice cutting, characteristic structures at the rostral face can be further inspected via epifluorescence. The IO and subnuclei will be dark, whereas the pattern of native fluorescence protein expression will be V‐shaped, with a recognizable dark oval that corresponds to the NA.

To cut neonatal Dbx1 reporter mouse slices that expose the preBötC at the caudal face we recommend a caudal border of −0.5 mm. This level corresponds to the IOM with an elongated dorsal tip, the lateral flat portion of the IOD, and a prominent (as opposed to minimally discernable) IOP_loop_. The NA should not be present at the caudal surface at this level. As above, we favor a slice thickness of at least 400 *μ*m to maximize the likelihood that the slice generates preBötC rhythms and XII motor output. Because IO structures extend farther caudal in the P4 mouse (Figs. 2, 3), age‐specific atlases will facilitate identification of these marker nuclei accordingly.

### Brainstem distribution of rhythmogenic Dbx1 interneurons

The rhythmogenic preBötC circuit consists of glutamatergic interneurons that further express neuropeptides and peptide receptors, which emerge from *Dbx1*‐expressing precursors during embryonic development (Bouvier et al. 2010; Gray et al. 2010). Cre‐dependent reporter systems enable the optical targeting of Dbx1 interneurons in physiological studies via native fluorescence expression (Madisen et al. 2010). However, Dbx1 neurons were distributed uniformly from the area lateral to the XII motor nucleus to ventrolateral medulla encompassing the intermediate reticular formation and VRC. These regions contain respiratory rhythmogenic circuits (Feldman and Del Negro 2006; Feldman et al. 2013), XII premotor neurons (Koizumi et al. 2008), and other oral‐motor circuits (Moore et al. 2013; Kleinfeld et al. 2014). Dbx1 neurons in the preBötC are not organized into a distinct nuclear structure. Targeting the preBötC requires cutting transverse slices wherein the preBötC is exposed at one face by visualizing marker nuclei using bright field or fluorescence microscopy.

We reported that multiple inspiratory active neurons, recorded selectively on the basis of native fluorescent protein expression in the ventrolateral aspect of preBötC slices, have discharge characteristics of putatively rhythmogenic type‐1 cells first described by Rekling and colleagues (Rekling et al. 1996; Rekling and Feldman 1998; Gray et al. 2010; Picardo et al. 2013). Using calibrated (sandwich) slices, it will be possible now to elucidate whether such Dbx1 neurons showing preinspiratory activity spiking patterns are found at a higher density within the rostrocaudal boundaries preBötC, which would be predicted if indeed type‐1 Dbx1 preBötC interneurons are key rhythm generators. This would explain why sandwich slices in Dbx1 reporter mice and Wistar/Sprague‐Dawley rats (Ballanyi and Ruangkittisakul 2009) do not show inspiratory bursting in the ventrolateral area unless they contain at least some portion of the rhythmogenic preBötC circuitry.

The use of Cre‐dependent reporter mice is becoming a ubiquitous tool for identifying and manipulating neural circuits. Recent Cre‐dependent reporters incorporate optogenetic tools in addition to fluorophores (Madisen et al. 2012). The variety and utility of floxed reporters will likely continue to expand, which further emphasizes the importance of our brainstem atlases that document the distribution of Dbx1 neurons in respiratory brainstem areas including the preBötC as well as the intermediate reticular formation and VRC.

## Conflict of Interest

The authors have no competing interests.

## Supplementary Material

**Figure S1**. Atlas of Dbx1 mouse (P0) medulla oblongata from a series of 50 μm thick thionin stained transverse sections. PDF

**Figure S2**. Atlas of Dbx1 mouse (P4) medulla oblongata from a series of 50 μm thick thionin stained transverse sections. PDF

**Figure S3**. Atlas of tdTomato fluorescence in Dbx1 mouse (P4) medulla oblongata shown as fluorescence image (right) and overlay with bright field image (left) from 50 μm thick transverse sections. PDF

**Figure S1**.

**Figure S2**.

**Figure S3**.
