# Supplementary material for: Identification of the pre‐Bötzinger complex inspiratory center in calibrated “sandwich” slices from newborn mice with fluorescent Dbx1 interneurons
Source: Physiol Rep. 2014 Aug 19;2(8):e12111. doi: 10.14814/phy2.12111 (PMC4246597; doi:10.14814/phy2.12111)
Supplement: Supplementary file 1 — Figure S1. Atlas of Dbx1 mouse (P0) medulla oblongata from a series of 50 μm thick thionin stained transverse sections. PDF [file phy2-2-e12111-s1.pdf]

Atlas of *Dbx1* mouse (P0) medulla oblongata

Ruangkittisakul A[1], Kottick A[2], Picardo MCD[2], Ballanyi K[1]*, Del Negro CA[2]*

[1] Department of Physiology, University of Alberta, Edmonton, AB, Canada

[2] Department of Applied Science, The College of William & Mary, Williamsburg, VA, USA

Abbreviations

AP	area postrema
IO	inferior olive
IOD	dorsal inferior olive
IOM	medial inferior olive
IOP	principal inferior olive
LRN	lateral reticular nucleus
NA	nucleus ambiguus, compact formation
NTB	nucleus of the trapezoid body
PD	pyramidal decussation
OBEX	Obex, the point where the central canal opens to the 4th ventricle
V4	4th ventricle
VII	facial nucleus
VIIdor	dorsal nucleus of the facial nucleus
VIImed	medial nucleus of the facial nucleus
VIIlat	lateral nucleus of the facial nucleus
XII	hypoglossal nucleus

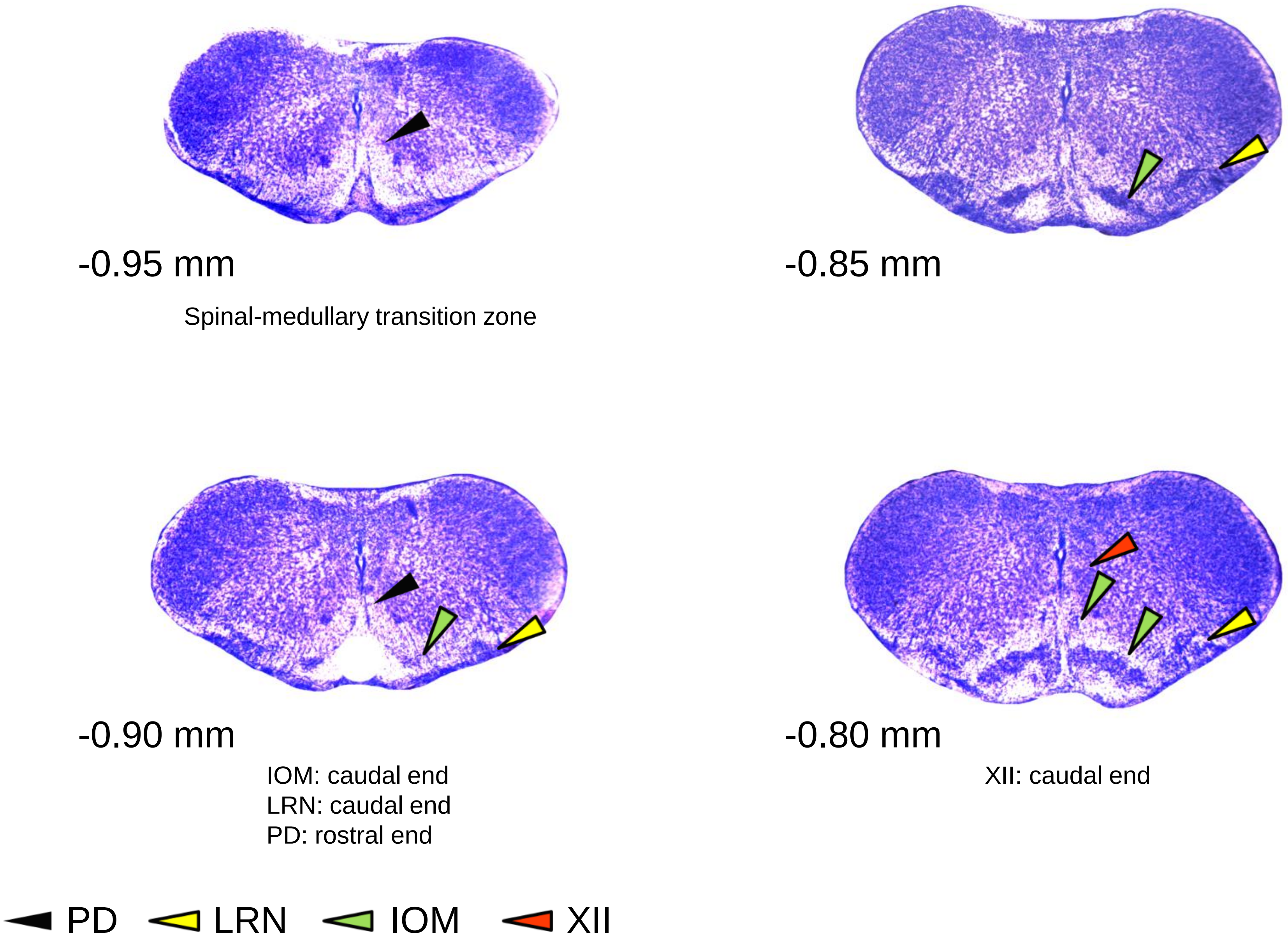
-0.95 mm
Spinal-medullary transition zone
-0.85 mm
-0.90 mm
IOM: caudal end
LRN: caudal end
PD: rostral end
-0.80 mm
XII: caudal end
PD
LRN
IOM
XII

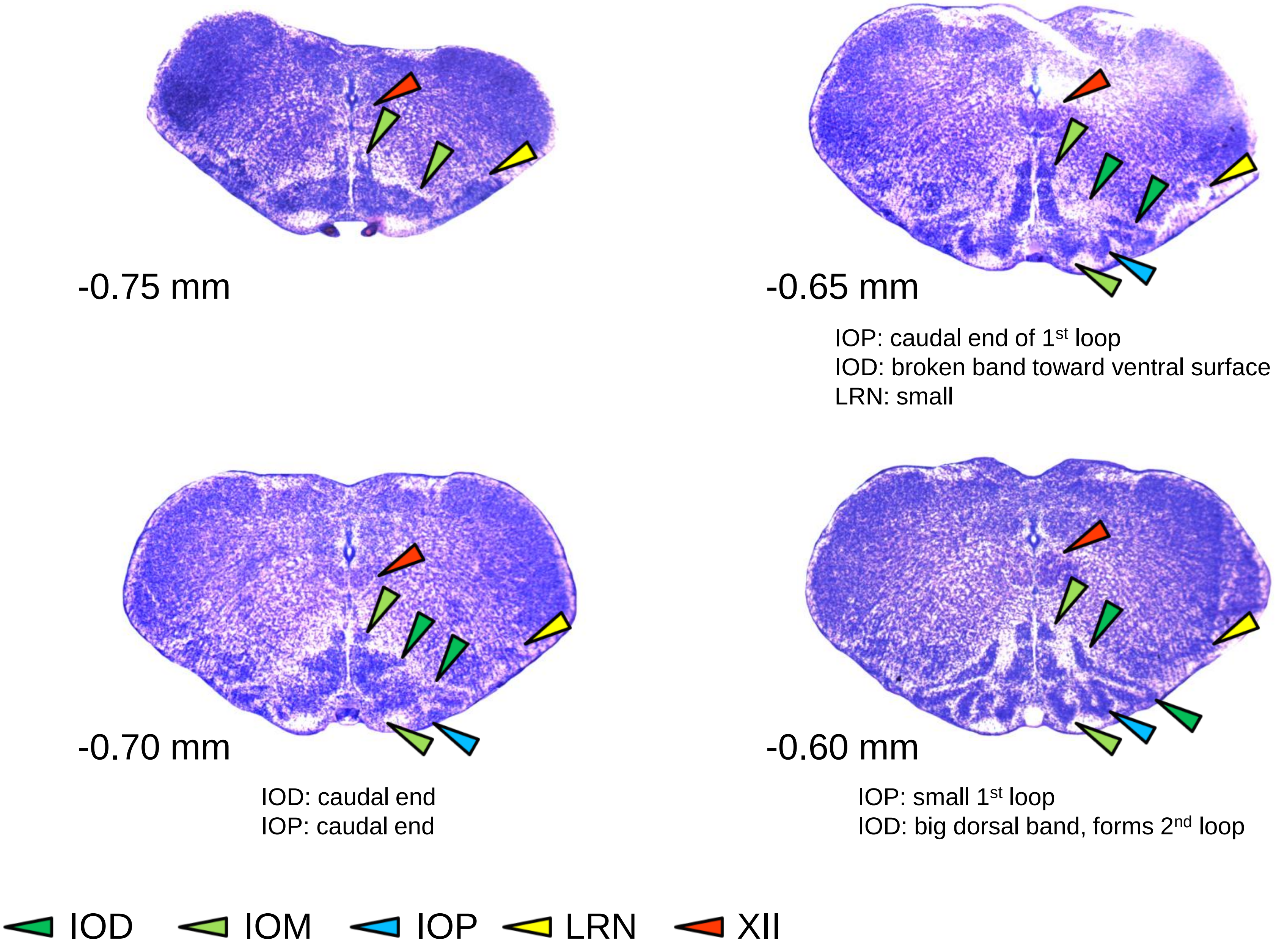

-0.75 mm
-0.65 mm
IOP: caudal end of 1st loop
IOD: broken band toward ventral surface
LRN: small
-0.70 mm
IOD: caudal end
IOP: caudal end
-0.60 mm
IOP: small 1st loop
IOD: big dorsal band, forms 2nd loop
IOD
IOM
IOP
LRN
XII

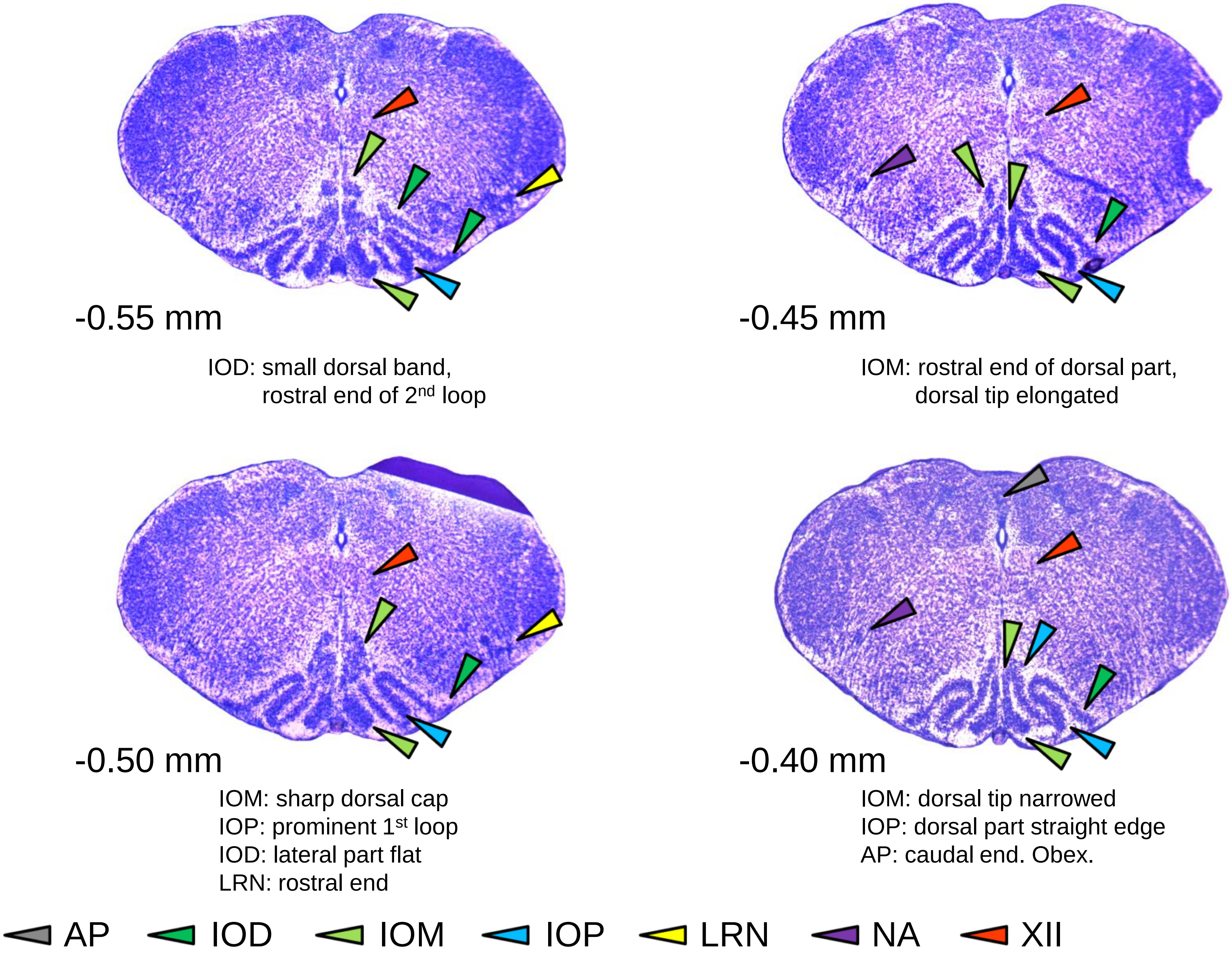
-0.55 mm
IOD: small dorsal band,
rostral end of 2nd loop
-0.45 mm
IOM: rostral end of dorsal part,
dorsal tip elongated
-0.50 mm
IOM: sharp dorsal cap
IOP: prominent 1st loop
IOD: lateral part flat
LRN: rostral end
-0.40 mm
IOM: dorsal tip narrowed
IOP: dorsal part straight edge
AP: caudal end. Obex.
AP
IOD
IOM
IOP
LRN
NA
XII

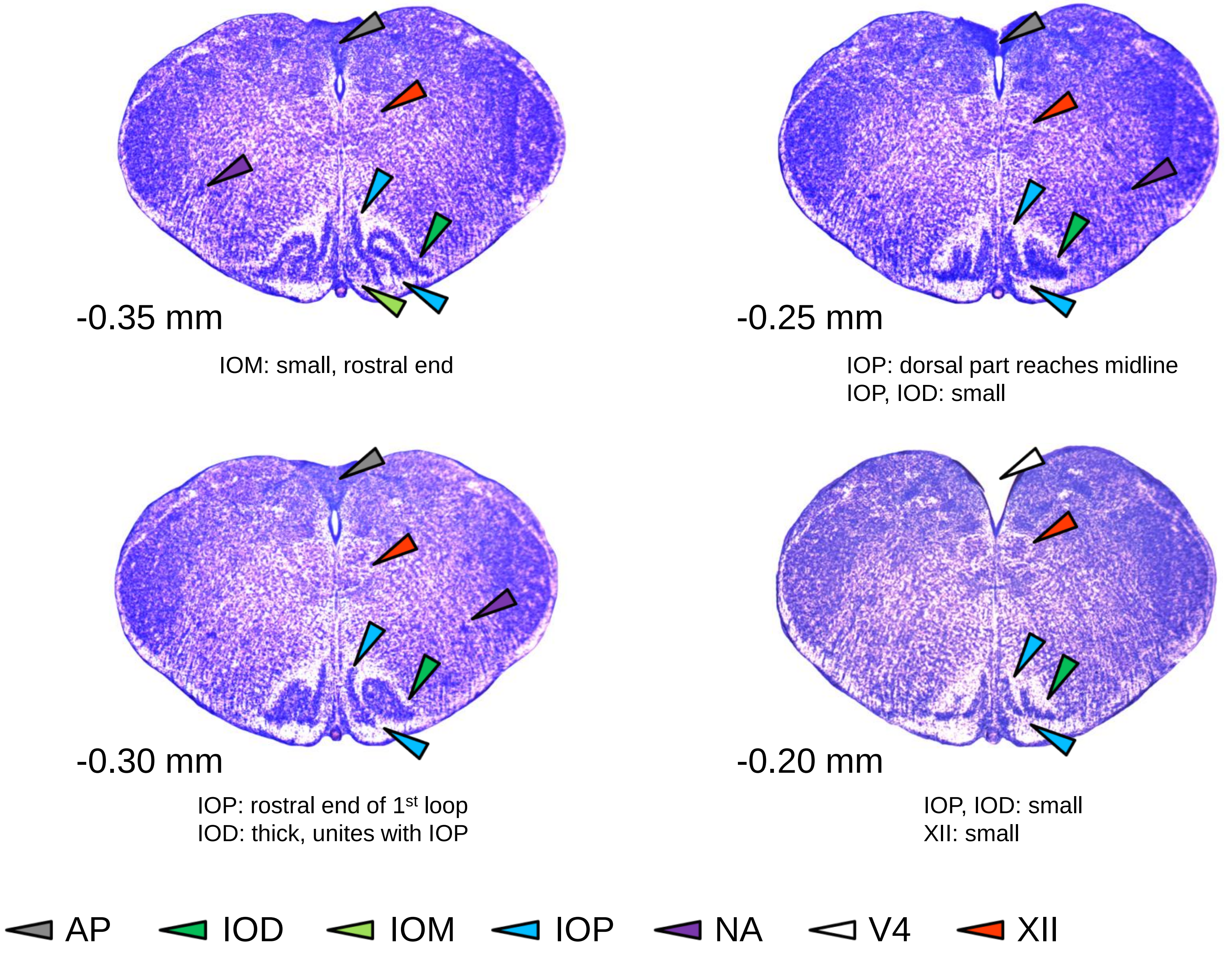

-0.35 mm
IOM: small, rostral end
-0.25 mm
IOP: dorsal part reaches midline
IOP, IOD: small
-0.30 mm
IOP: rostral end of 1st loop
IOD: thick, unites with IOP
-0.20 mm
IOP, IOD: small
XII: small
AP
IOD
IOM
IOP
NA
V4
XII

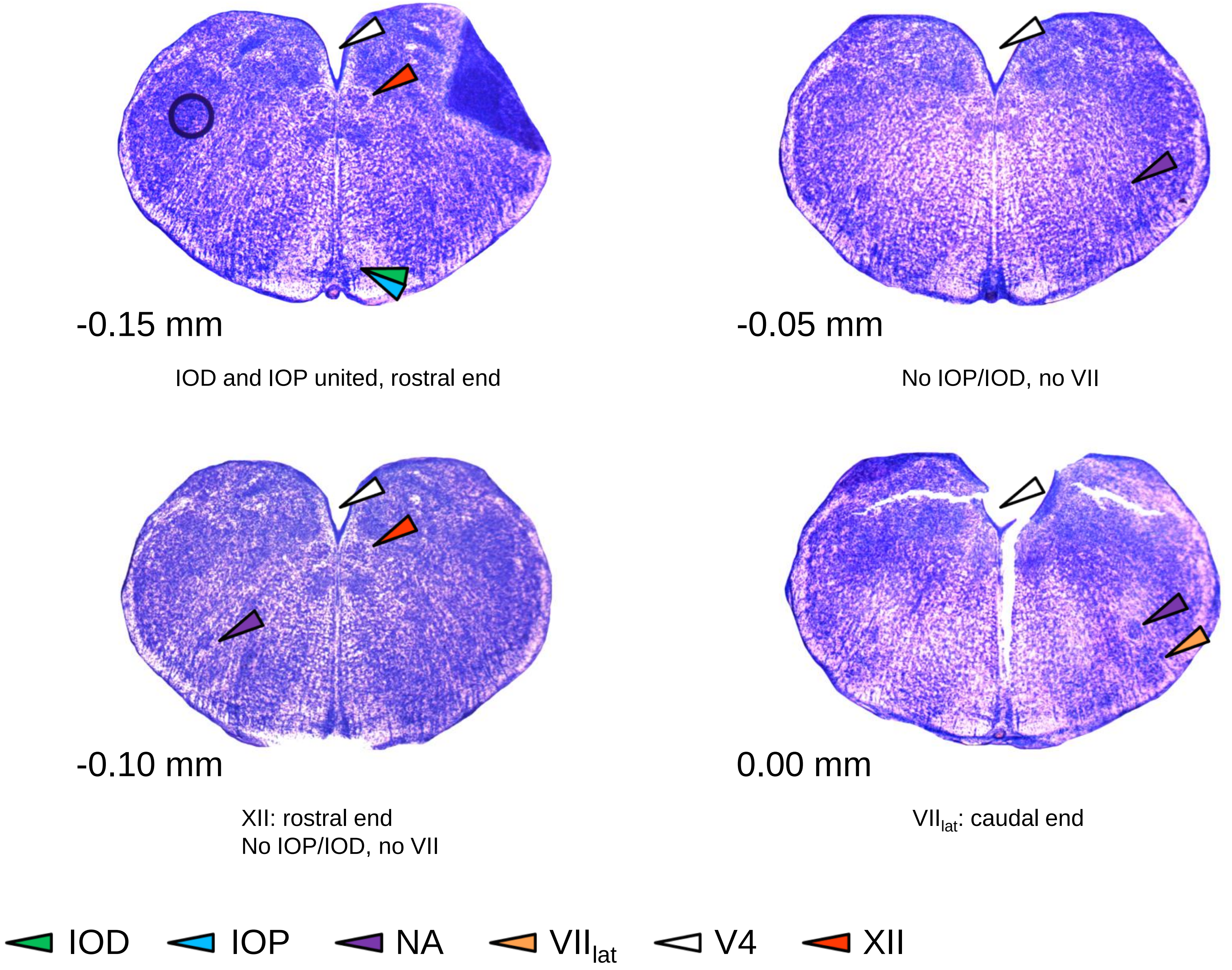

-0.15 mm
IOD and IOP united, rostral end
-0.05 mm
No IOP/IOD, no VII
-0.10 mm
XII: rostral end
No IOP/IOD, no VII
0.00 mm
VII_{lat}: caudal end
IOD
IOP
NA
VII_{lat}
V4
XII

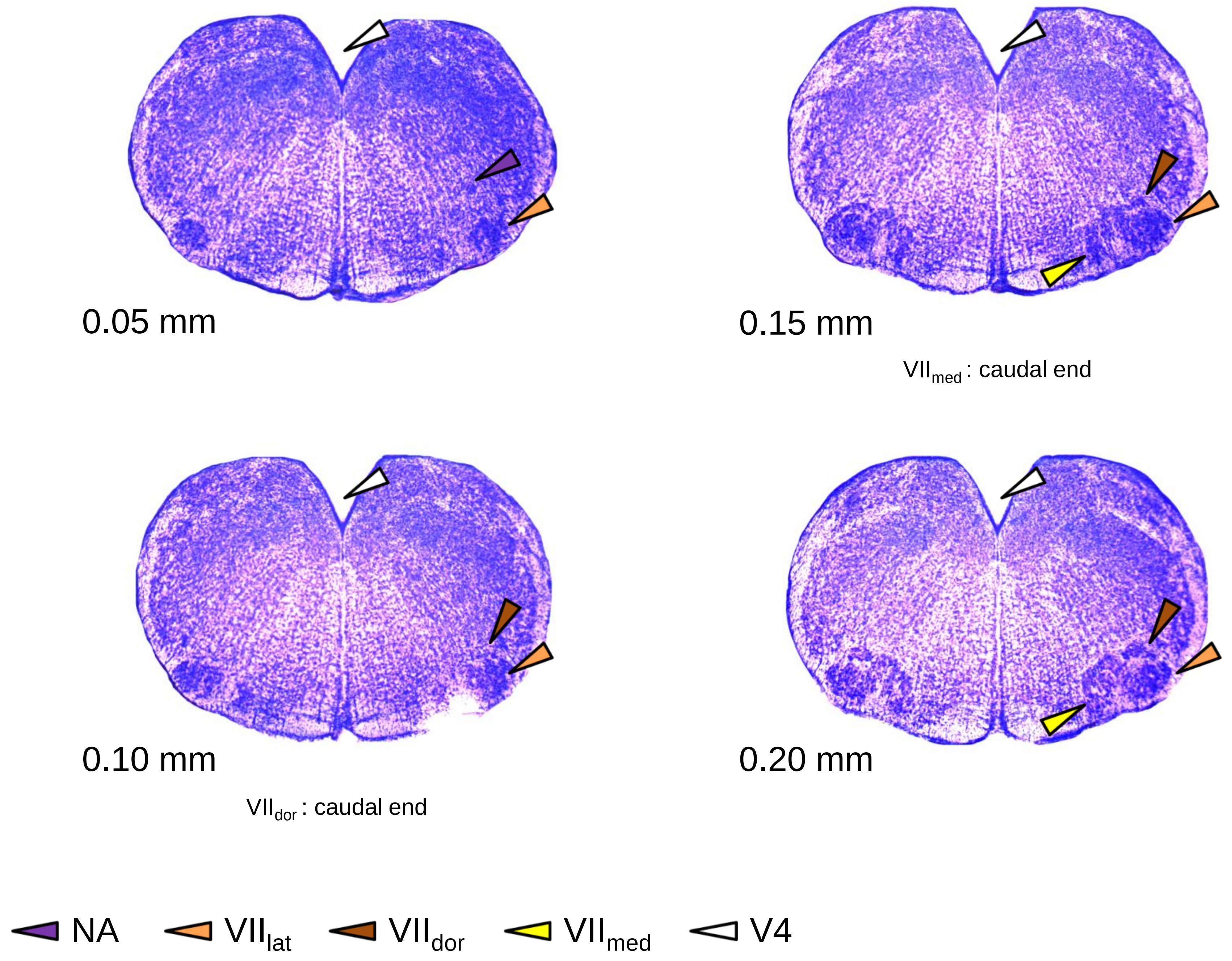
0.05 mm
0.15 mm
VII$_{med}$: caudal end
0.10 mm
VII$_{dor}$: caudal end
0.20 mm
NA
VII$_{lat}$
VII$_{dor}$
VII$_{med}$
V4

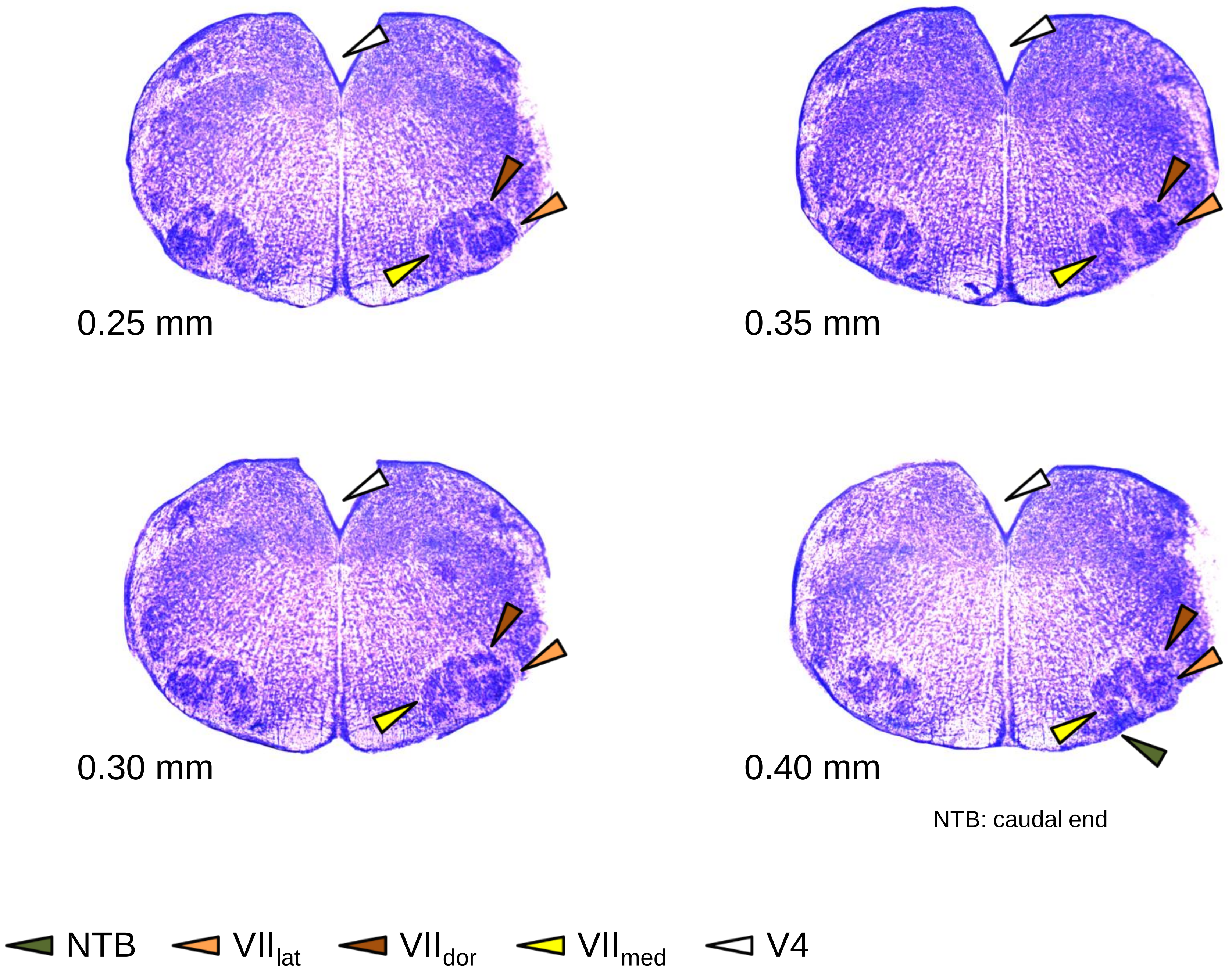
0.25 mm
0.35 mm
0.30 mm
0.40 mm
NTB: caudal end
NTB
VII_{lat}
VII_{dor}
VII_{med}
V4

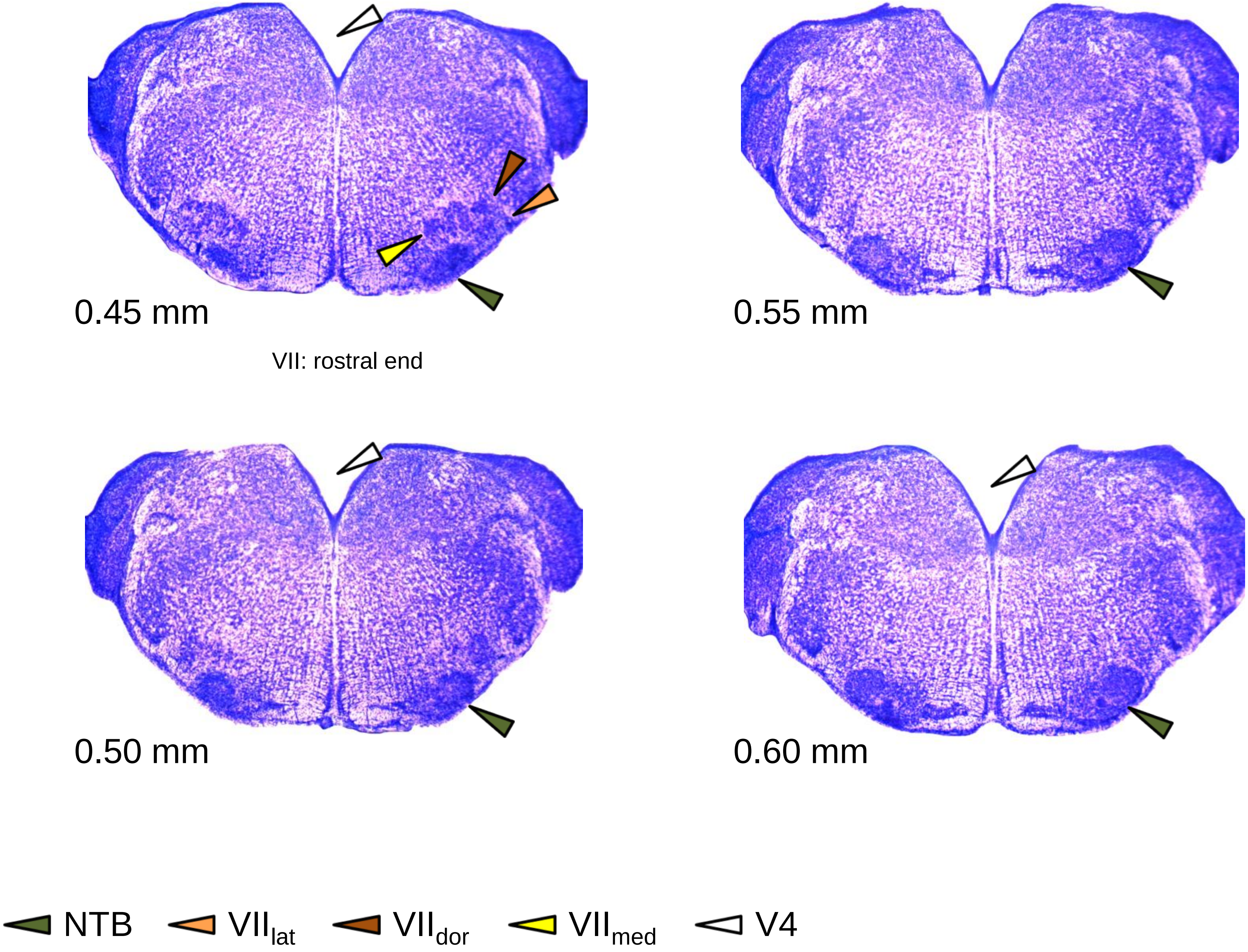
0.45 mm
VII: rostral end
0.55 mm
0.50 mm
0.60 mm
NTB
VII_{lat}
VII_{dor}
VII_{med}
V4
