# Supplementary material for: Identification of the pre‐Bötzinger complex inspiratory center in calibrated “sandwich” slices from newborn mice with fluorescent Dbx1 interneurons
Source: Physiol Rep. 2014 Aug 19;2(8):e12111. doi: 10.14814/phy2.12111 (PMC4246597; doi:10.14814/phy2.12111)
Supplement: Supplementary file 2 — Figure S2. Atlas of Dbx1 mouse (P4) medulla oblongata from a series of 50 μm thick thionin stained transverse sections. PDF [file phy2-2-e12111-s2.pdf]

Atlas of *Dbx1* mouse (P4) medulla oblongata

Ruangkittisakul A[1], Kottick A[2], Picardo MCD[2],
Ballanyi K[1]*, Del Negro CA[2]*

[1] Department of Physiology, University of Alberta,
Edmonton, AB, Canada

[2] Department of Applied Science, The College of William & Mary,
Williamsburg, VA, USA

Abbreviations

AP	area postrema
IO	inferior olive
IOD	dorsal inferior olive
IOM	medial inferior olive
IOP	principal inferior olive
LRN	lateral reticular nucleus
NA	nucleus ambiguus, compact formation
NTB	nucleus of the trapezoid body
PD	pyramidal decussation
OBEX	Obex, the point where the central canal opens to the 4th ventricle
V4	4th ventricle
VII	facial nucleus
VIIdor	dorsal nucleus of the facial nucleus
VIImed	medial nucleus of the facial nucleus
VIIlat	lateral nucleus of the facial nucleus
XII	hypoglossal nucleus

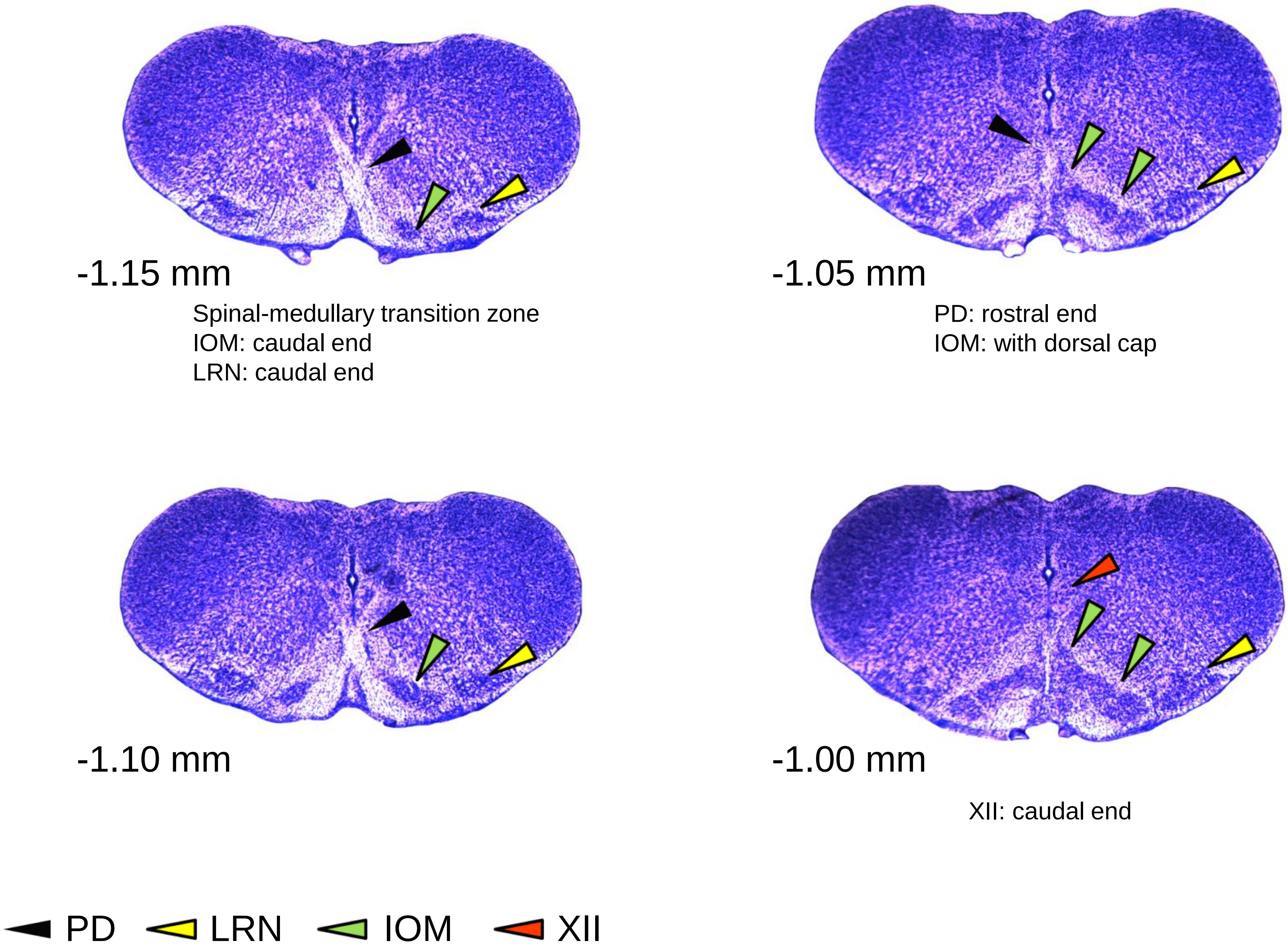
-1.15 mm
Spinal-medullary transition zone
IOM: caudal end
LRN: caudal end
-1.05 mm
PD: rostral end
IOM: with dorsal cap
-1.10 mm
-1.00 mm
XII: caudal end
PD
LRN
IOM
XII

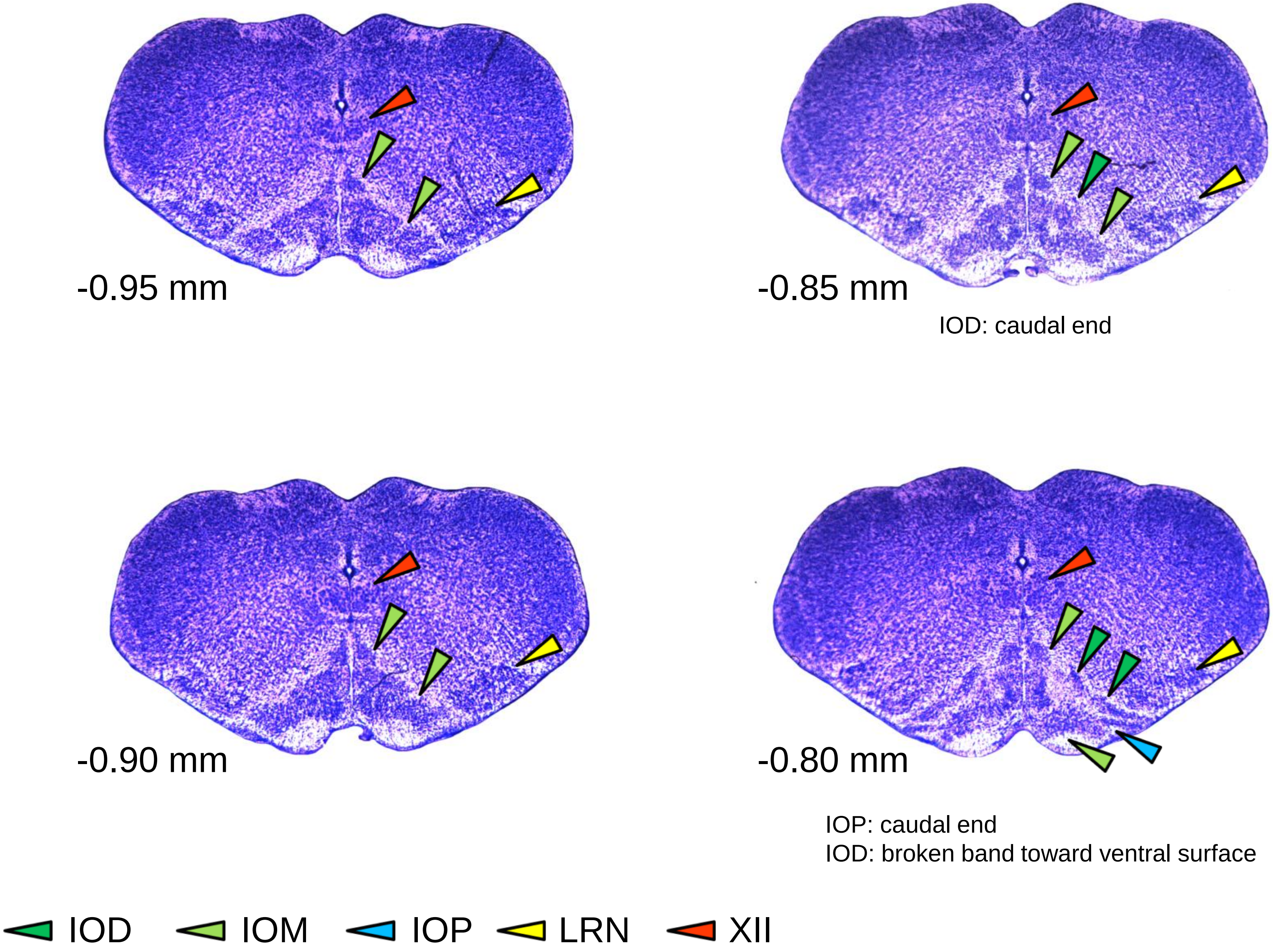
-0.95 mm
-0.85 mm
IOD: caudal end
-0.90 mm
-0.80 mm
IOP: caudal end
IOD: broken band toward ventral surface
IOD
IOM
IOP
LRN
XII

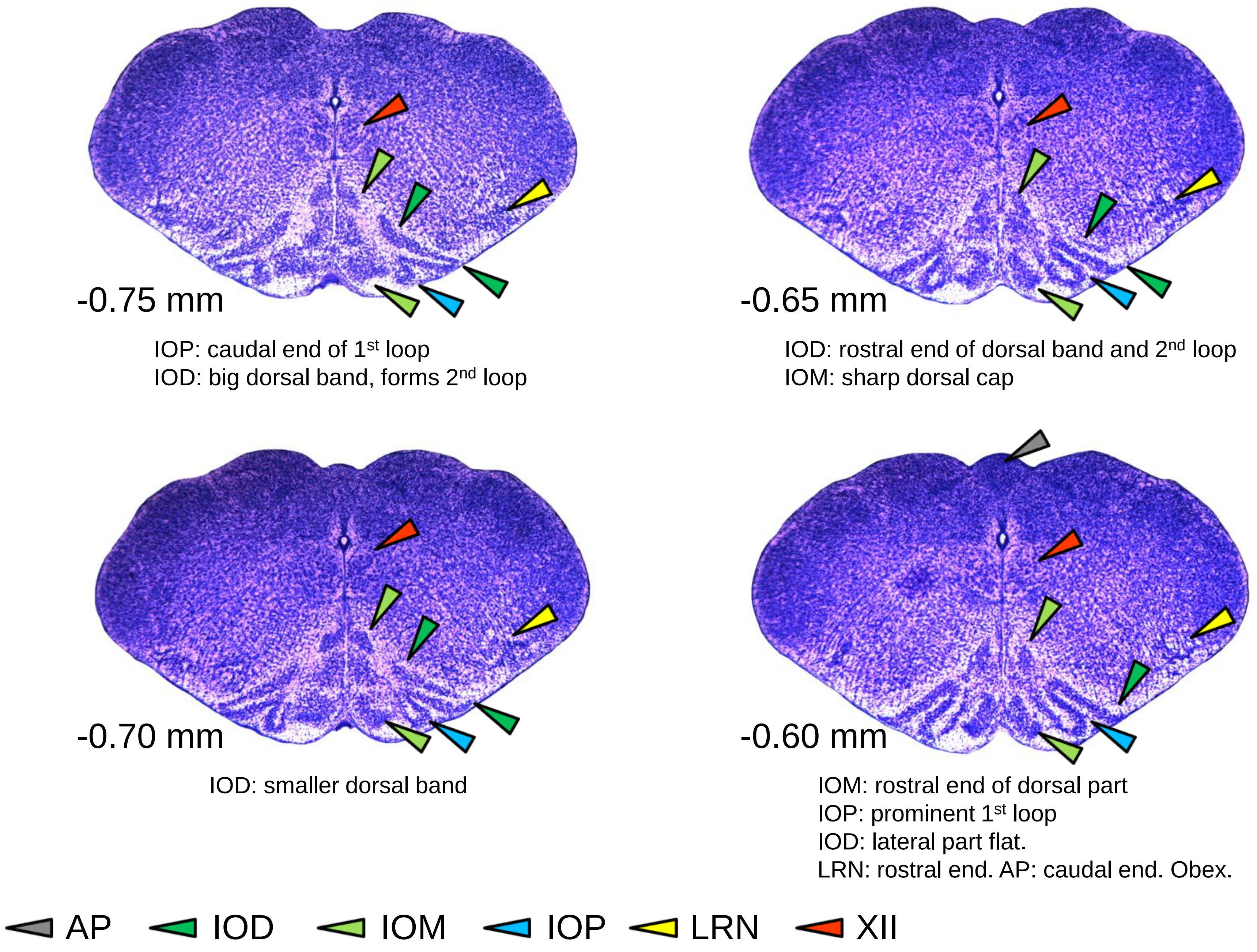

-0.75 mm
IOP: caudal end of 1st loop
IOD: big dorsal band, forms 2nd loop
-0.65 mm
IOD: rostral end of dorsal band and 2nd loop
IOM: sharp dorsal cap
-0.70 mm
IOD: smaller dorsal band
-0.60 mm
IOM: rostral end of dorsal part
IOP: prominent 1st loop
IOD: lateral part flat.
LRN: rostral end. AP: caudal end. Obex.
AP
IOD
IOM
IOP
LRN
XII

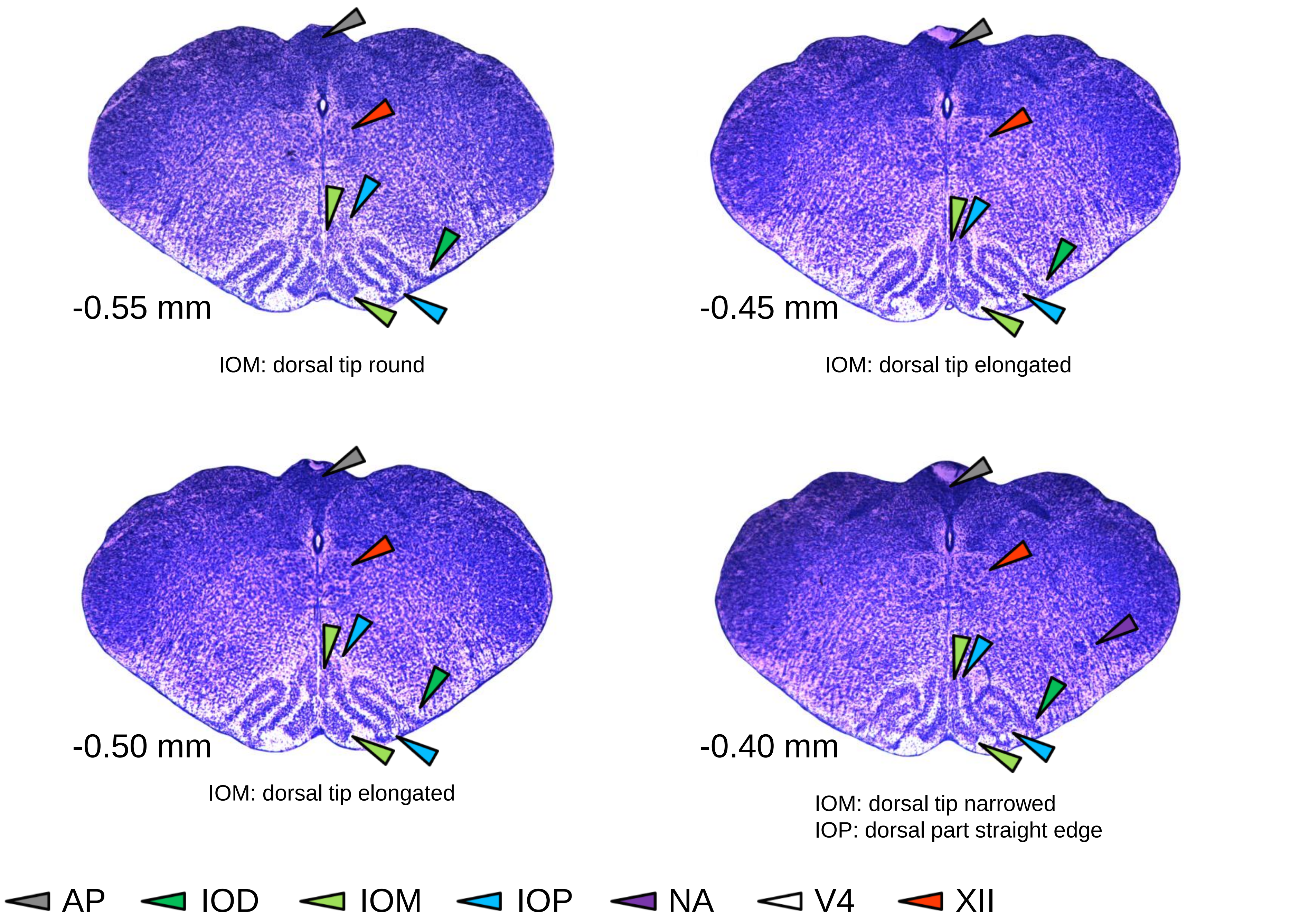
-0.55 mm
IOM: dorsal tip round
-0.45 mm
IOM: dorsal tip elongated
-0.50 mm
IOM: dorsal tip elongated
-0.40 mm
IOM: dorsal tip narrowed
IOP: dorsal part straight edge
AP
IOD
IOM
IOP
NA
V4
XII

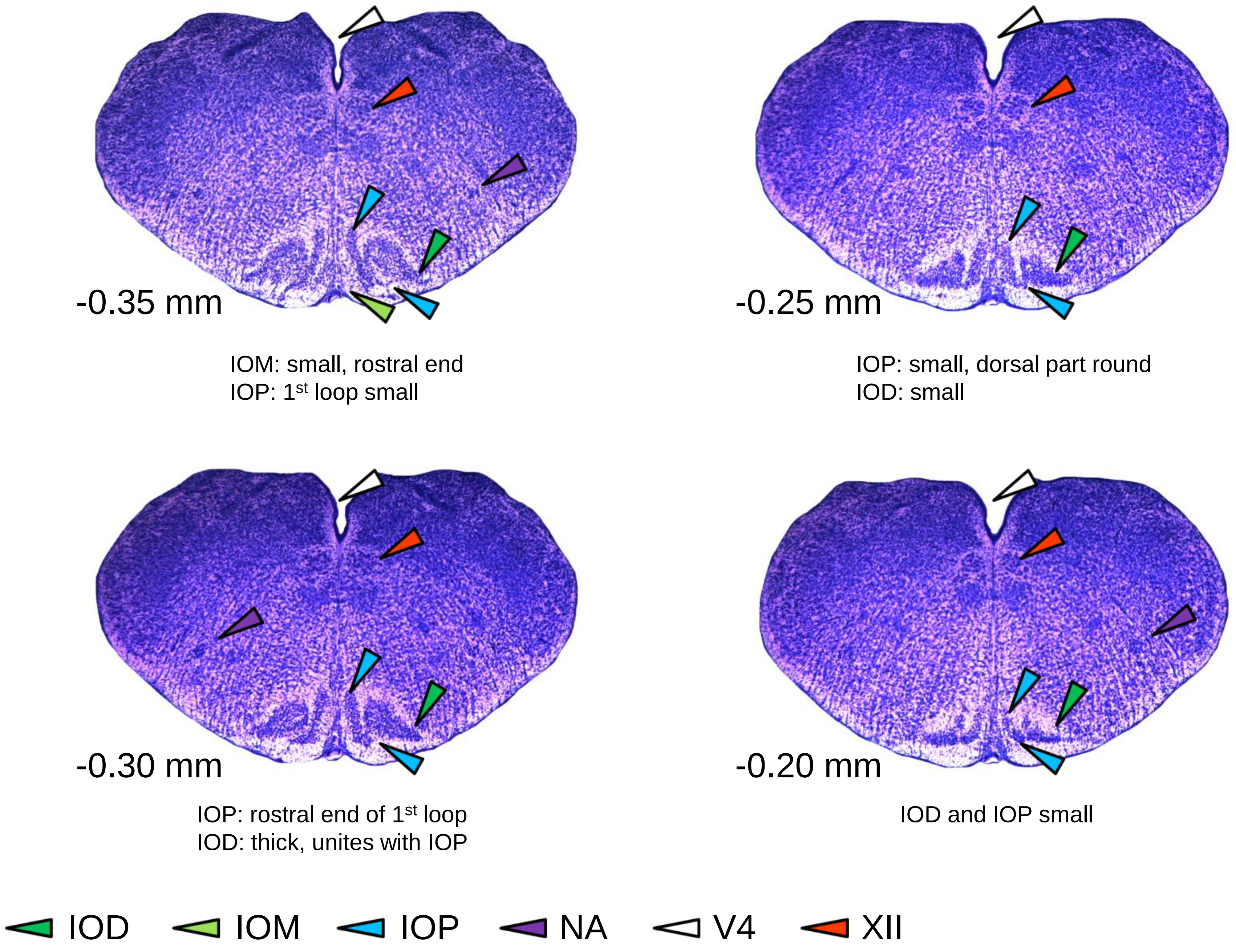
-0.35 mm
IOM: small, rostral end
IOP: 1st loop small
-0.25 mm
IOP: small, dorsal part round
IOD: small
-0.30 mm
IOP: rostral end of 1st loop
IOD: thick, unites with IOP
-0.20 mm
IOD and IOP small
IOD
IOM
IOP
NA
V4
XII

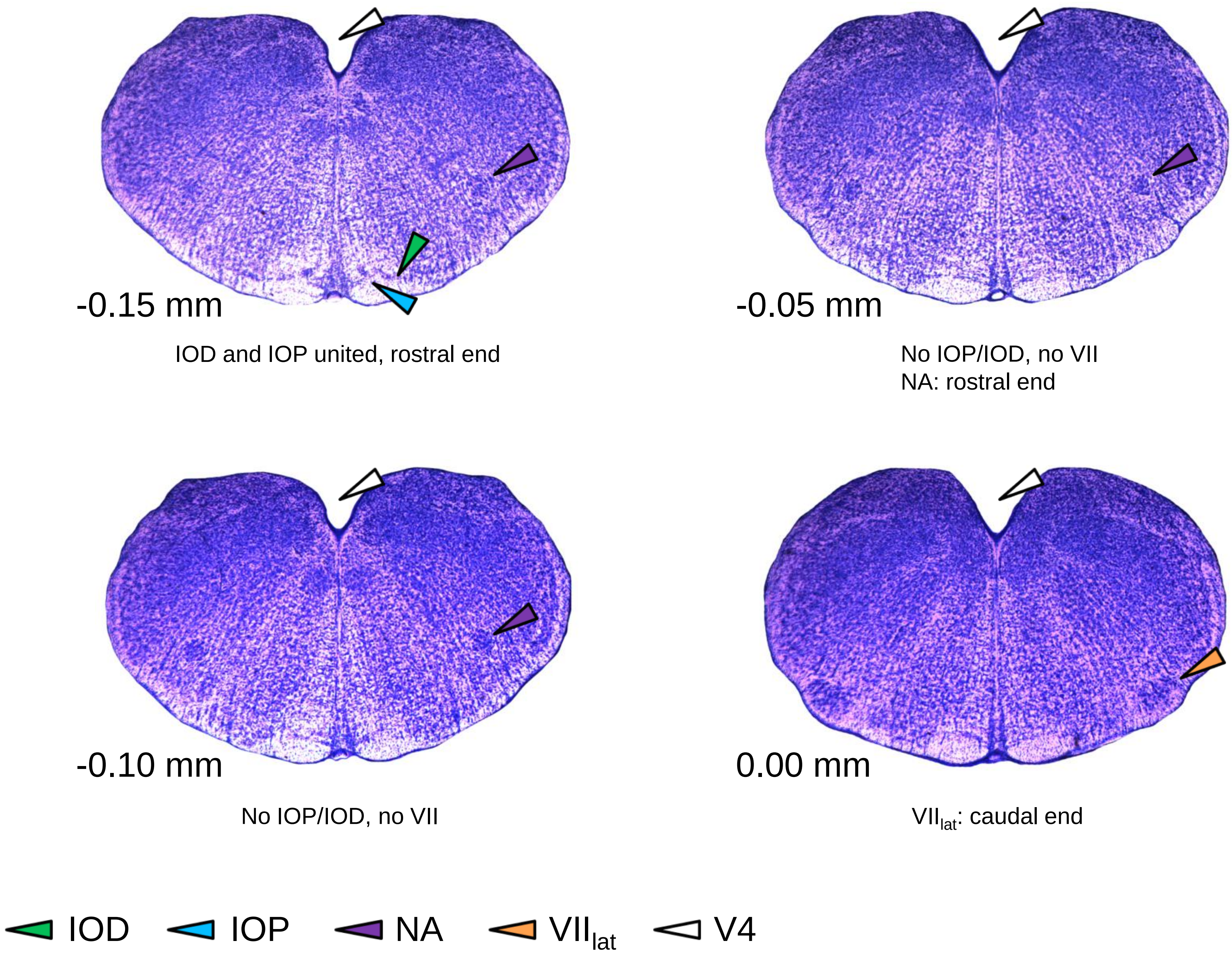
-0.15 mm
IOD and IOP united, rostral end
-0.05 mm
No IOP/IOD, no VII
NA: rostral end
-0.10 mm
No IOP/IOD, no VII
0.00 mm
VII$_{lat}$: caudal end
IOD
IOP
NA
VII$_{lat}$
V4

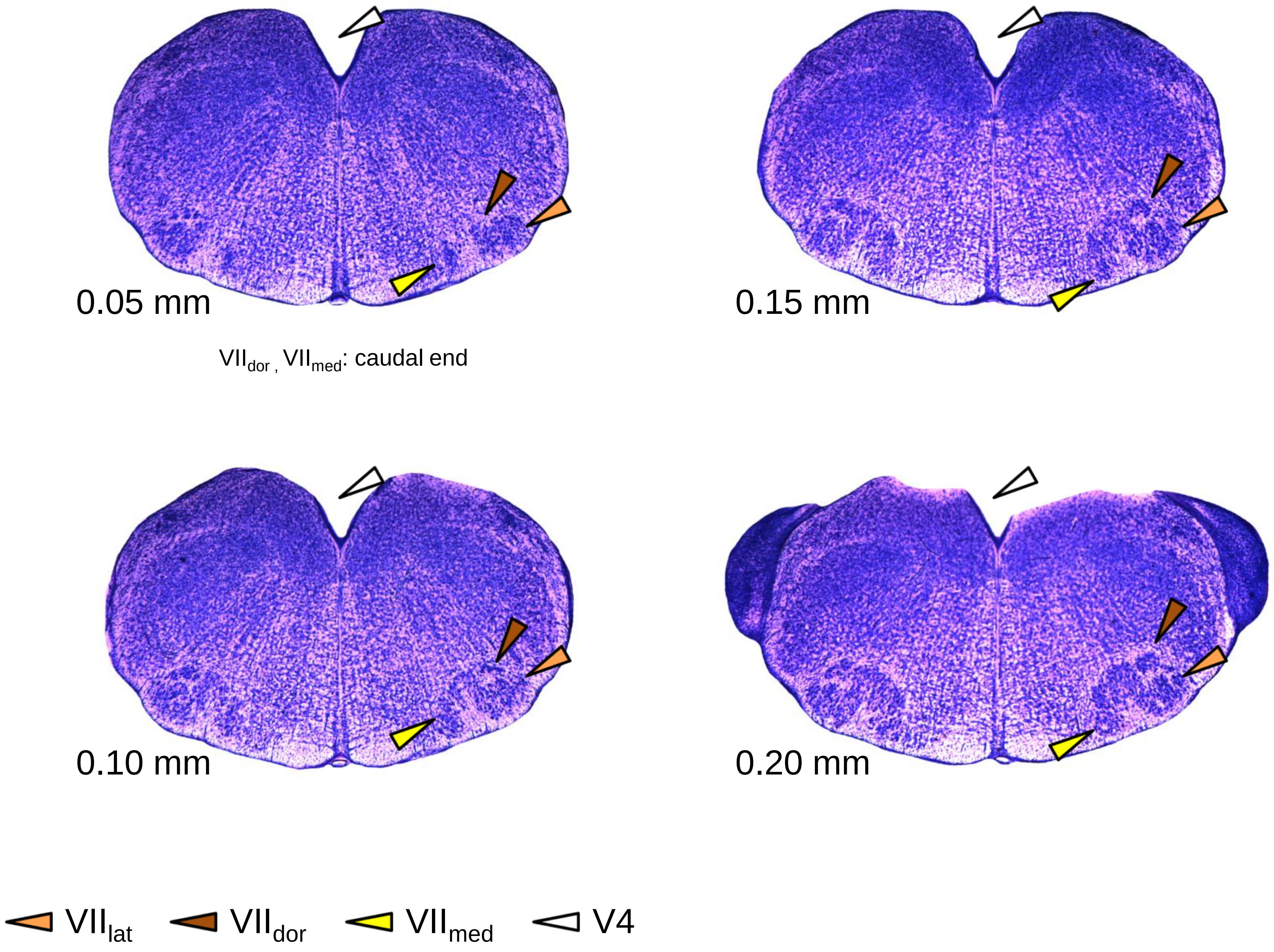

0.05 mm
0.15 mm
VIIdor , VIImed: caudal end
0.10 mm
0.20 mm
VIIlat
VIIdor
VIImed
V4

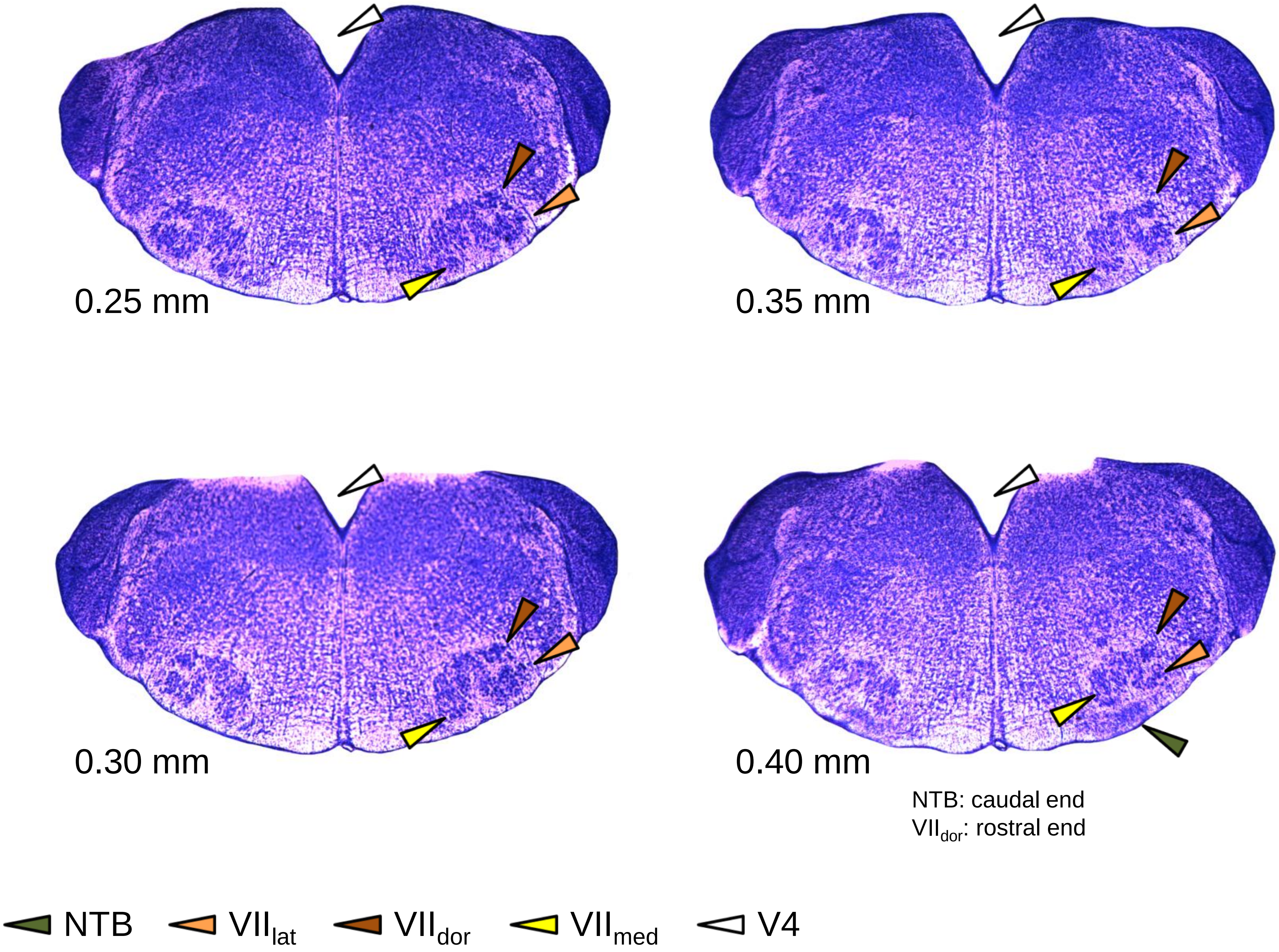

0.25 mm
0.35 mm
0.30 mm
0.40 mm
NTB: caudal end
VII_{dor}: rostral end
NTB
VII_{lat}
VII_{dor}
VII_{med}
V4

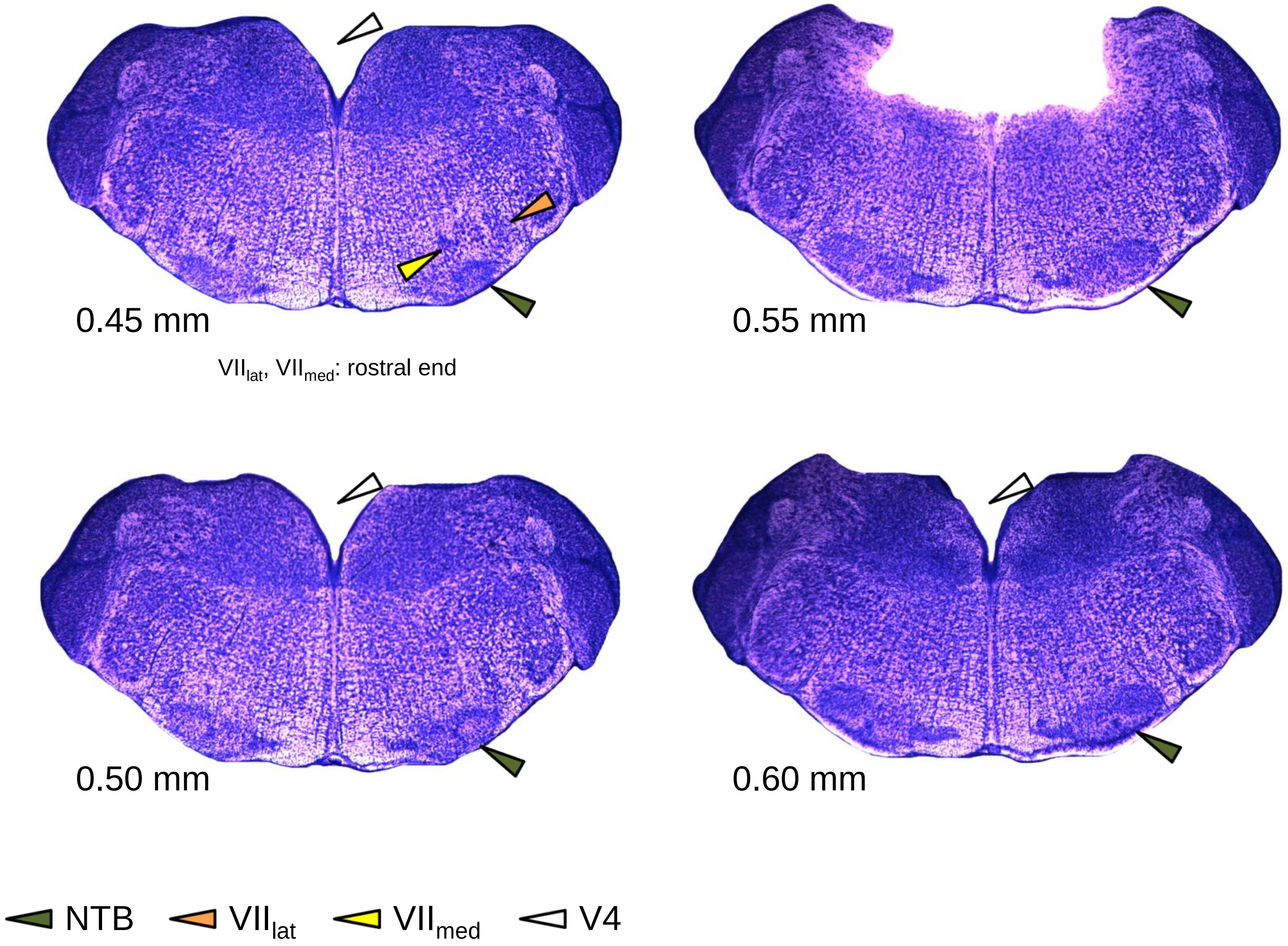
0.45 mm
VIIlat, VIImed: rostral end
0.55 mm
0.50 mm
0.60 mm
NTB
VIIlat
VIImed
V4
