# Supplementary material for: Identification of the pre‐Bötzinger complex inspiratory center in calibrated “sandwich” slices from newborn mice with fluorescent Dbx1 interneurons
Source: Physiol Rep. 2014 Aug 19;2(8):e12111. doi: 10.14814/phy2.12111 (PMC4246597; doi:10.14814/phy2.12111)
Supplement: Supplementary file 3 — Figure S3. Atlas of tdTomato fluorescence in Dbx1 mouse (P4) medulla oblongata shown as fluorescence image (right) and overlay with bright field image (left) from 50 μm thick transverse sections. PDF [file phy2-2-e12111-s3.pdf]

Atlas of tdTomato Fluorescence in *Dbx1* mouse (P4) medulla oblongata

Ruangkittisakul A[1], Kottick A[2], Picardo MCD[2], Ballanyi K[1]*, Del Negro CA[2]*

[1] Department of Physiology, University of Alberta, Edmonton, AB, Canada

[2] Department of Applied Science, The College of William & Mary, Williamsburg, VA, USA

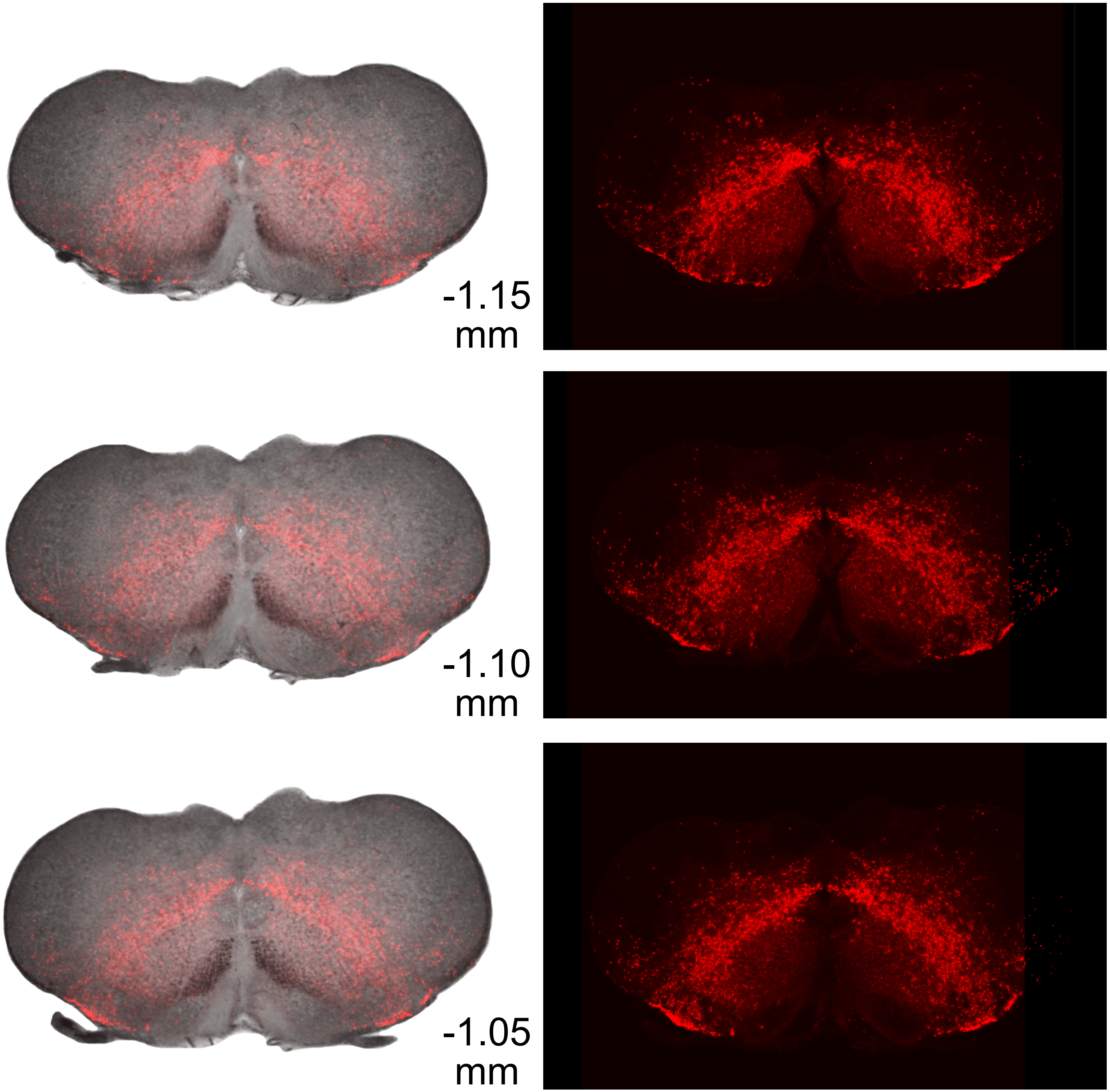
-1.15 mm
-1.10 mm
-1.05 mm

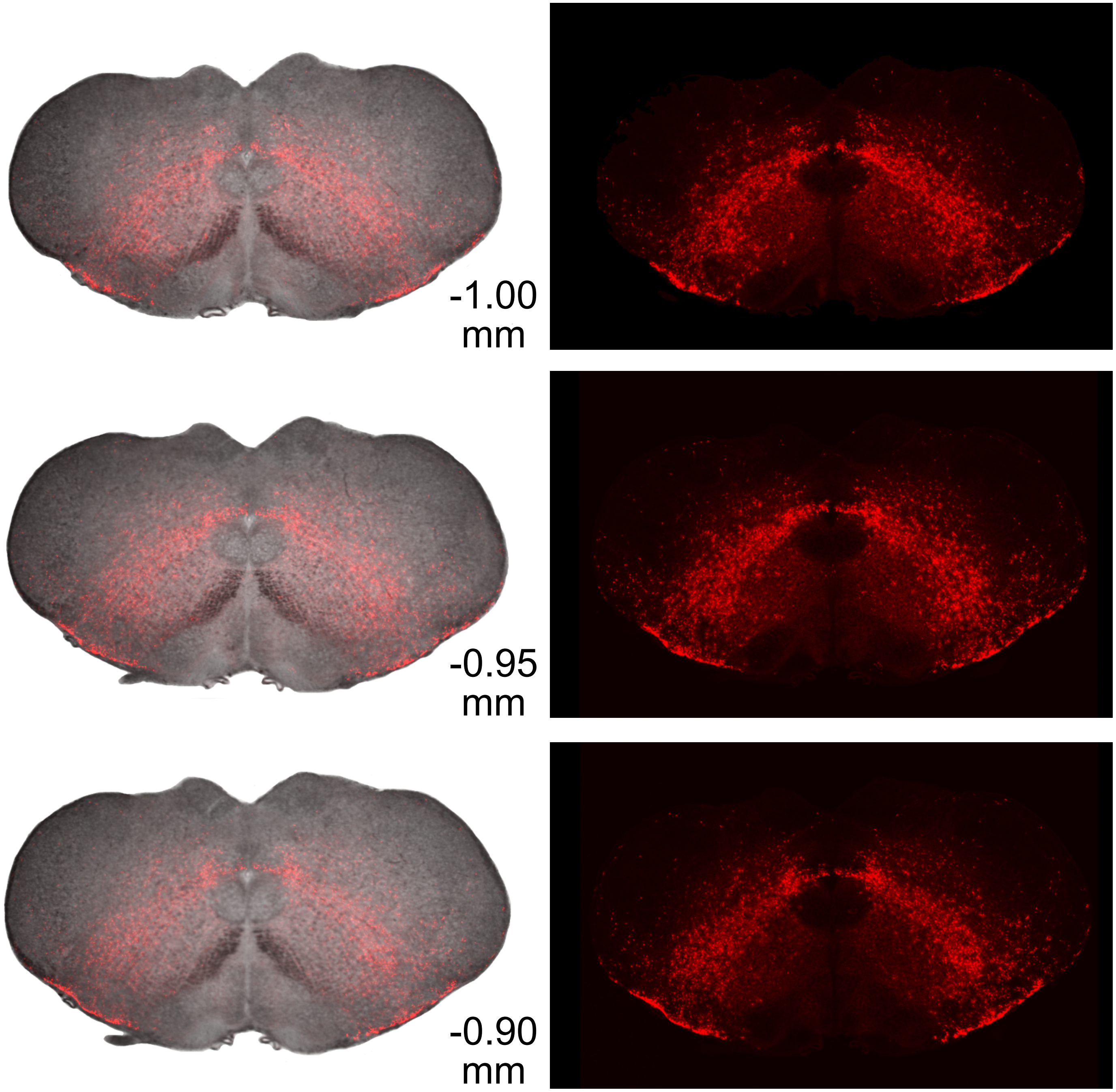
-1.00 mm
-0.95 mm
-0.90 mm

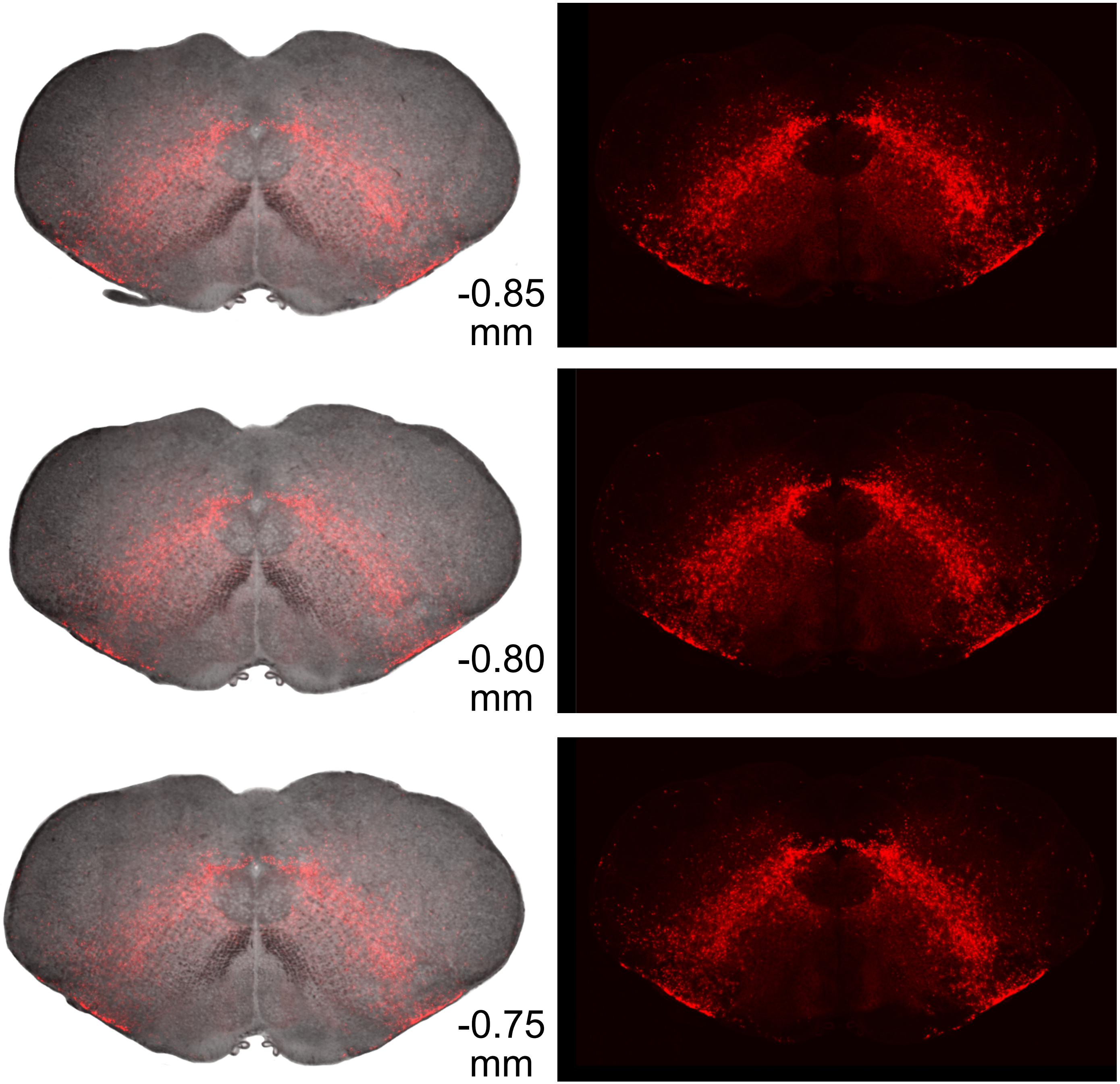
-0.85 mm
-0.80 mm
-0.75 mm

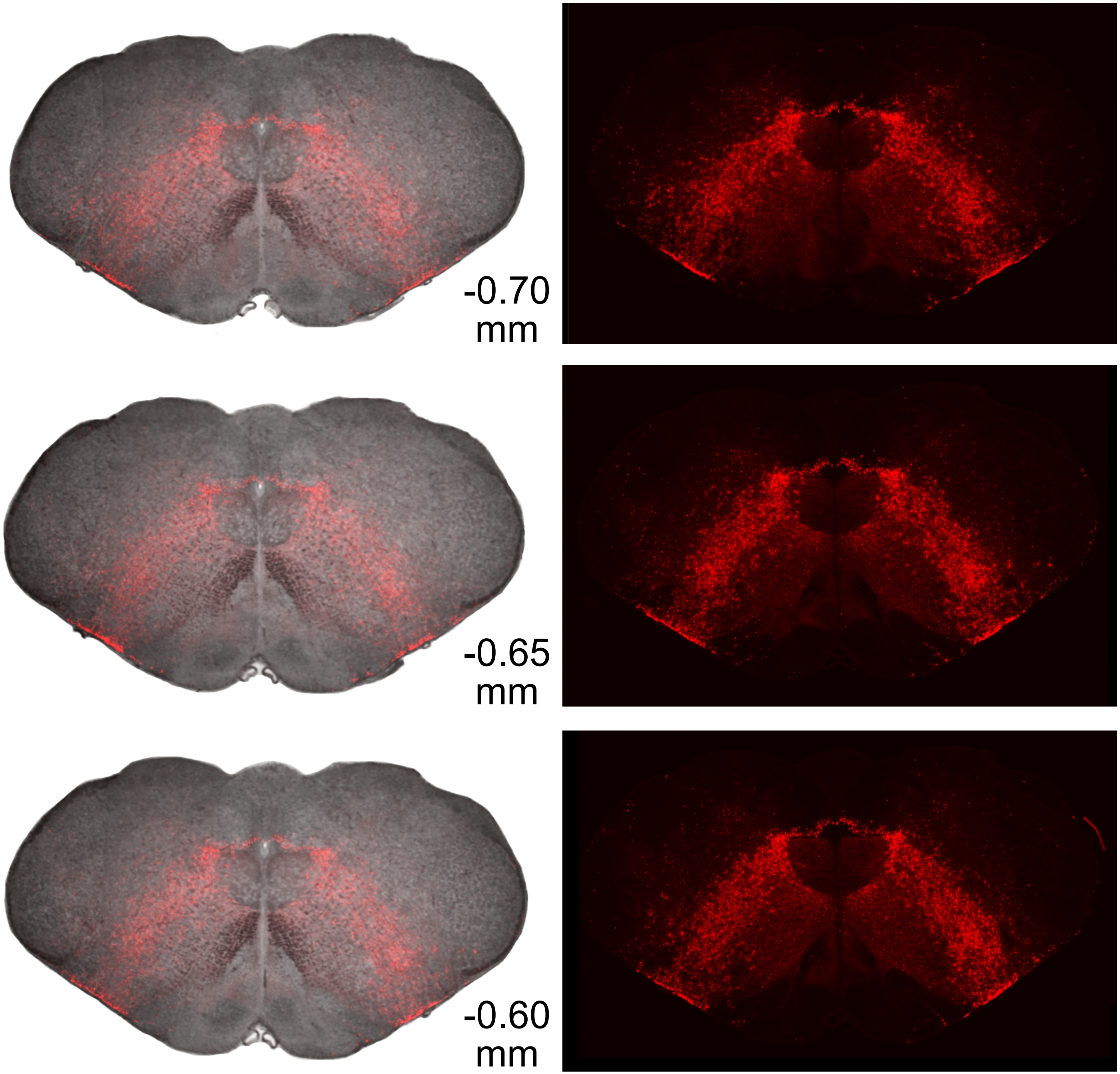
-0.70 mm
-0.65 mm
-0.60 mm

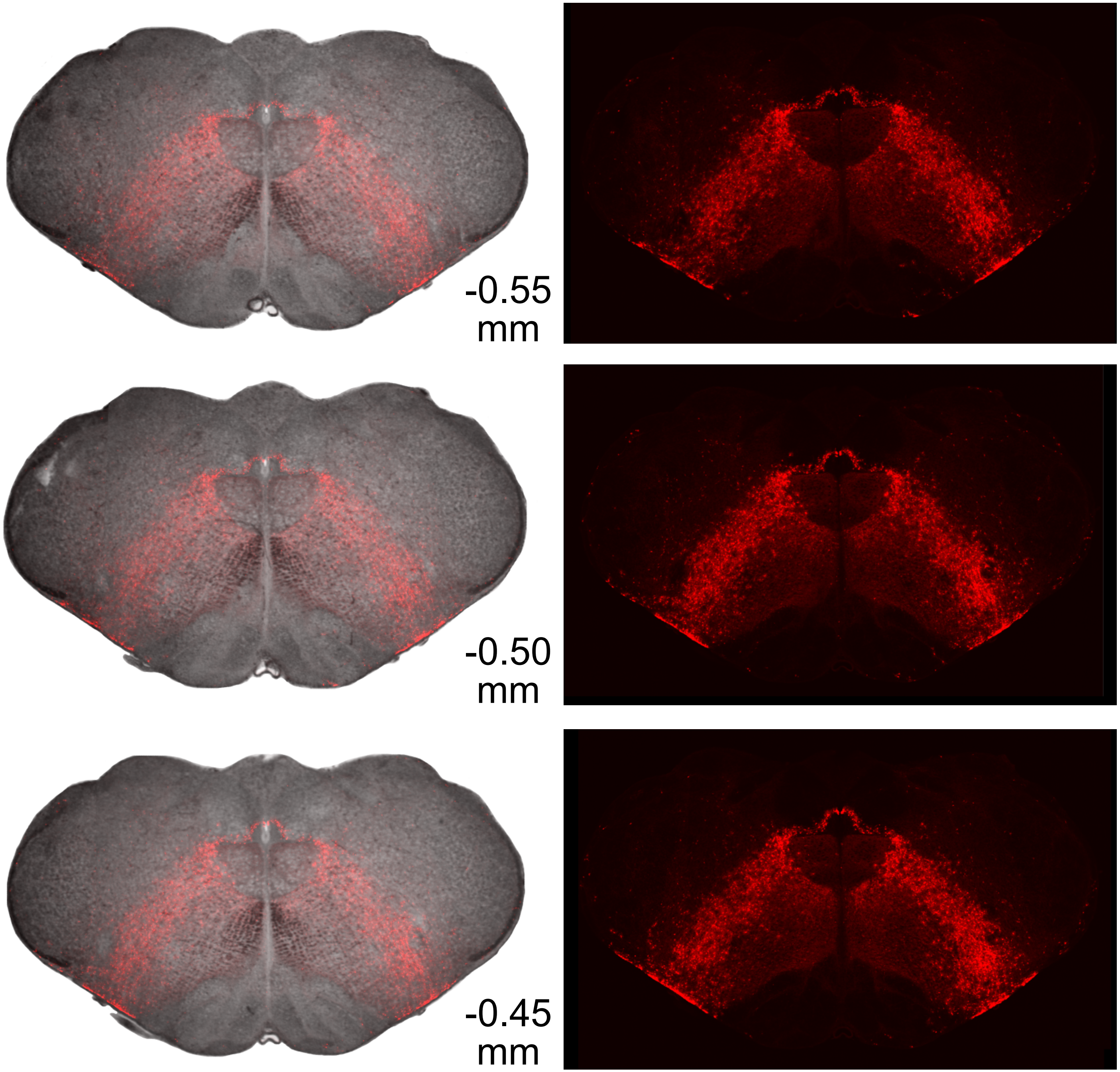

-0.55
mm
-0.50
mm
-0.45
mm

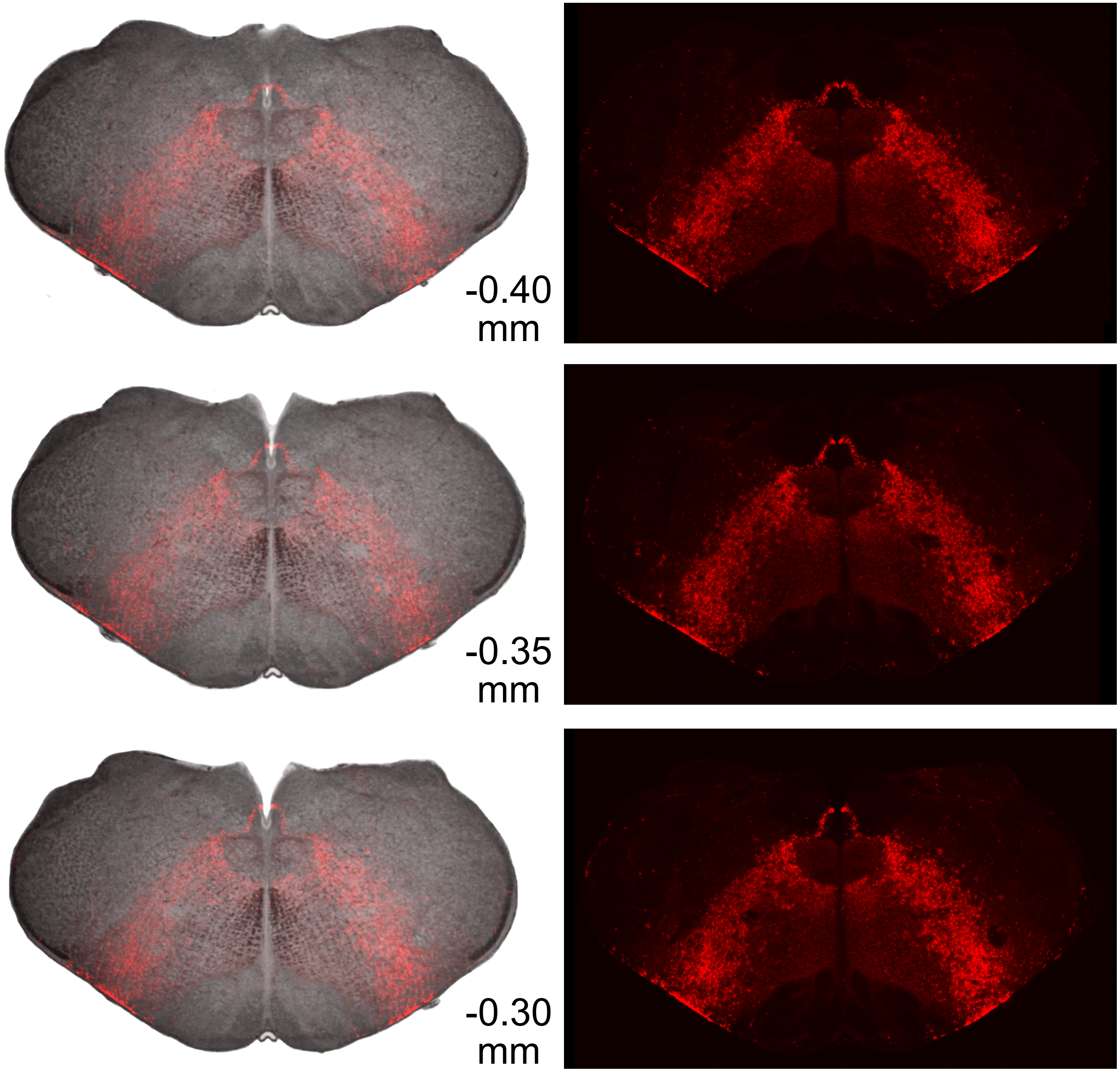

-0.40 mm
-0.35 mm
-0.30 mm

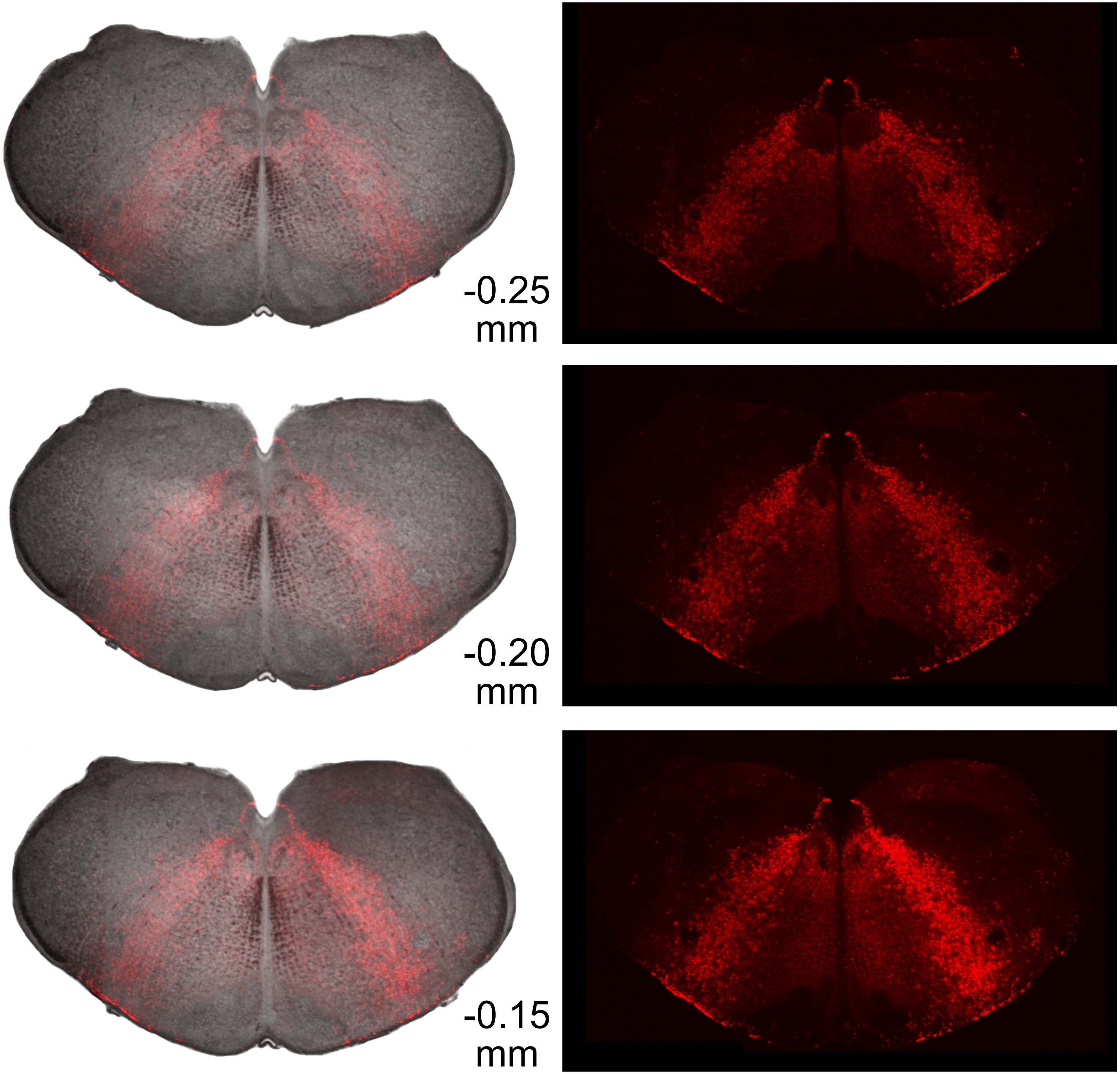
-0.25 mm
-0.20 mm
-0.15 mm

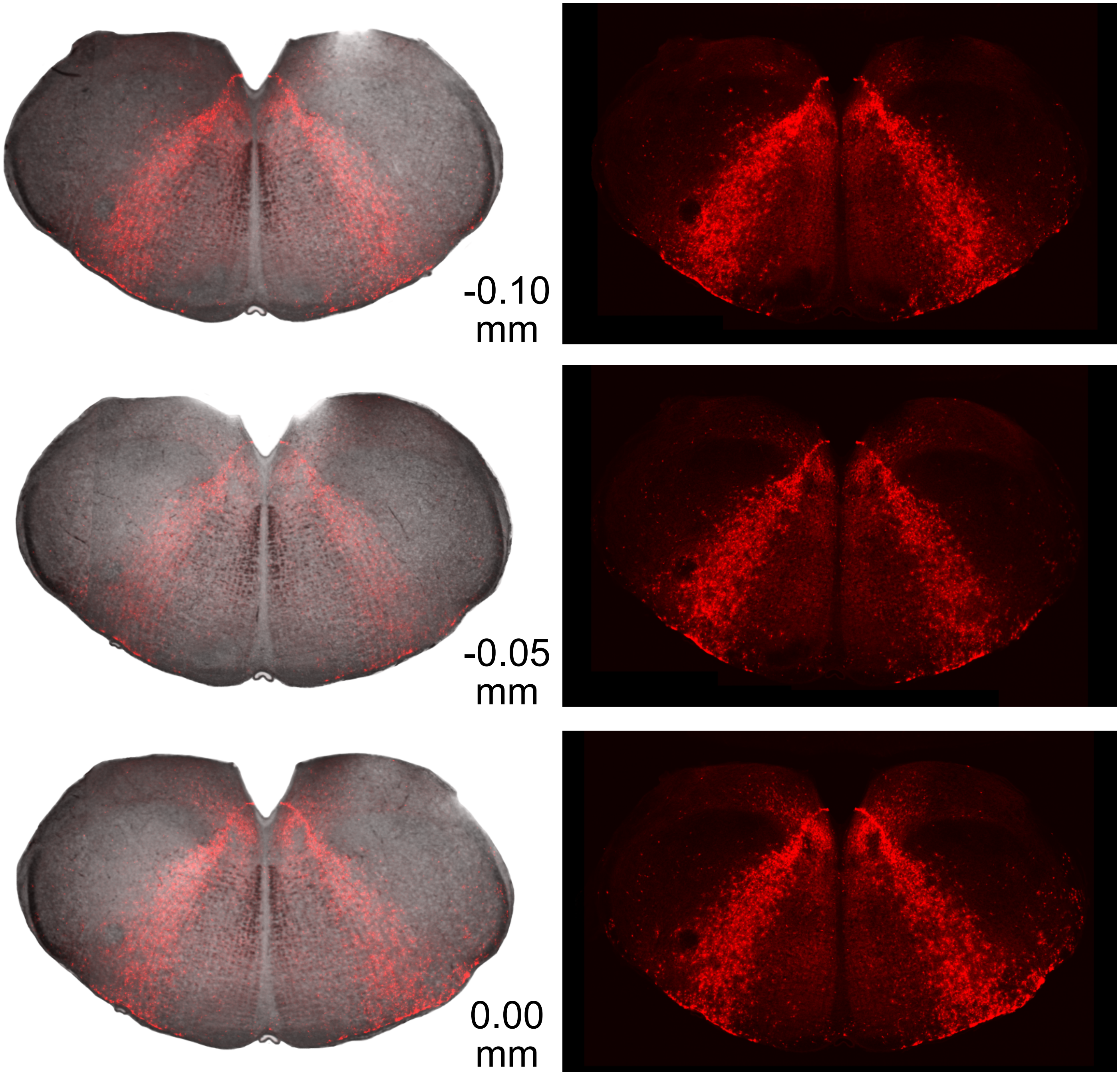
-0.10 mm
-0.05 mm
0.00 mm

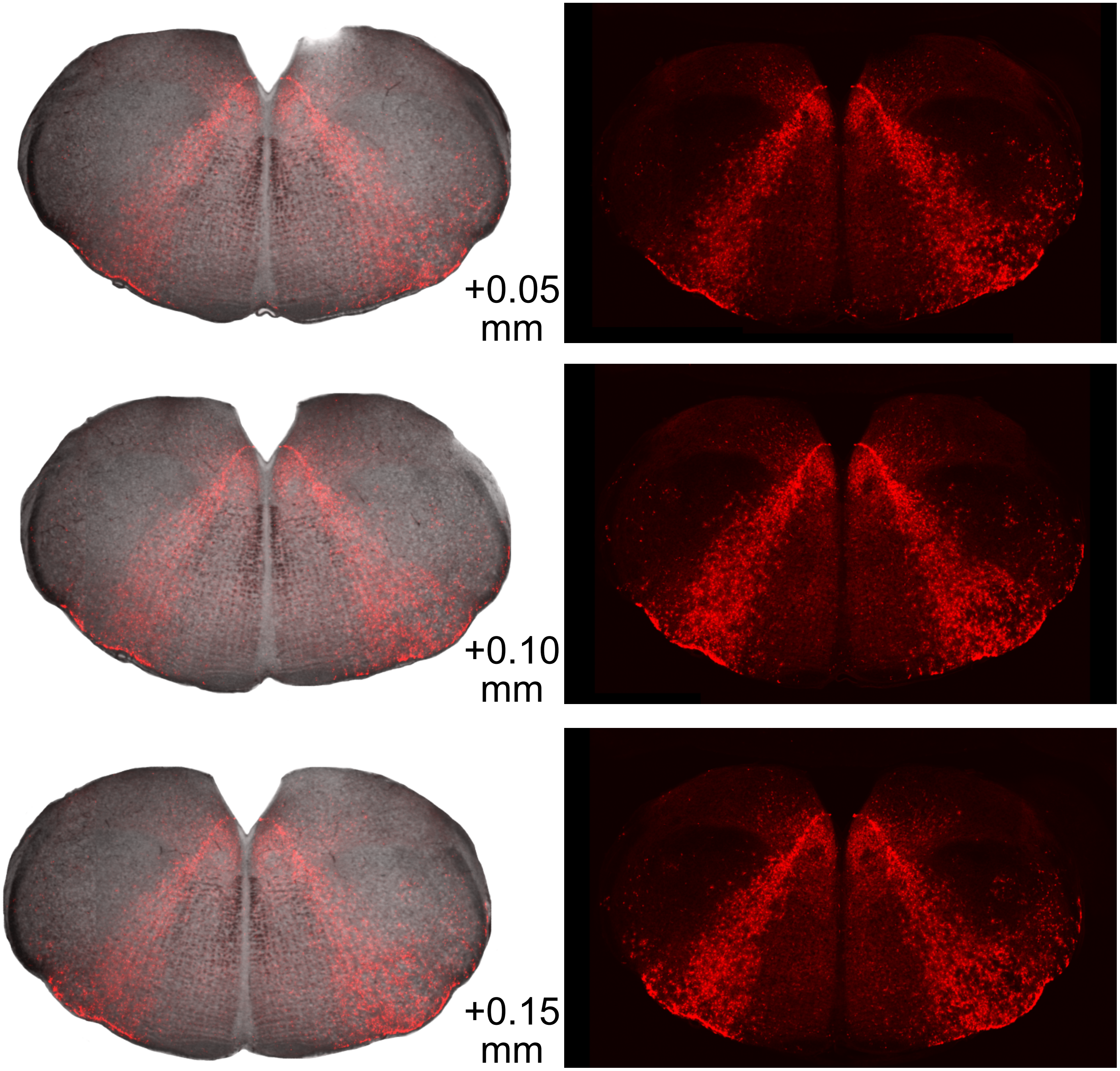
+0.05 mm
+0.10 mm
+0.15 mm

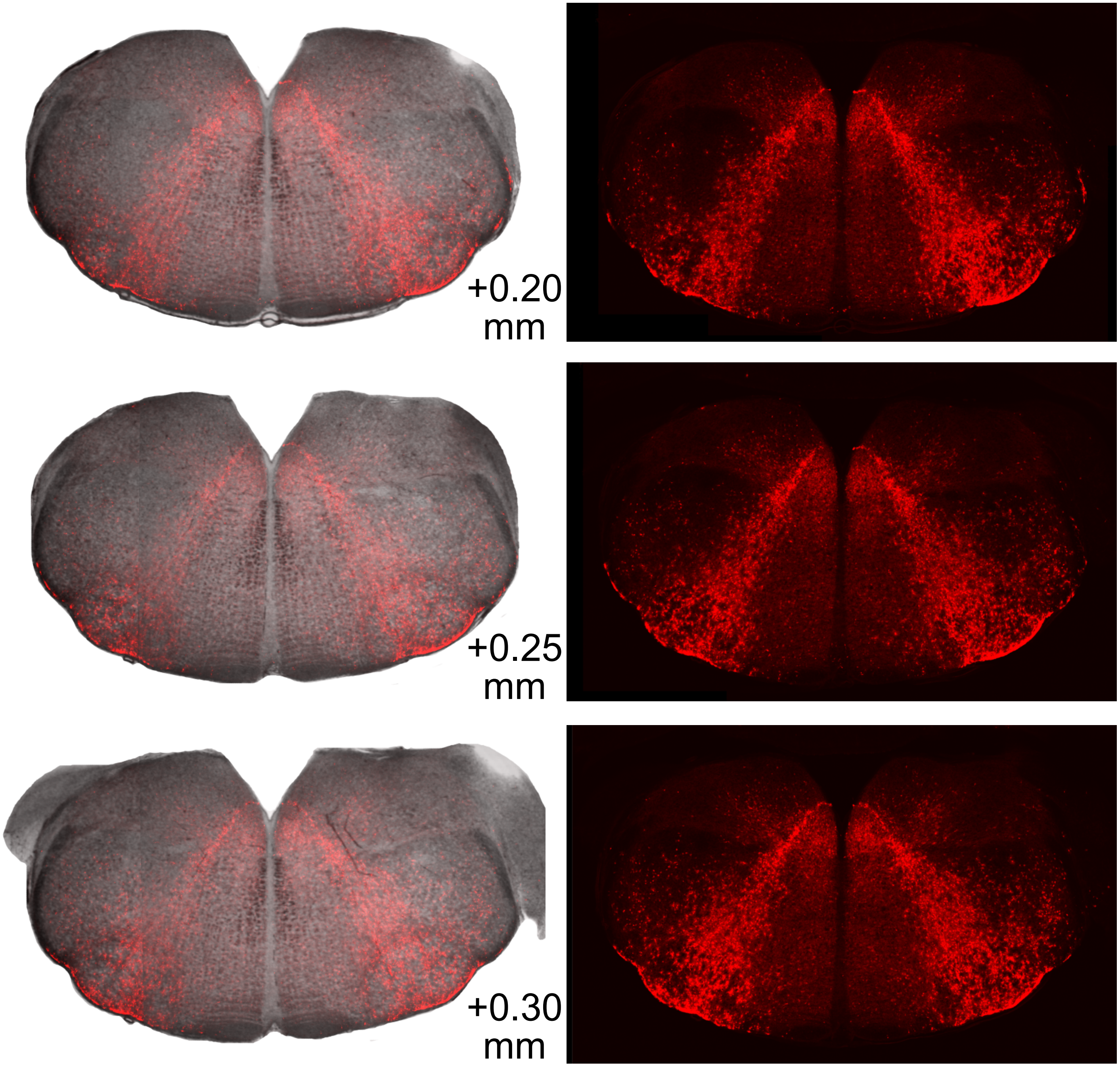
+0.20 mm
+0.25 mm
+0.30 mm

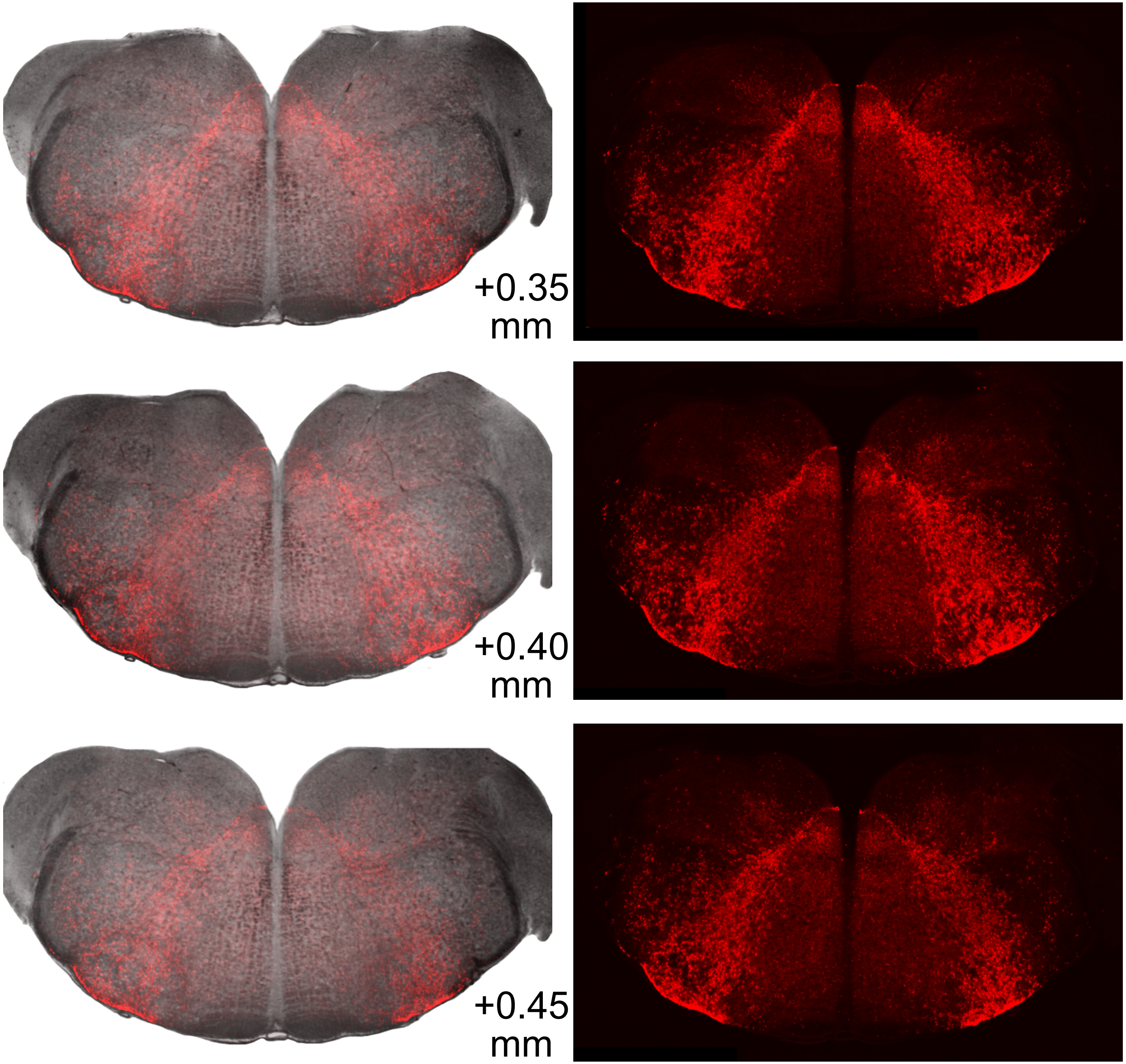
+0.35 mm
+0.40 mm
+0.45 mm

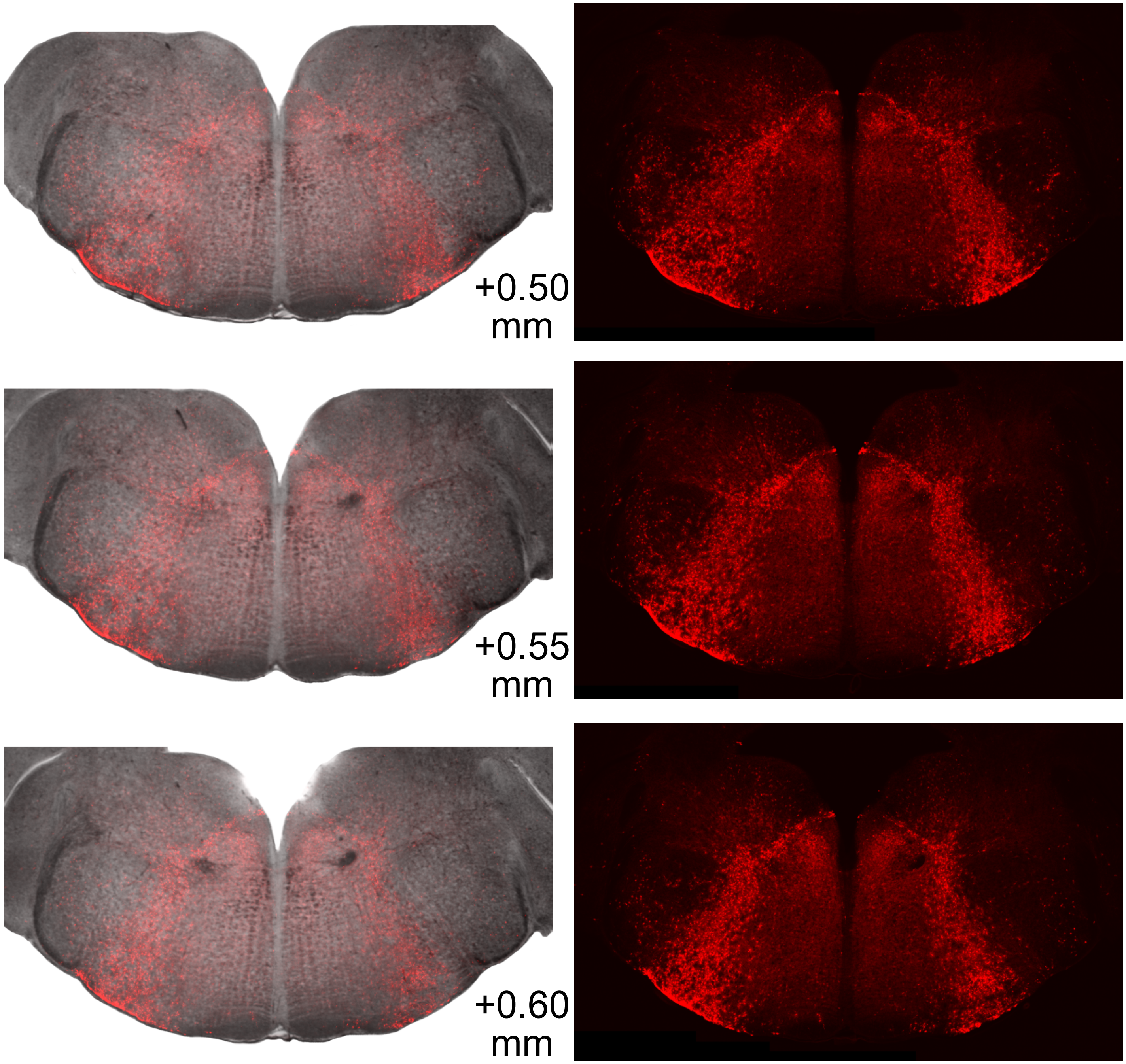
+0.50 mm
+0.55 mm
+0.60 mm
