# Supplementary material for: Identification of the pre‐Bötzinger complex inspiratory center in calibrated “sandwich” slices from newborn mice with fluorescent Dbx1 interneurons
Source: Physiol Rep. 2014 Aug 19;2(8):e12111. doi: 10.14814/phy2.12111 (PMC4246597; doi:10.14814/phy2.12111)
Supplement: Supplementary file 5 — Figure S2. [file phy2-2-e12111-s5.pptx]

## Slide 1
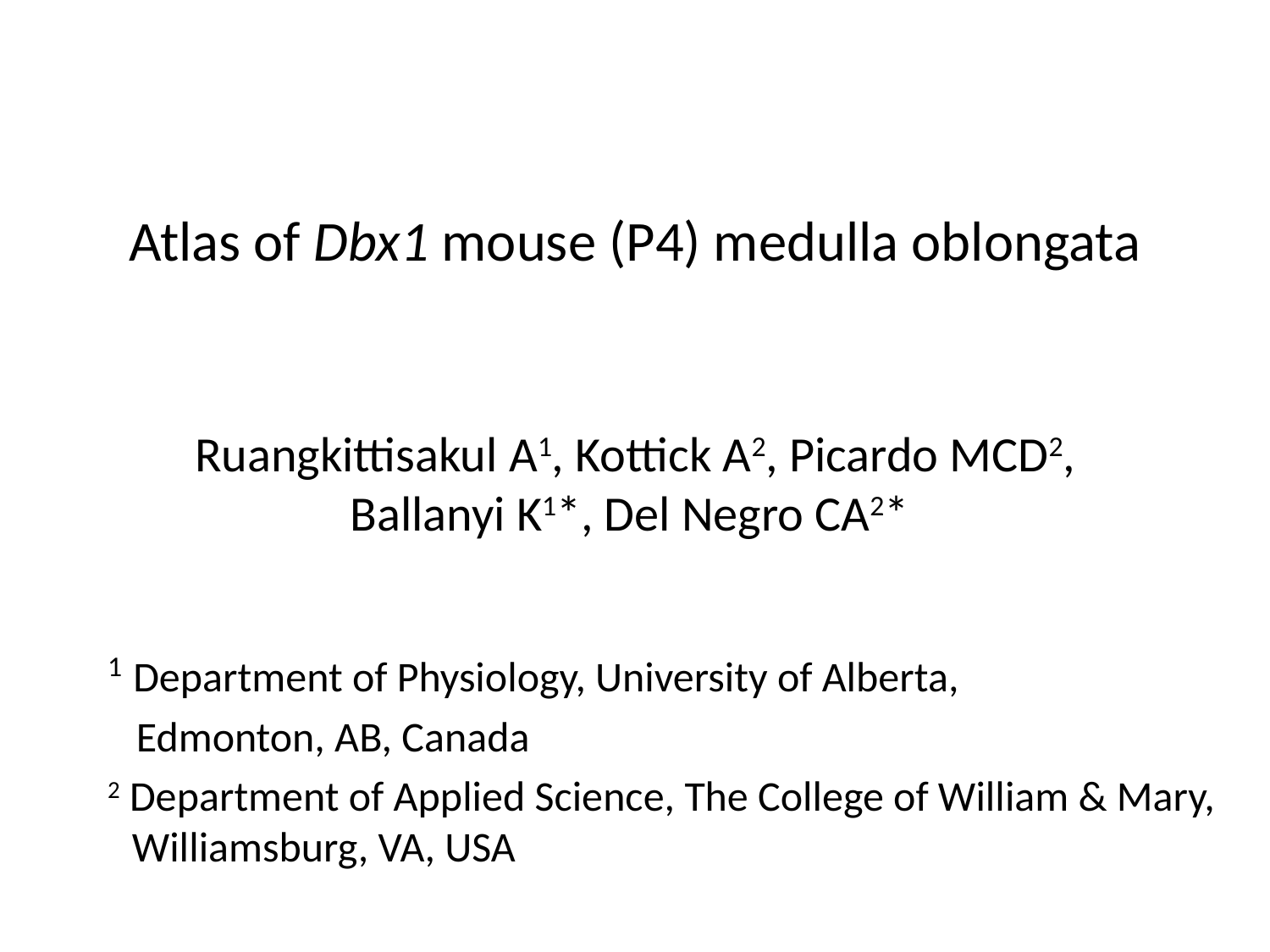

# Atlas of Dbx1 mouse (P4) medulla oblongata
Ruangkittisakul A1, Kottick A2, Picardo MCD2, Ballanyi K1*, Del Negro CA2*
1 Department of Physiology, University of Alberta,
 Edmonton, AB, Canada
2 Department of Applied Science, The College of William & Mary, Williamsburg, VA, USA

## Slide 2
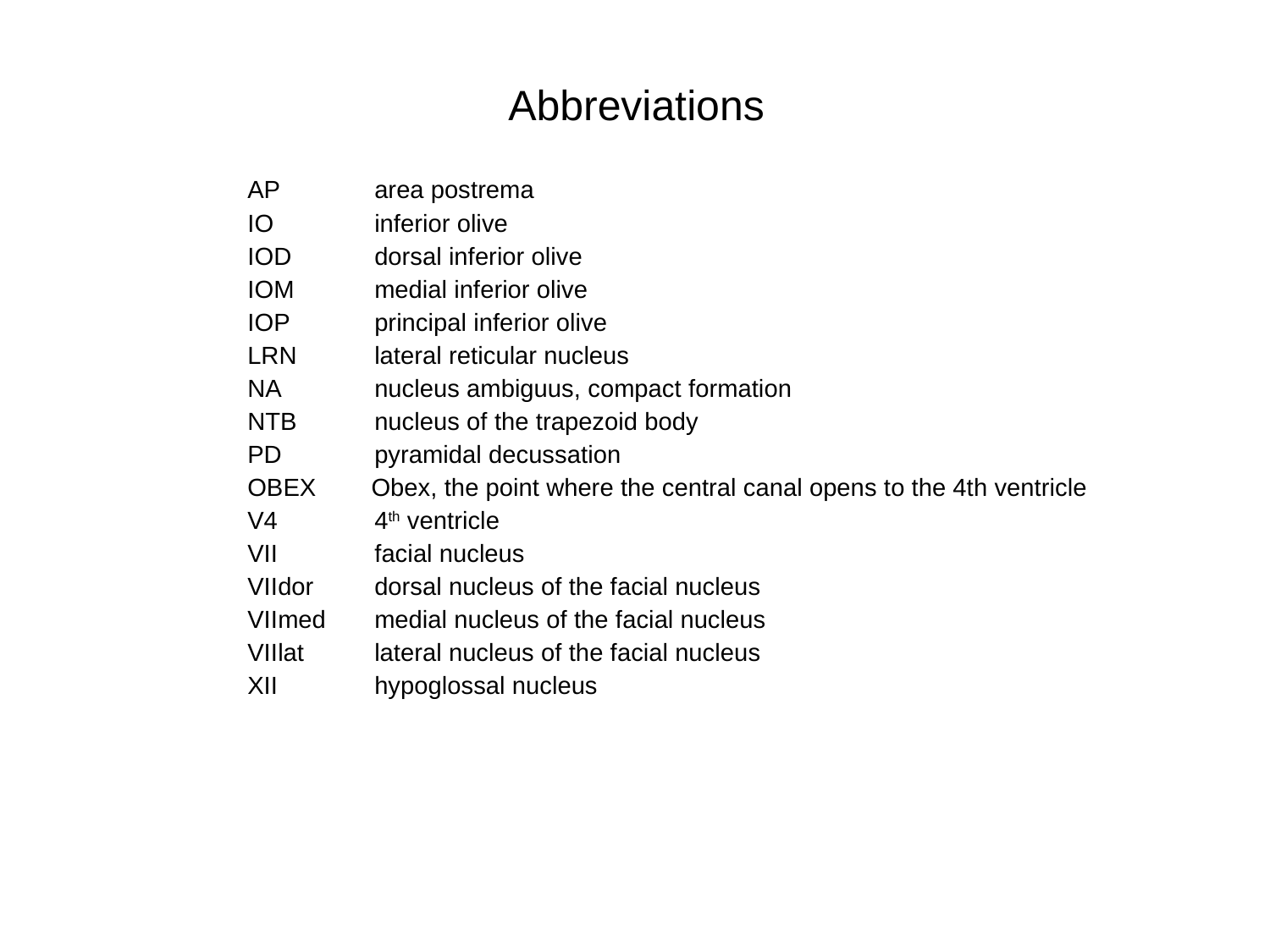

Abbreviations
AP	area postrema
IO	inferior olive
IOD	dorsal inferior olive
IOM	medial inferior olive
IOP	principal inferior olive
LRN	lateral reticular nucleus
NA	nucleus ambiguus, compact formation
NTB	nucleus of the trapezoid body
PD	pyramidal decussation
OBEX	Obex, the point where the central canal opens to the 4th ventricle
V4	4th ventricle
VII	facial nucleus
VIIdor	dorsal nucleus of the facial nucleus
VIImed	medial nucleus of the facial nucleus
VIIlat	lateral nucleus of the facial nucleus
XII	hypoglossal nucleus

## Slide 3
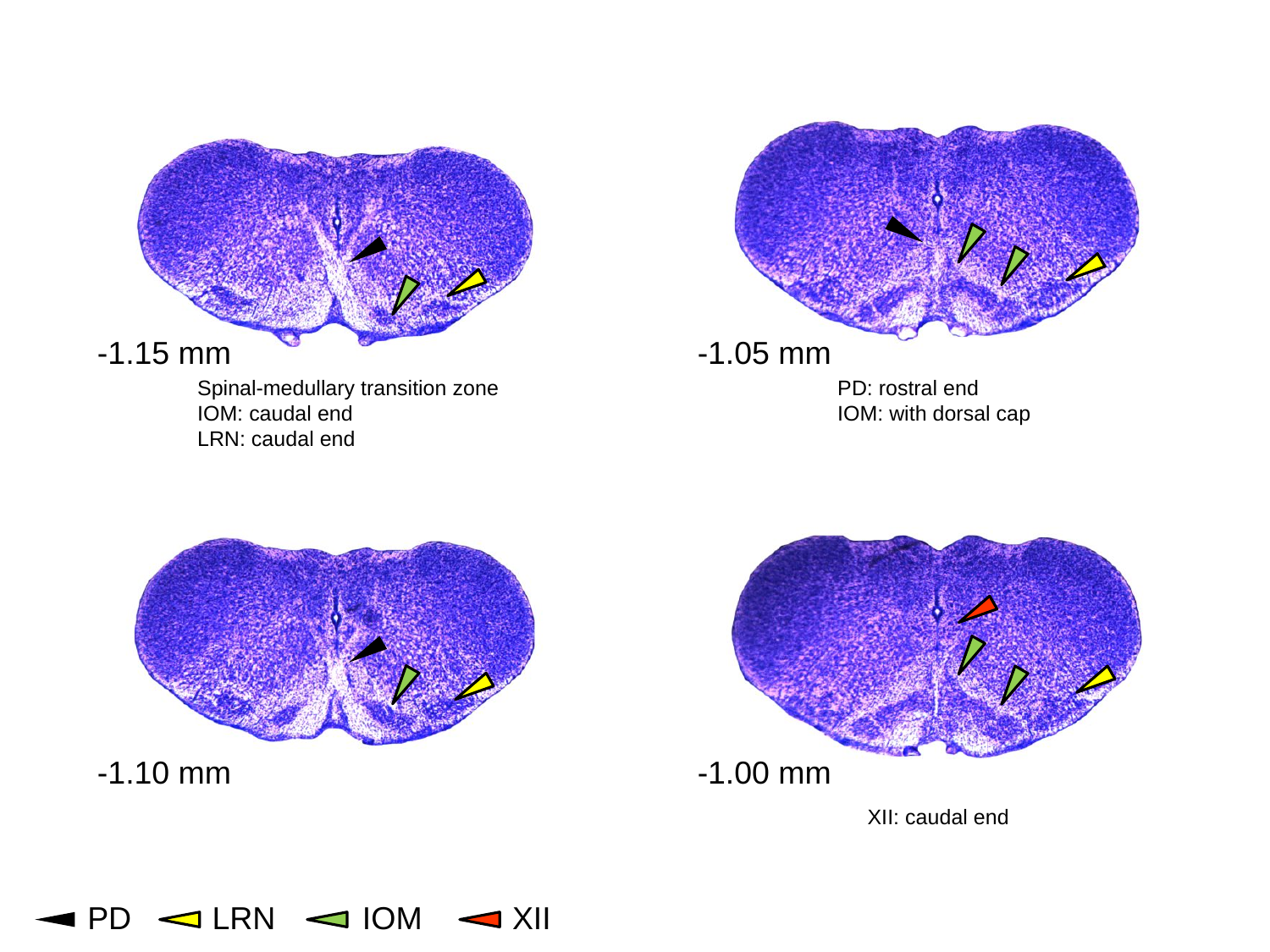

-1.15 mm
-1.05 mm
Spinal-medullary transition zone
IOM: caudal end
LRN: caudal end
PD: rostral end
IOM: with dorsal cap
-1.10 mm
-1.00 mm
XII: caudal end
PD
LRN
IOM
XII

## Slide 4
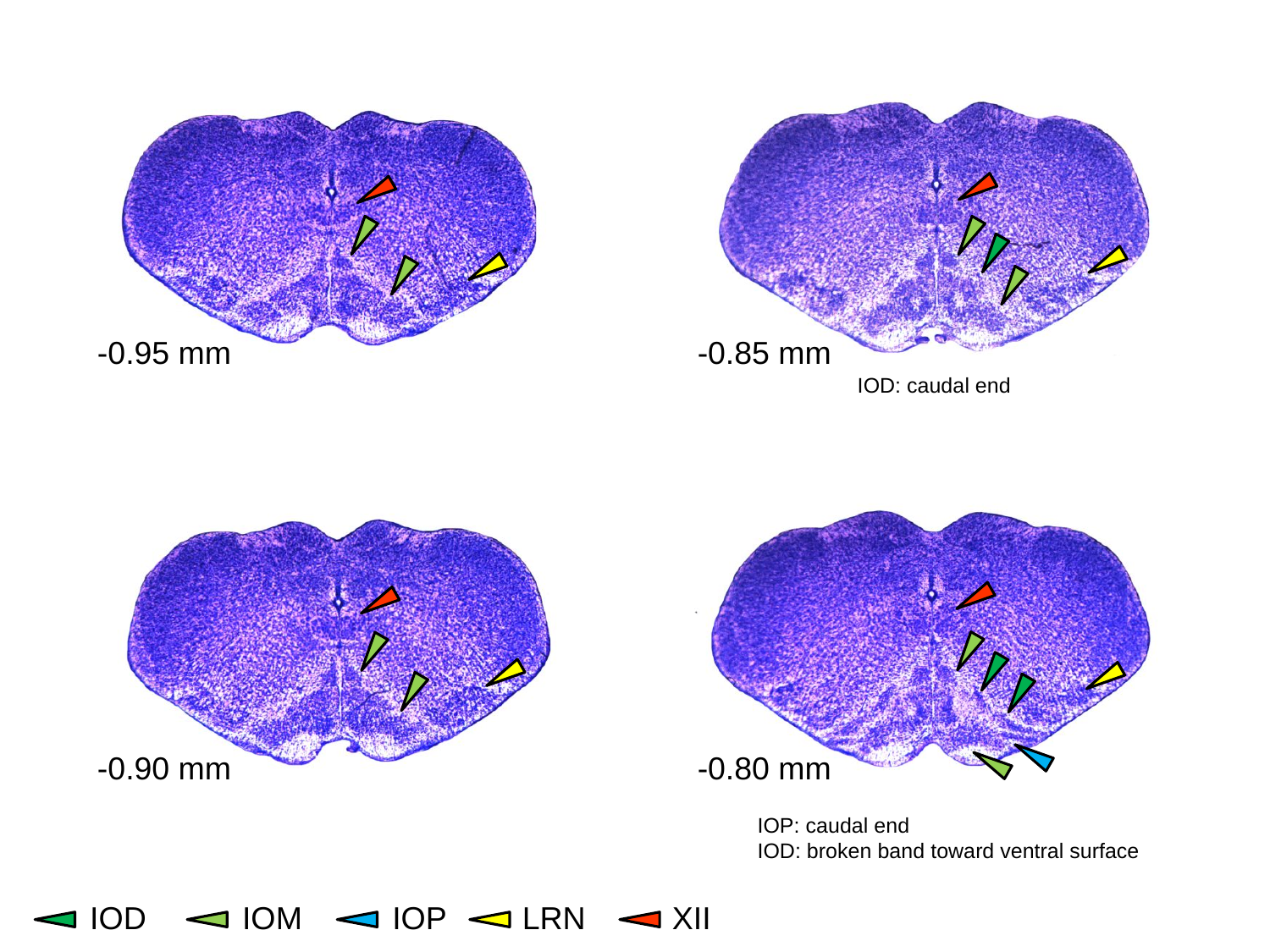

-0.95 mm
-0.85 mm
IOD: caudal end
-0.90 mm
-0.80 mm
IOP: caudal end
IOD: broken band toward ventral surface
IOD
IOM
IOP
LRN
XII

## Slide 5
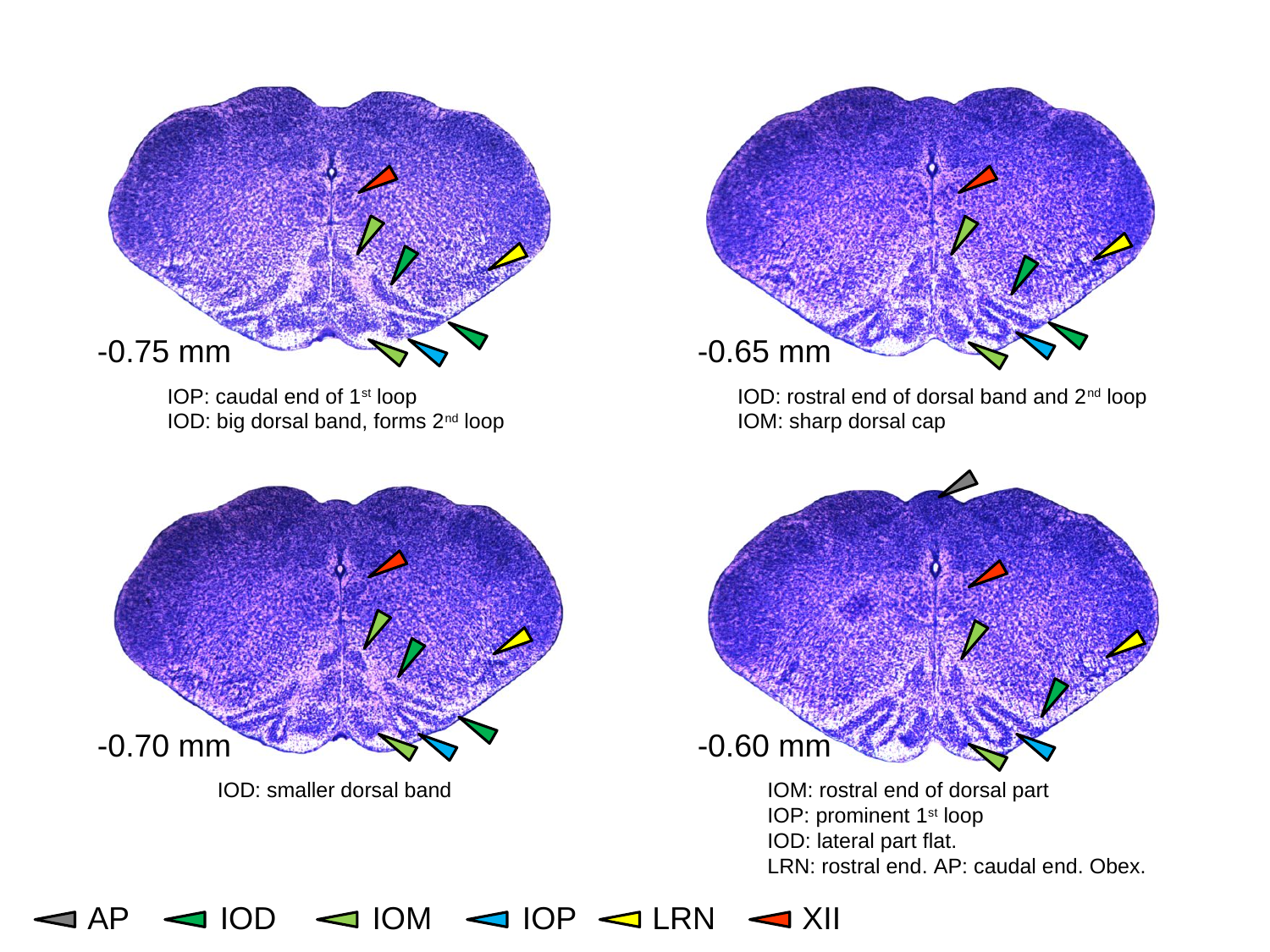

-0.75 mm
-0.65 mm
IOD: rostral end of dorsal band and 2nd loop
IOM: sharp dorsal cap
IOP: caudal end of 1st loop
IOD: big dorsal band, forms 2nd loop
-0.70 mm
-0.60 mm
IOD: smaller dorsal band
IOM: rostral end of dorsal part
IOP: prominent 1st loop
IOD: lateral part flat.
LRN: rostral end. AP: caudal end. Obex.
AP
IOD
IOM
IOP
LRN
XII

## Slide 6
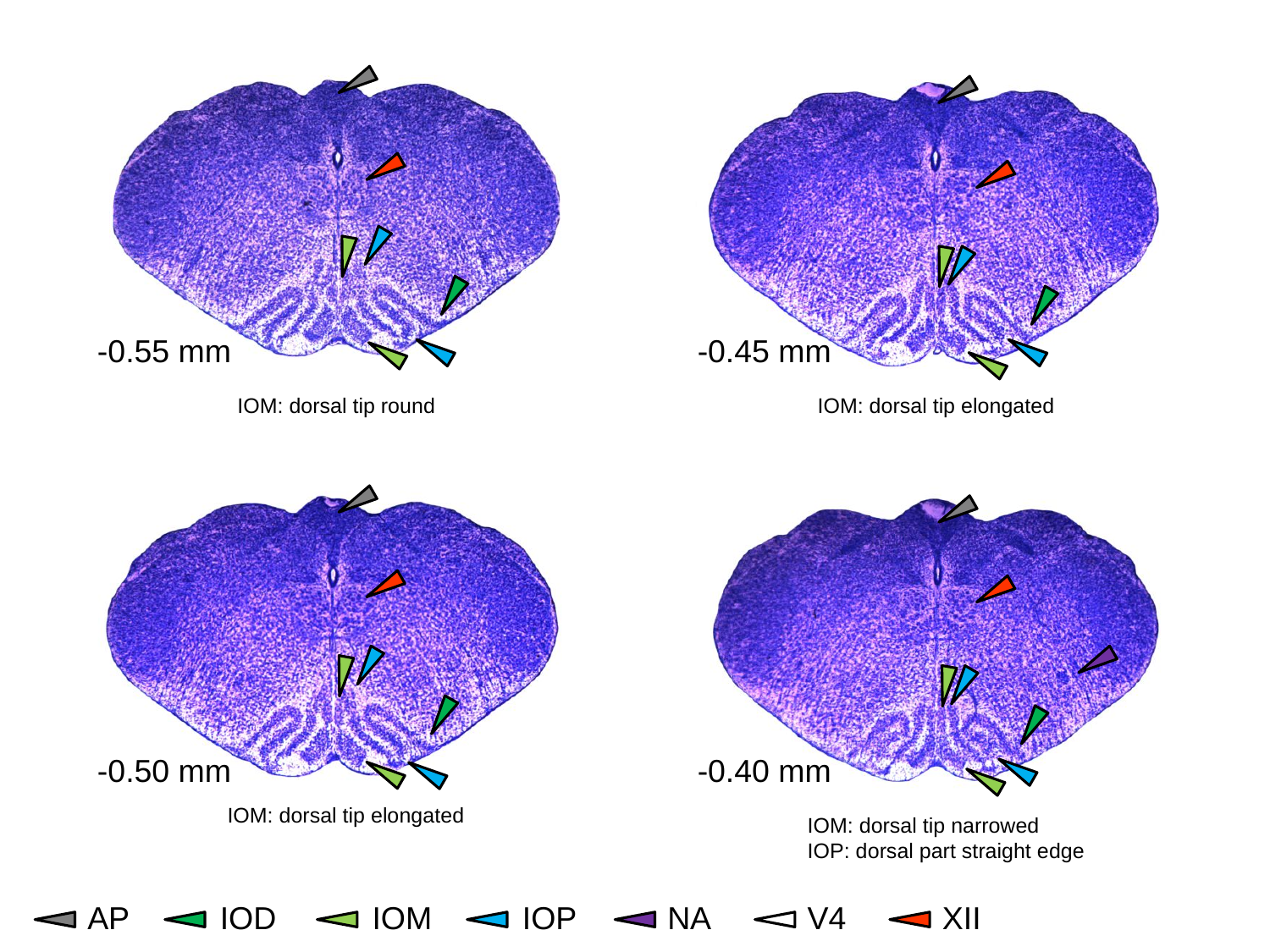

-0.55 mm
-0.45 mm
IOM: dorsal tip round
IOM: dorsal tip elongated
-0.50 mm
-0.40 mm
IOM: dorsal tip elongated
IOM: dorsal tip narrowed
IOP: dorsal part straight edge
AP
IOD
IOM
IOP
NA
V4
XII

## Slide 7
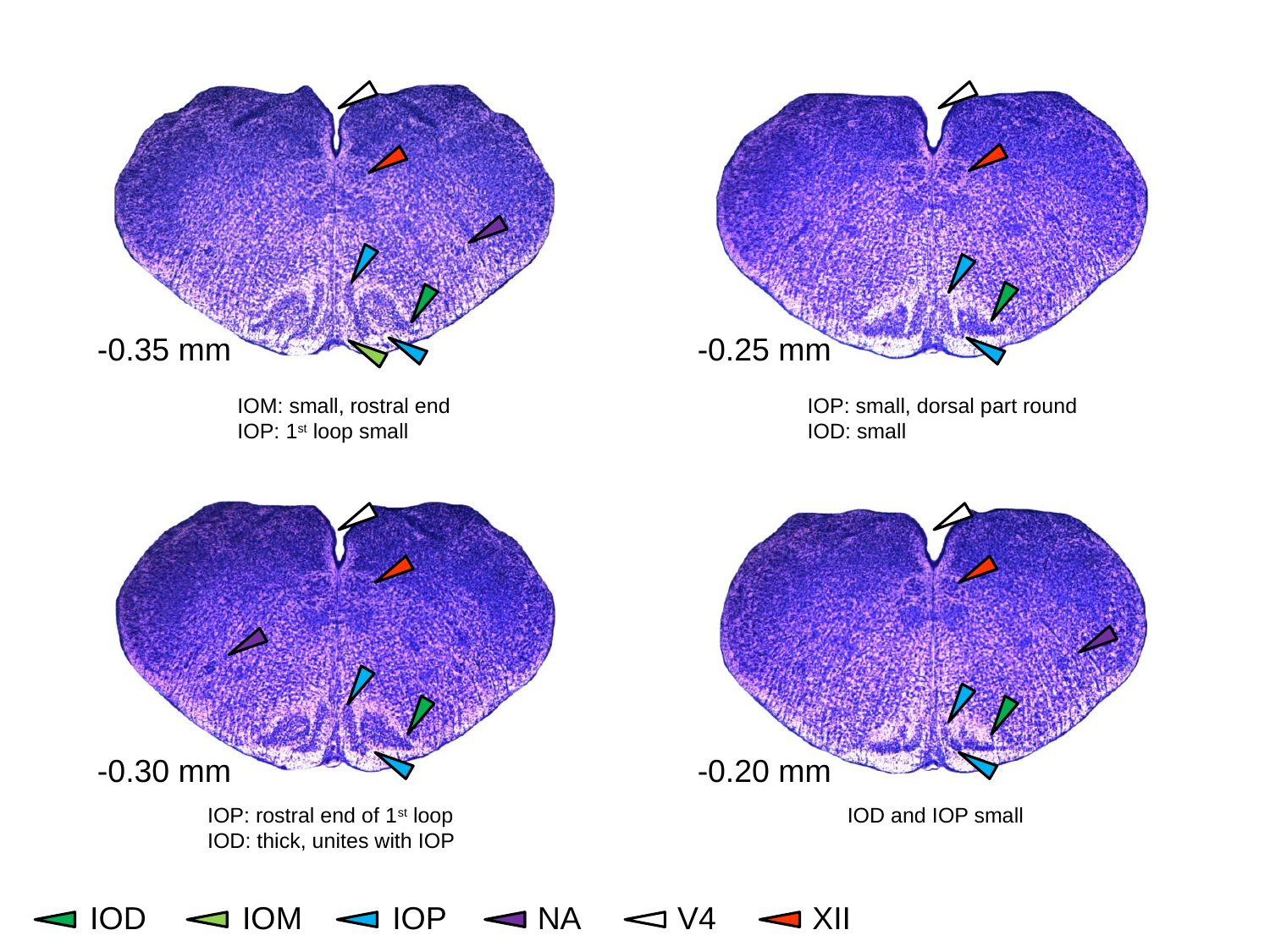

-0.35 mm
-0.25 mm
IOM: small, rostral end
IOP: 1st loop small
IOP: small, dorsal part round
IOD: small
-0.30 mm
-0.20 mm
IOP: rostral end of 1st loop
IOD: thick, unites with IOP
IOD and IOP small
IOD
IOM
IOP
NA
V4
XII

## Slide 8
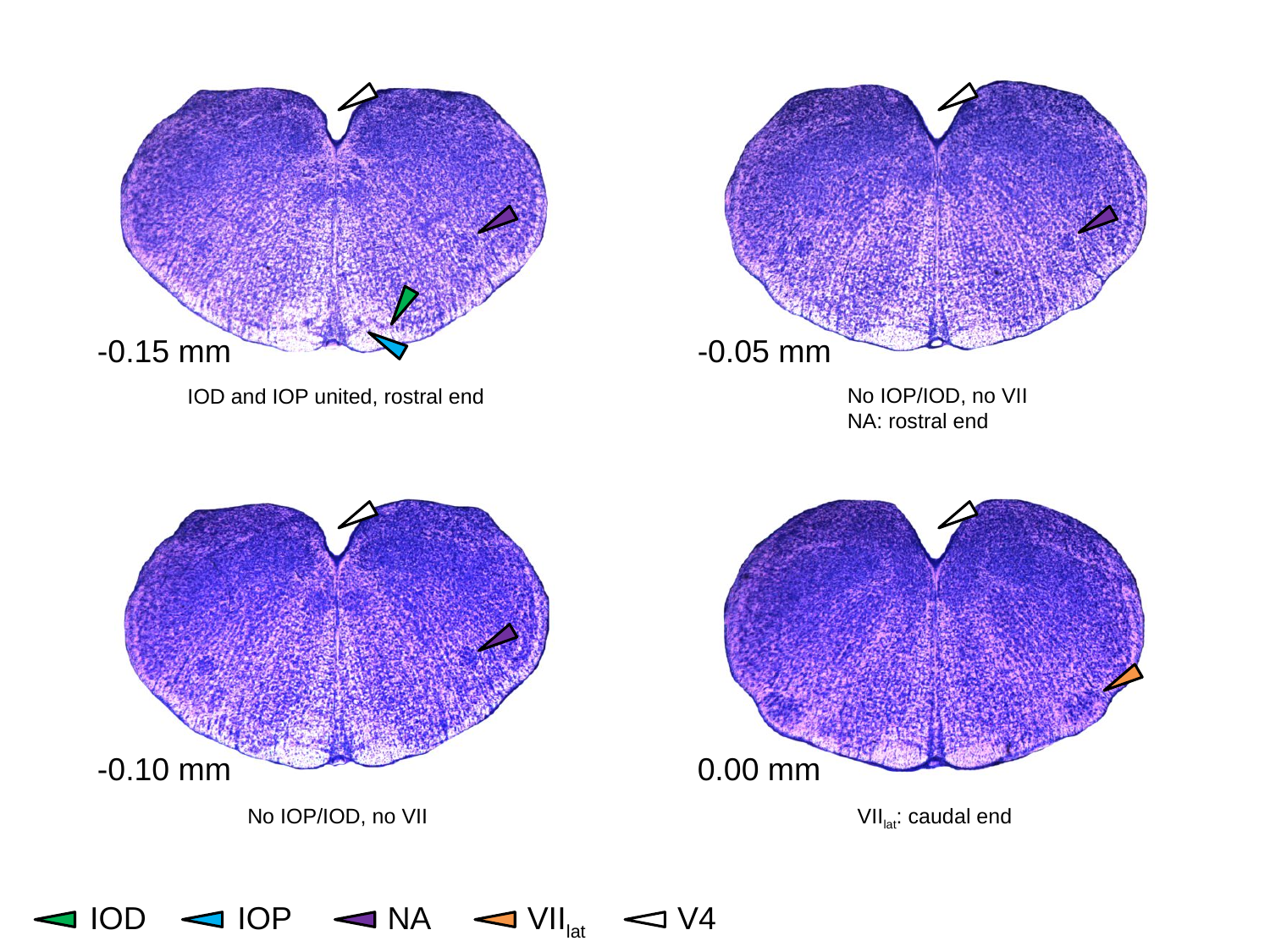

-0.15 mm
-0.05 mm
No IOP/IOD, no VII
NA: rostral end
IOD and IOP united, rostral end
-0.10 mm
0.00 mm
No IOP/IOD, no VII
VIIlat: caudal end
IOD
IOP
NA
VIIlat
V4

## Slide 9
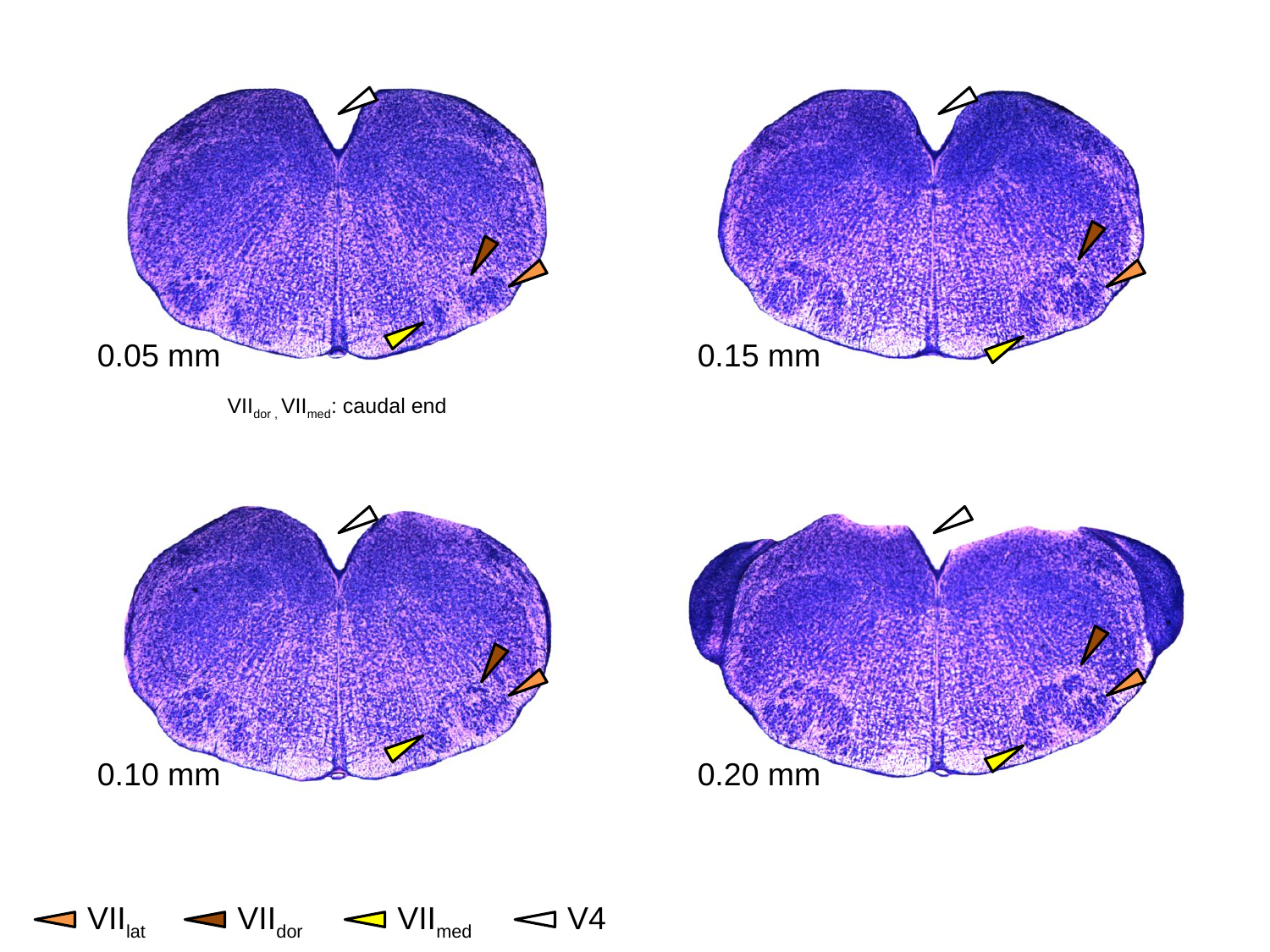

0.05 mm
0.15 mm
VIIdor , VIImed: caudal end
0.10 mm
0.20 mm
VIIlat
VIIdor
VIImed
V4

## Slide 10
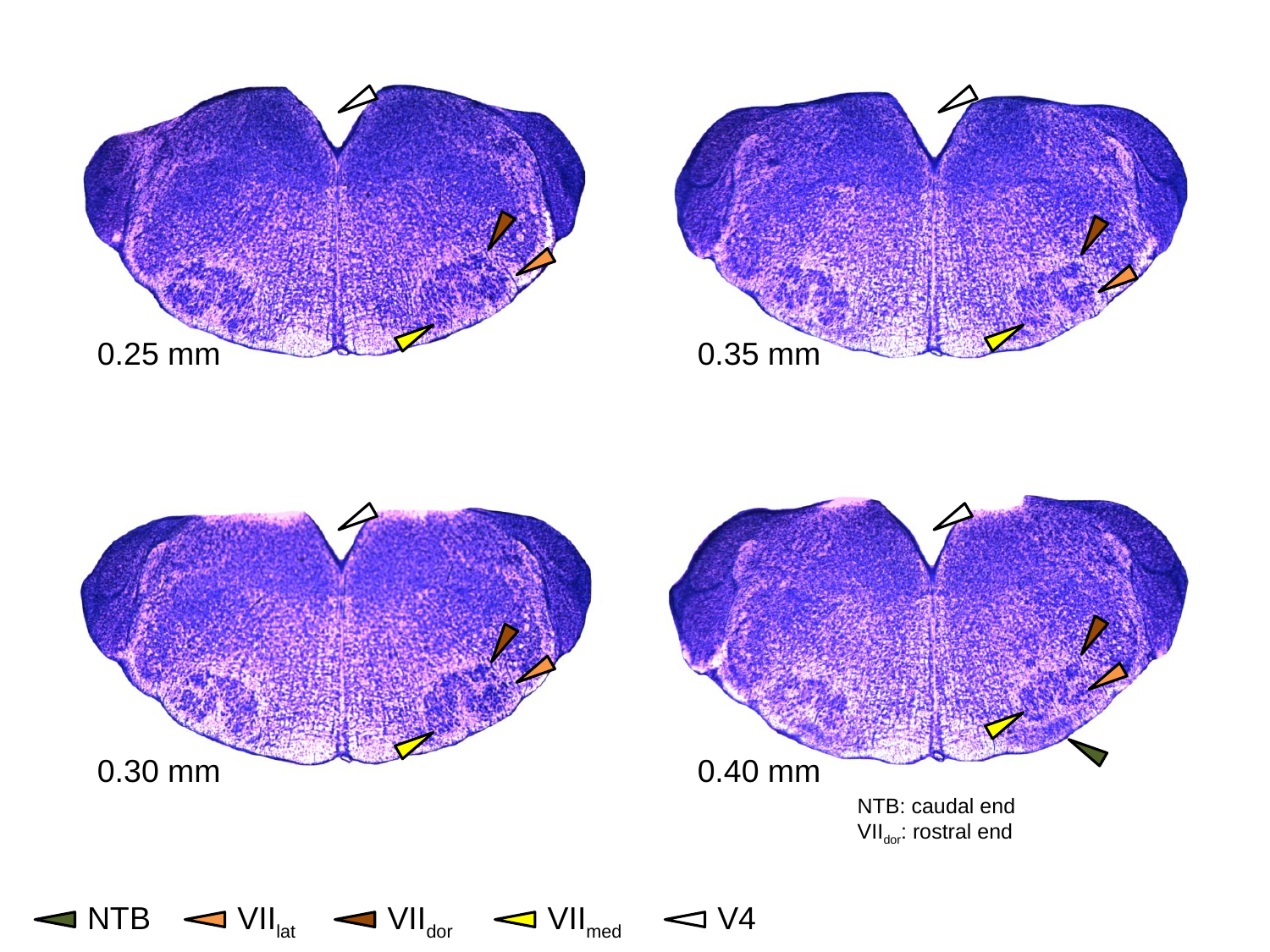

0.25 mm
0.35 mm
0.30 mm
0.40 mm
NTB: caudal end
VIIdor: rostral end
NTB
VIIlat
VIIdor
VIImed
V4

## Slide 11
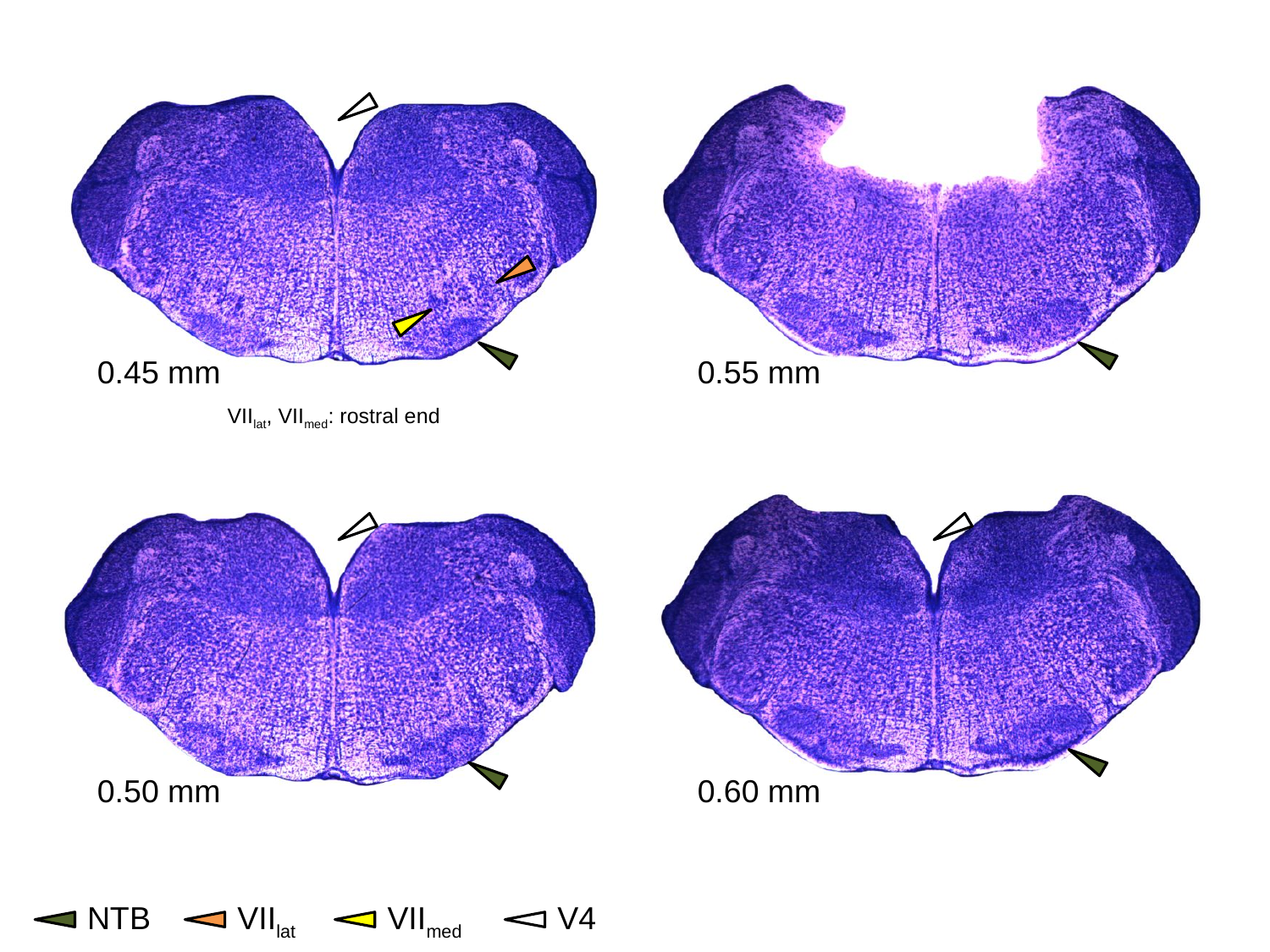

0.45 mm
0.55 mm
VIIlat, VIImed: rostral end
0.50 mm
0.60 mm
NTB
VIIlat
VIImed
V4
