# Supplementary material for: Identification of the pre‐Bötzinger complex inspiratory center in calibrated “sandwich” slices from newborn mice with fluorescent Dbx1 interneurons
Source: Physiol Rep. 2014 Aug 19;2(8):e12111. doi: 10.14814/phy2.12111 (PMC4246597; doi:10.14814/phy2.12111)
Supplement: Supplementary file 6 — Figure S3. [file phy2-2-e12111-s6.pptx]

## Slide 1
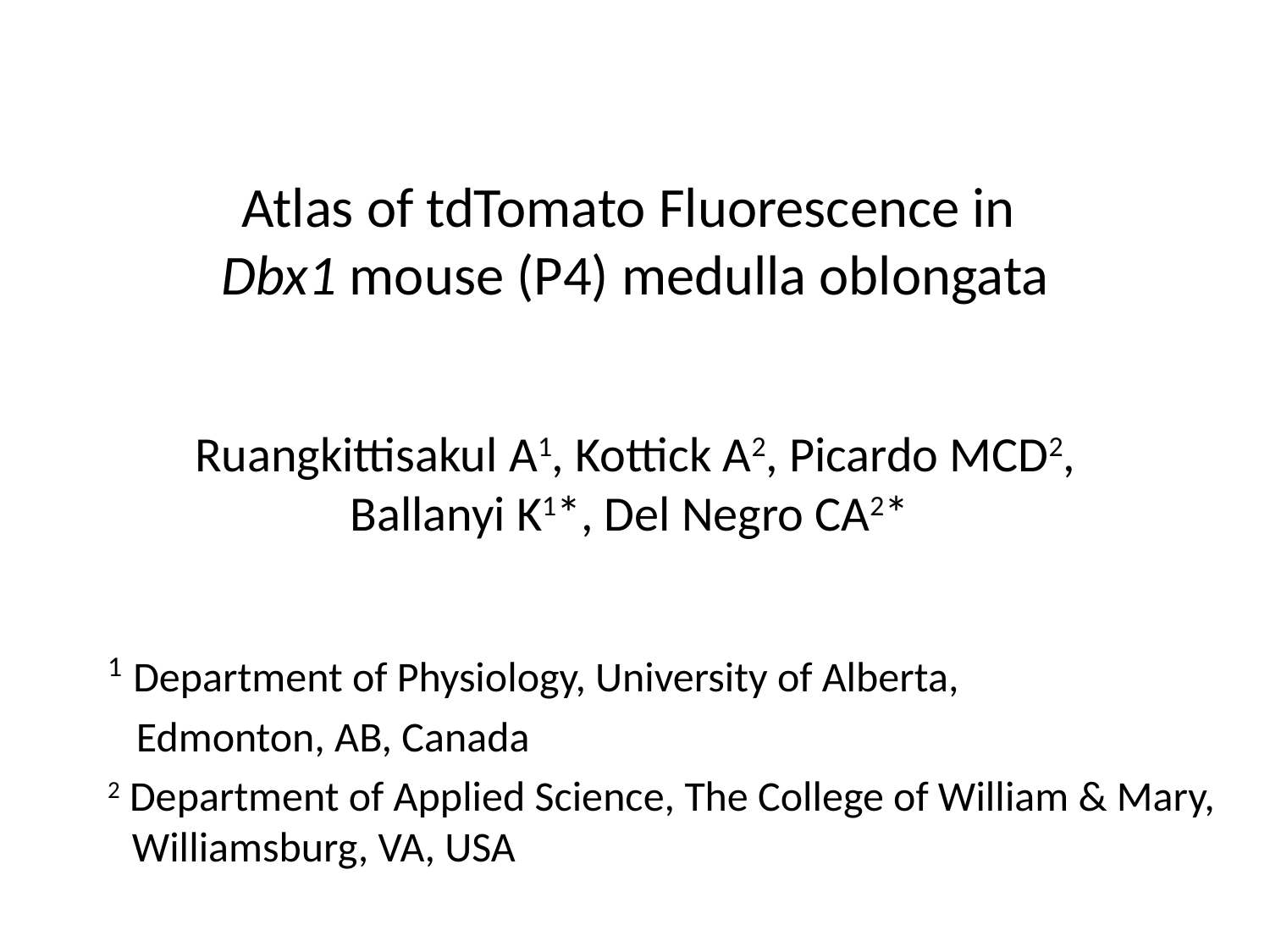

# Atlas of tdTomato Fluorescence in Dbx1 mouse (P4) medulla oblongata
Ruangkittisakul A1, Kottick A2, Picardo MCD2, Ballanyi K1*, Del Negro CA2*
1 Department of Physiology, University of Alberta,
 Edmonton, AB, Canada
2 Department of Applied Science, The College of William & Mary, Williamsburg, VA, USA

## Slide 2
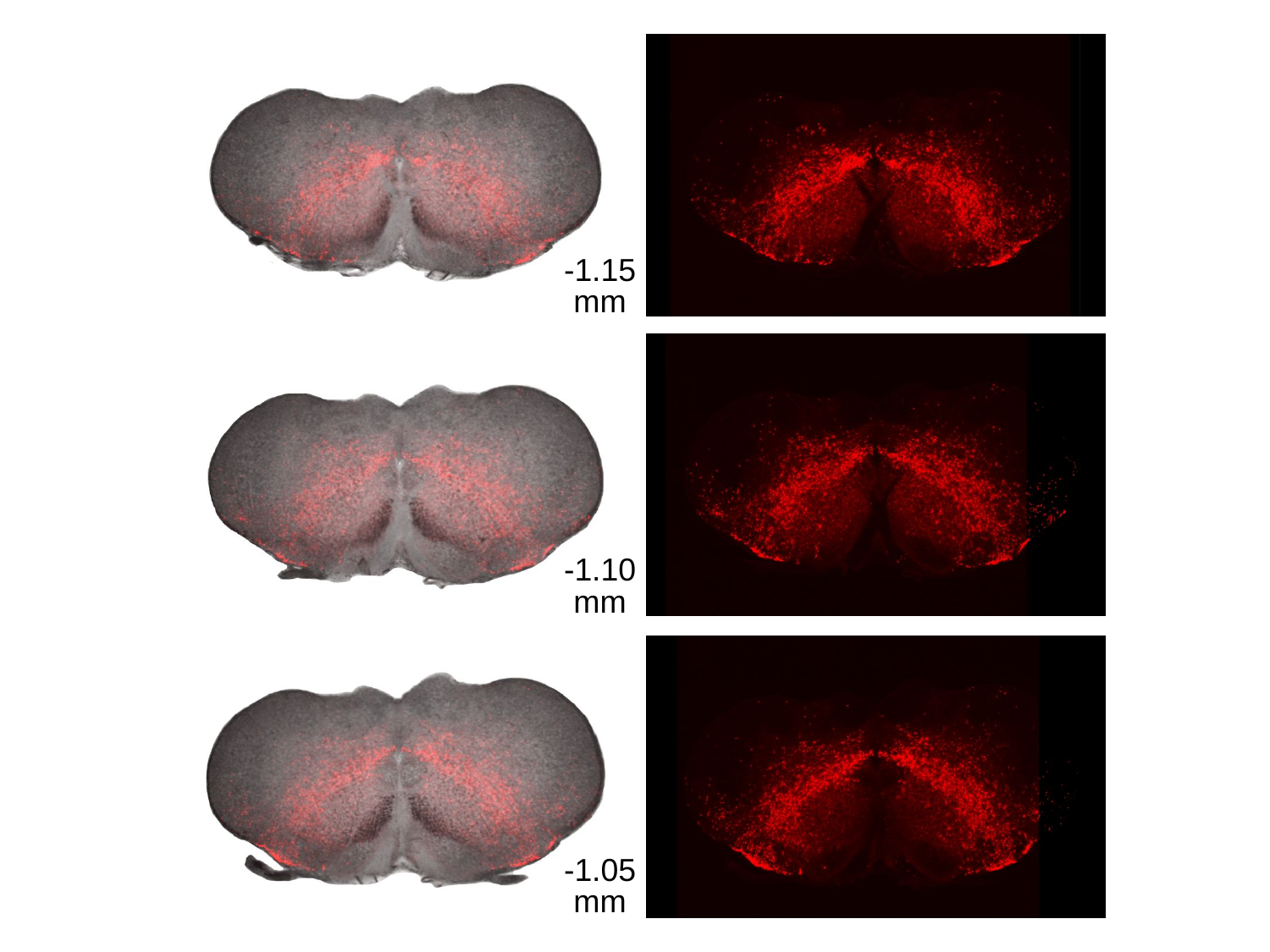

-1.15
mm
-1.10
mm
-1.05
mm

## Slide 3
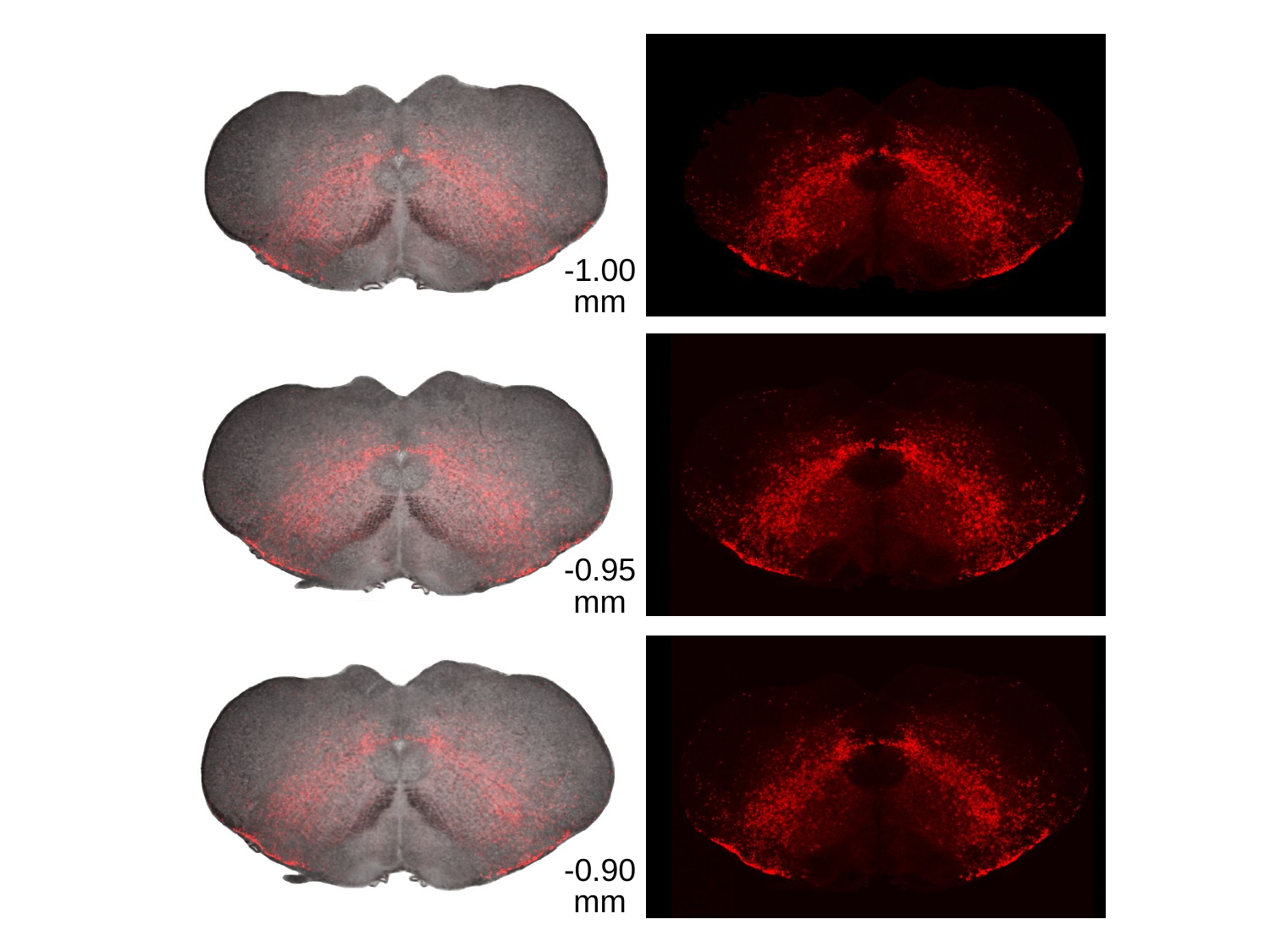

-1.00
mm
-0.95
mm
-0.90
mm

## Slide 4
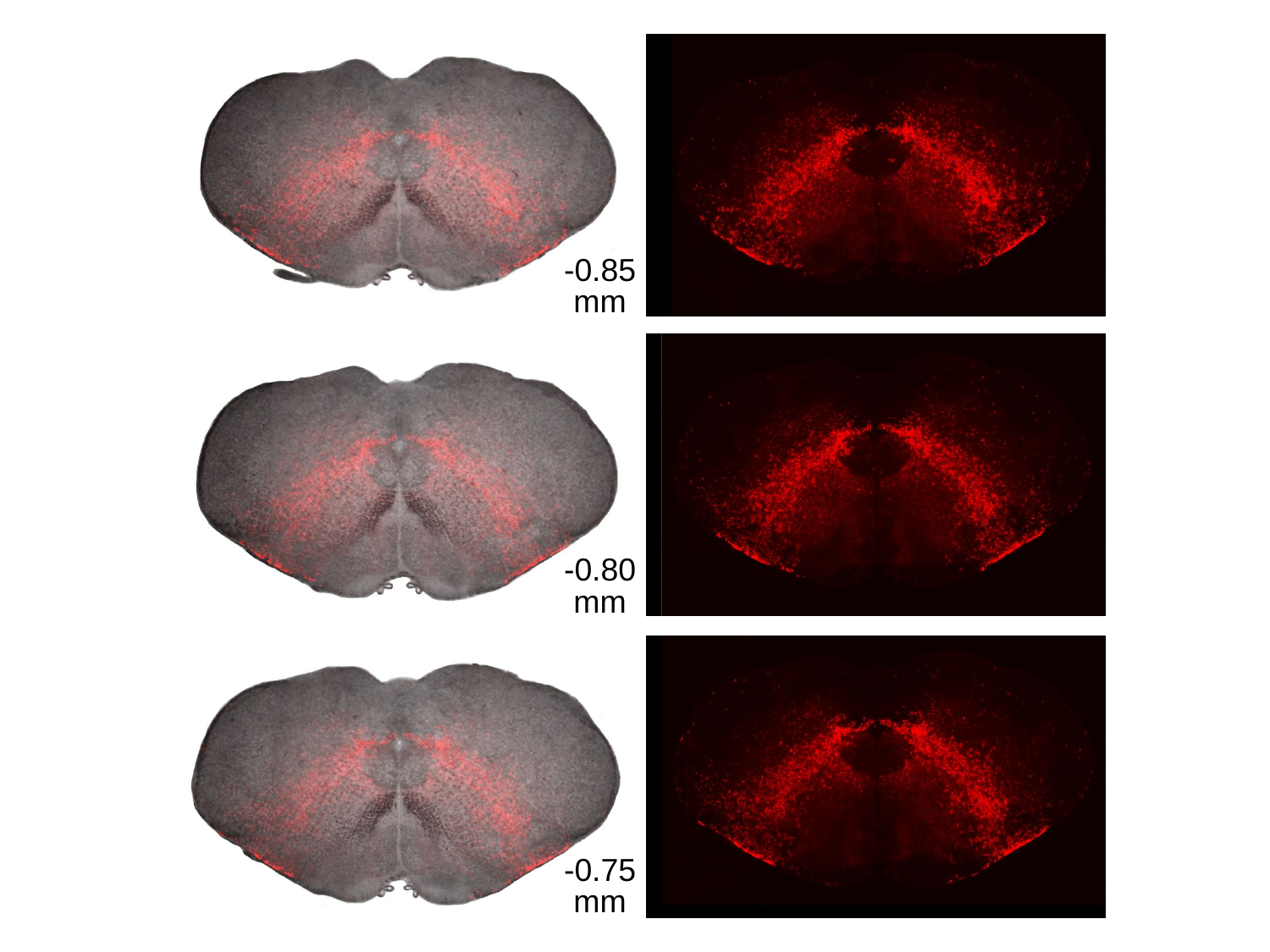

-0.85
mm
-0.80
mm
-0.75
mm

## Slide 5
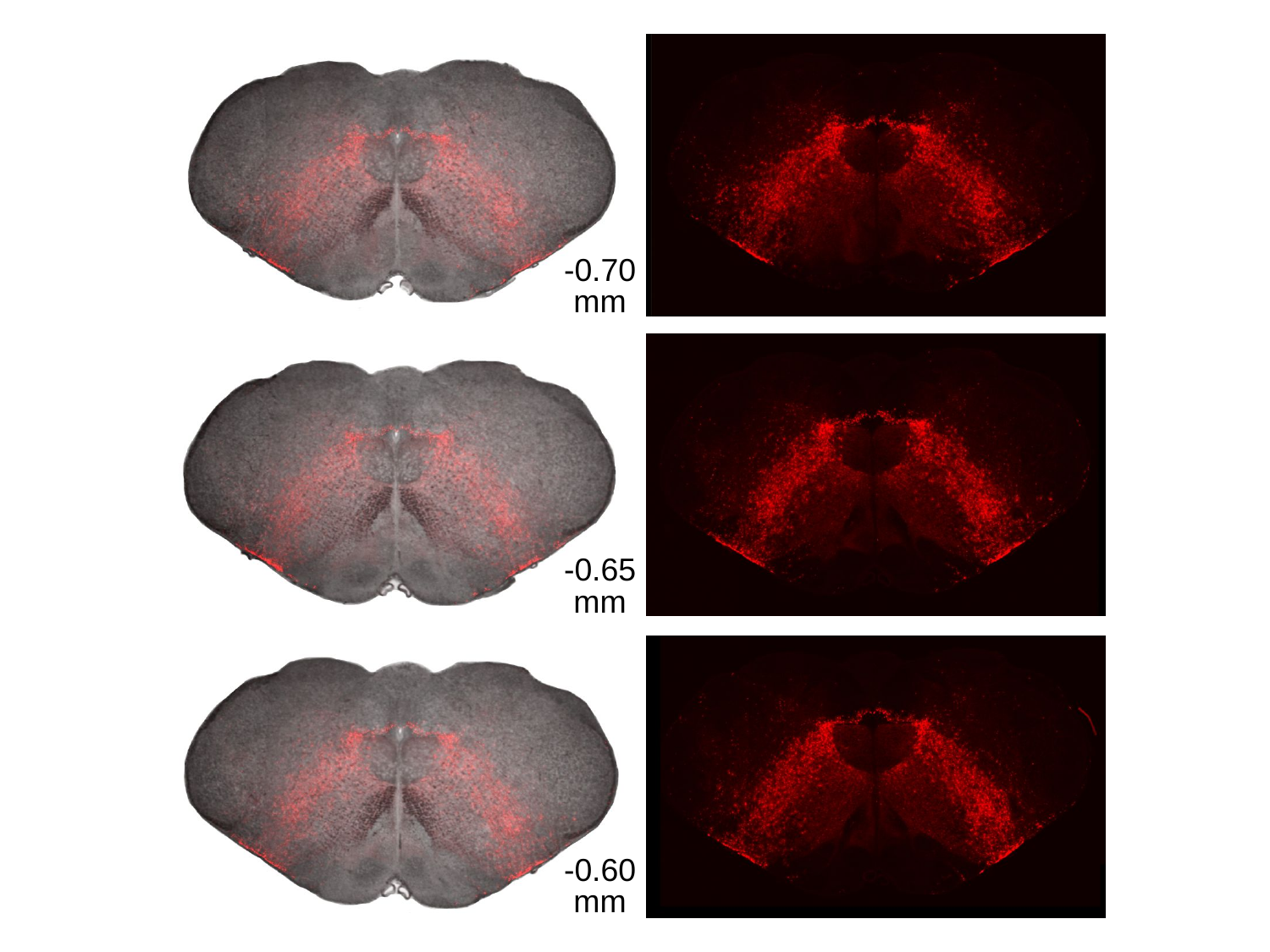

-0.70
mm
-0.65
mm
-0.60
mm

## Slide 6
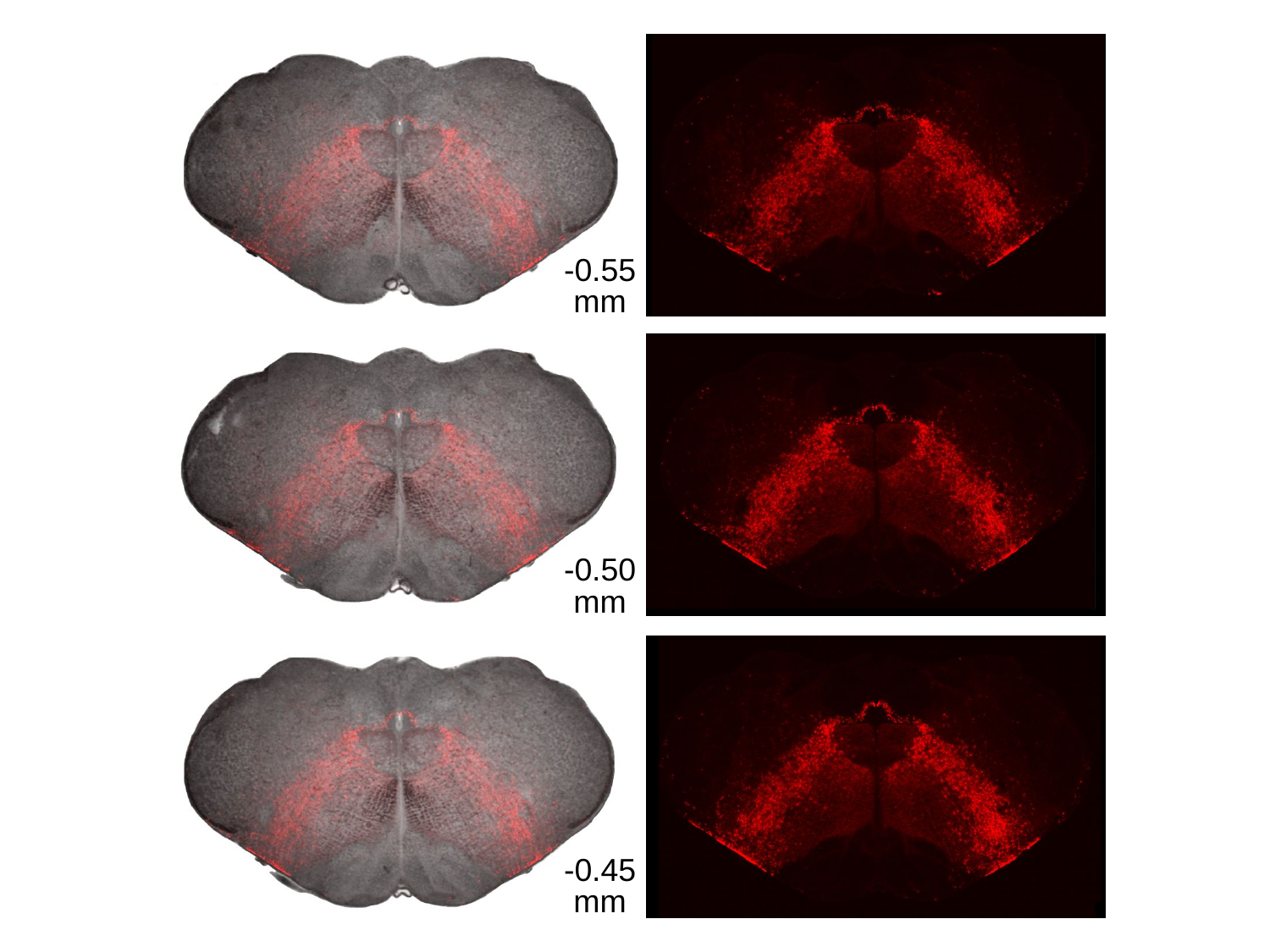

-0.55
mm
-0.50
mm
-0.45
mm

## Slide 7
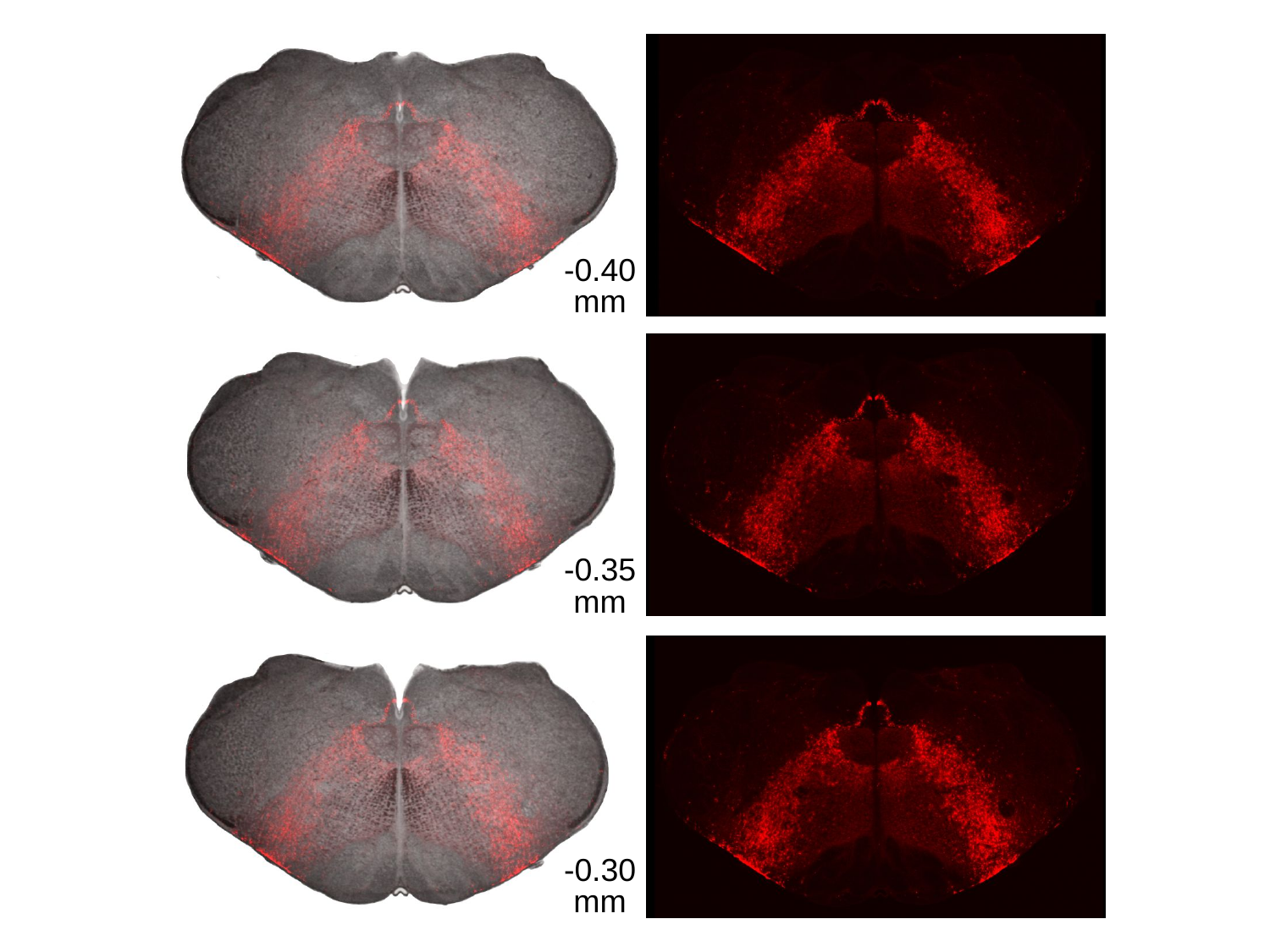

-0.40
mm
-0.35
mm
-0.30
mm

## Slide 8
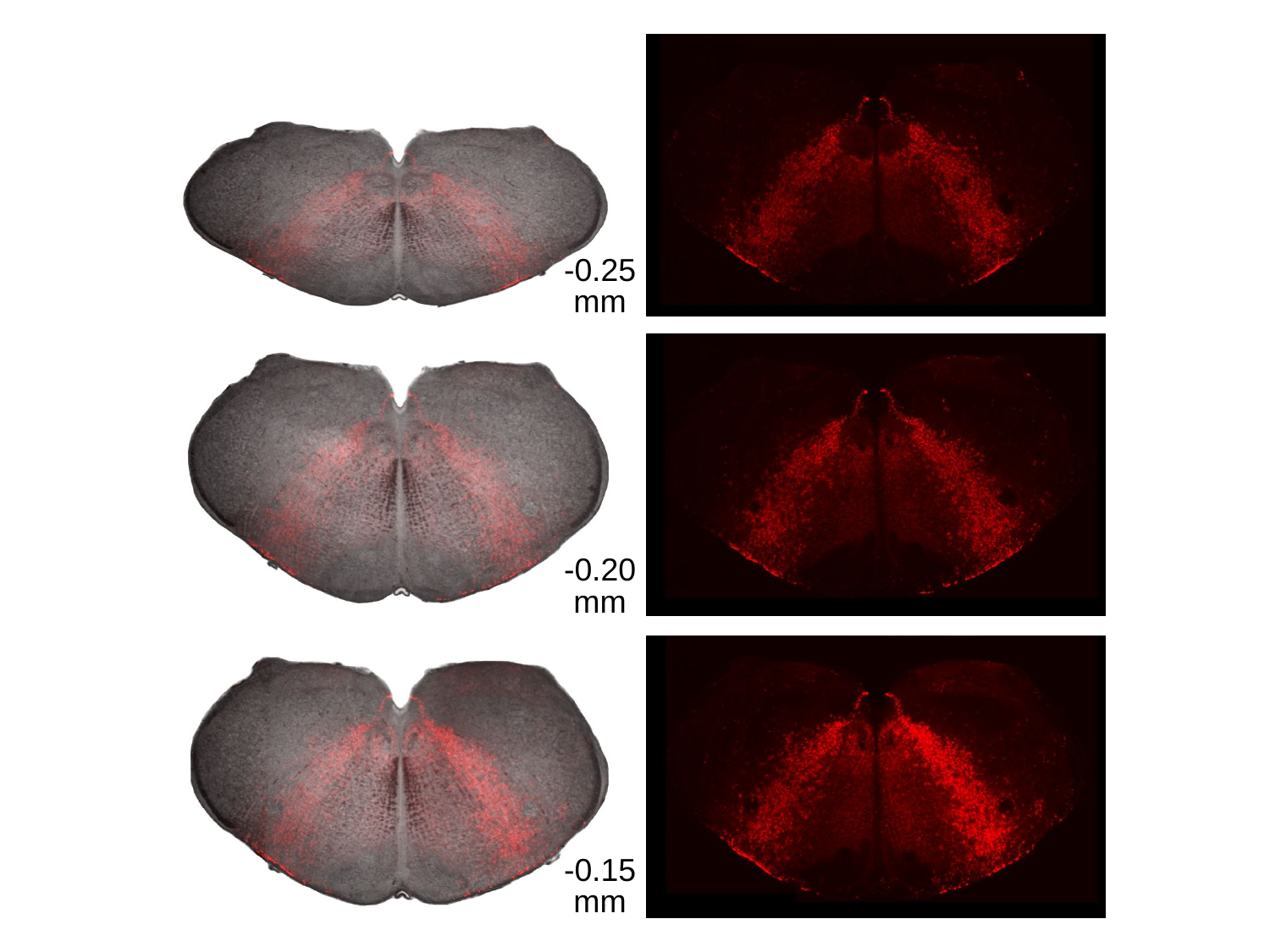

-0.25
mm
-0.20
mm
-0.15
mm

## Slide 9
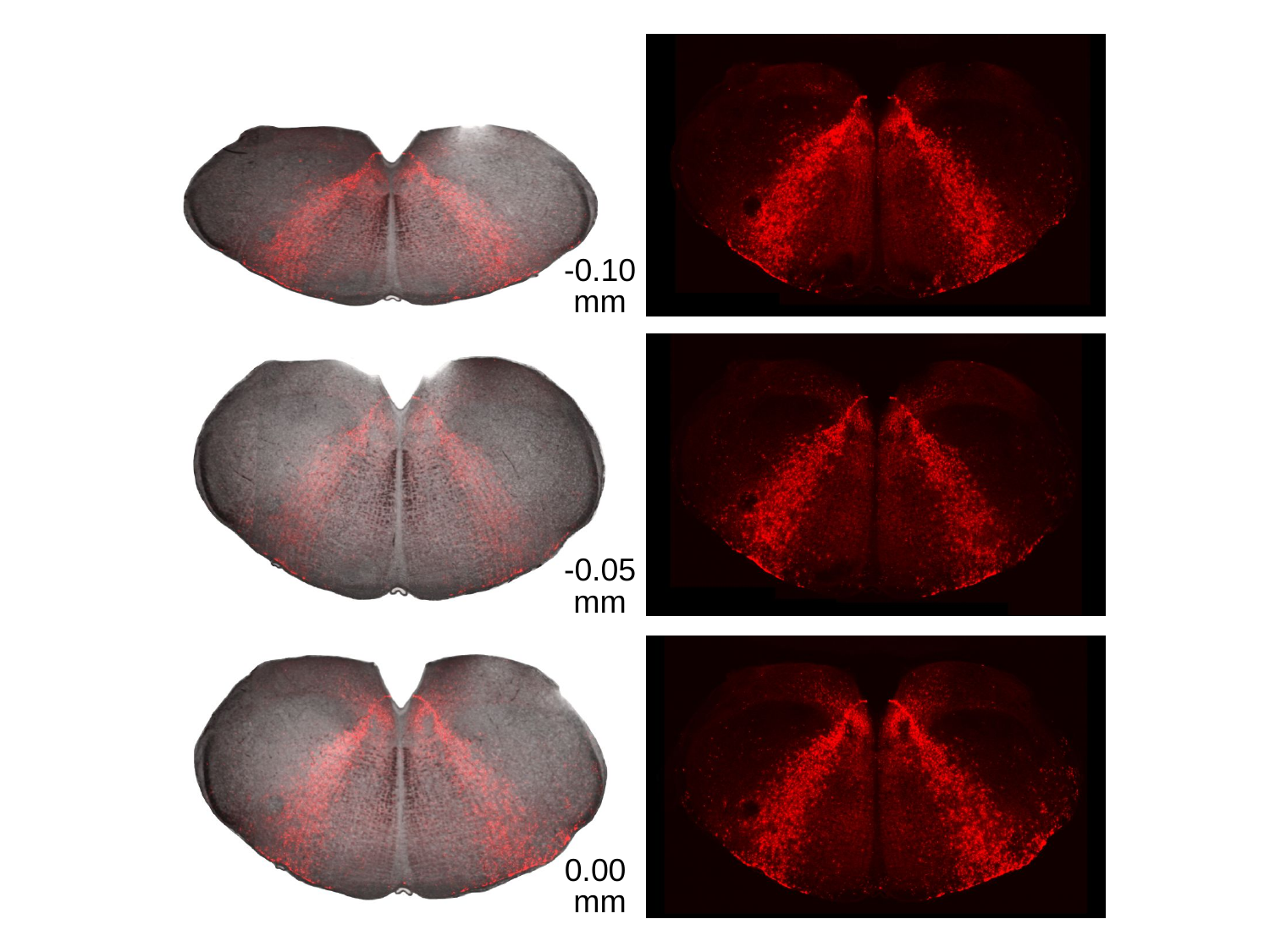

-0.10
mm
-0.05
mm
0.00
mm

## Slide 10
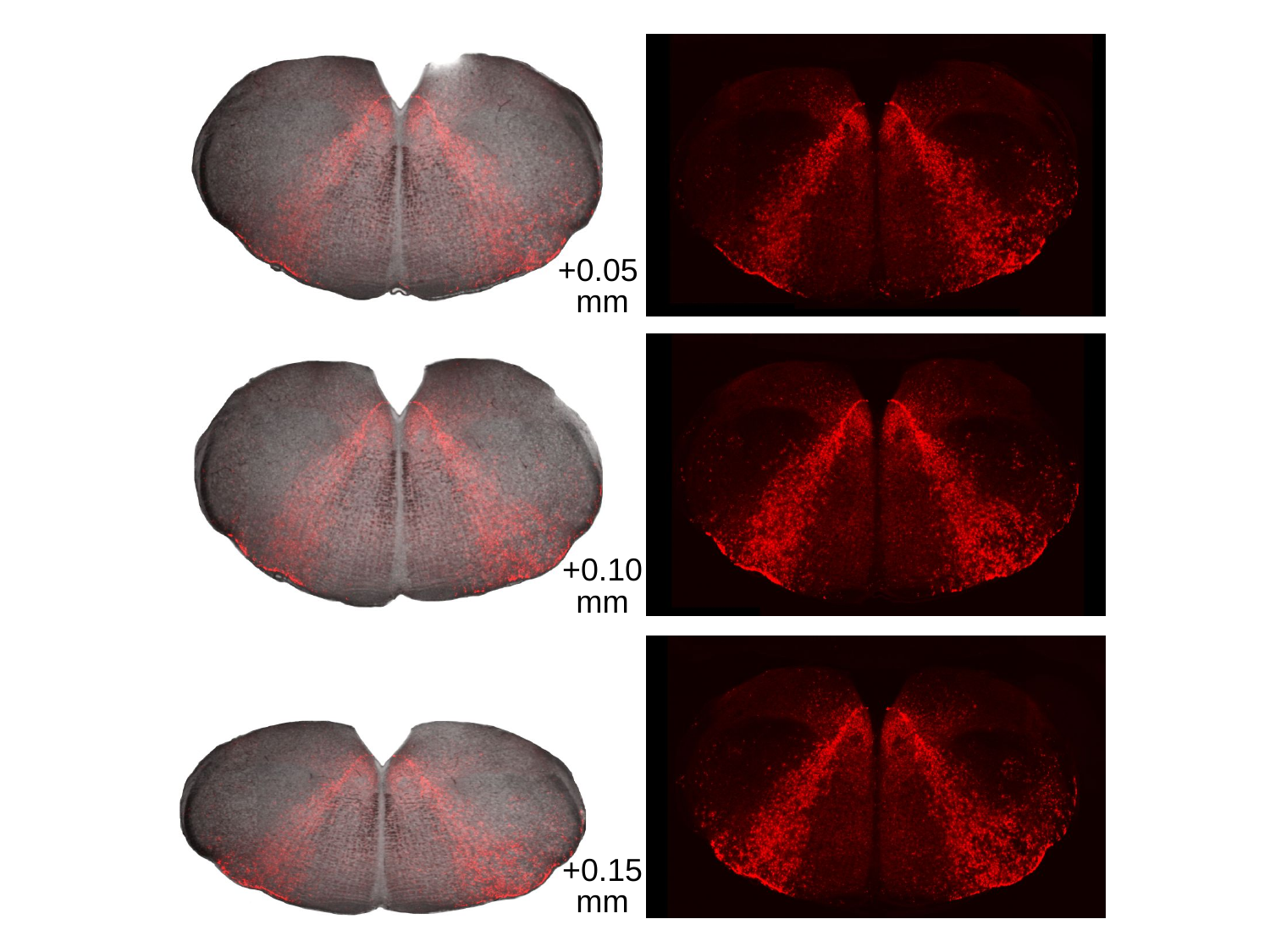

+0.05
mm
+0.10
mm
+0.15
mm

## Slide 11
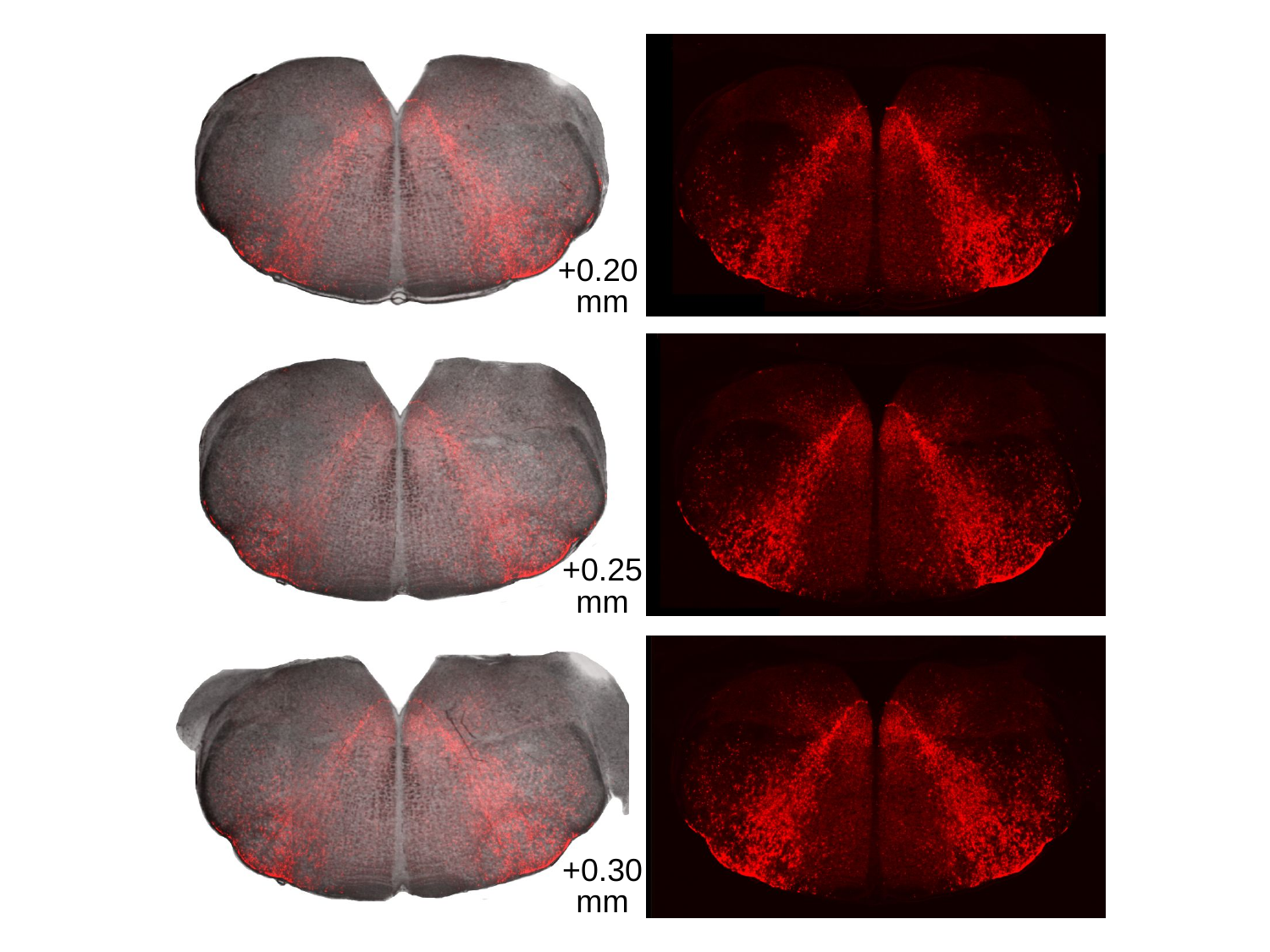

+0.20
mm
+0.25
mm
+0.30
mm

## Slide 12
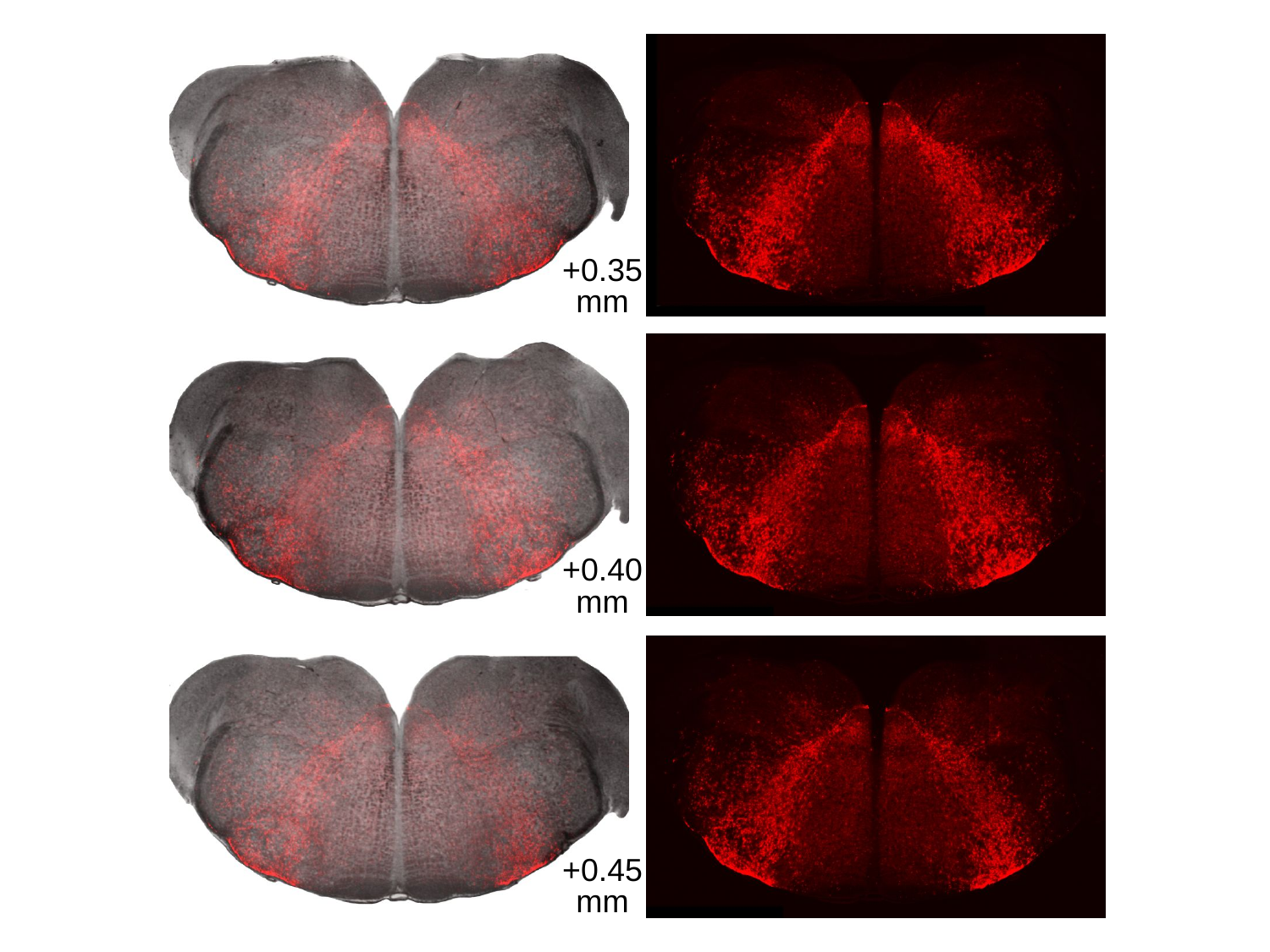

+0.35
mm
+0.40
mm
+0.45
mm

## Slide 13
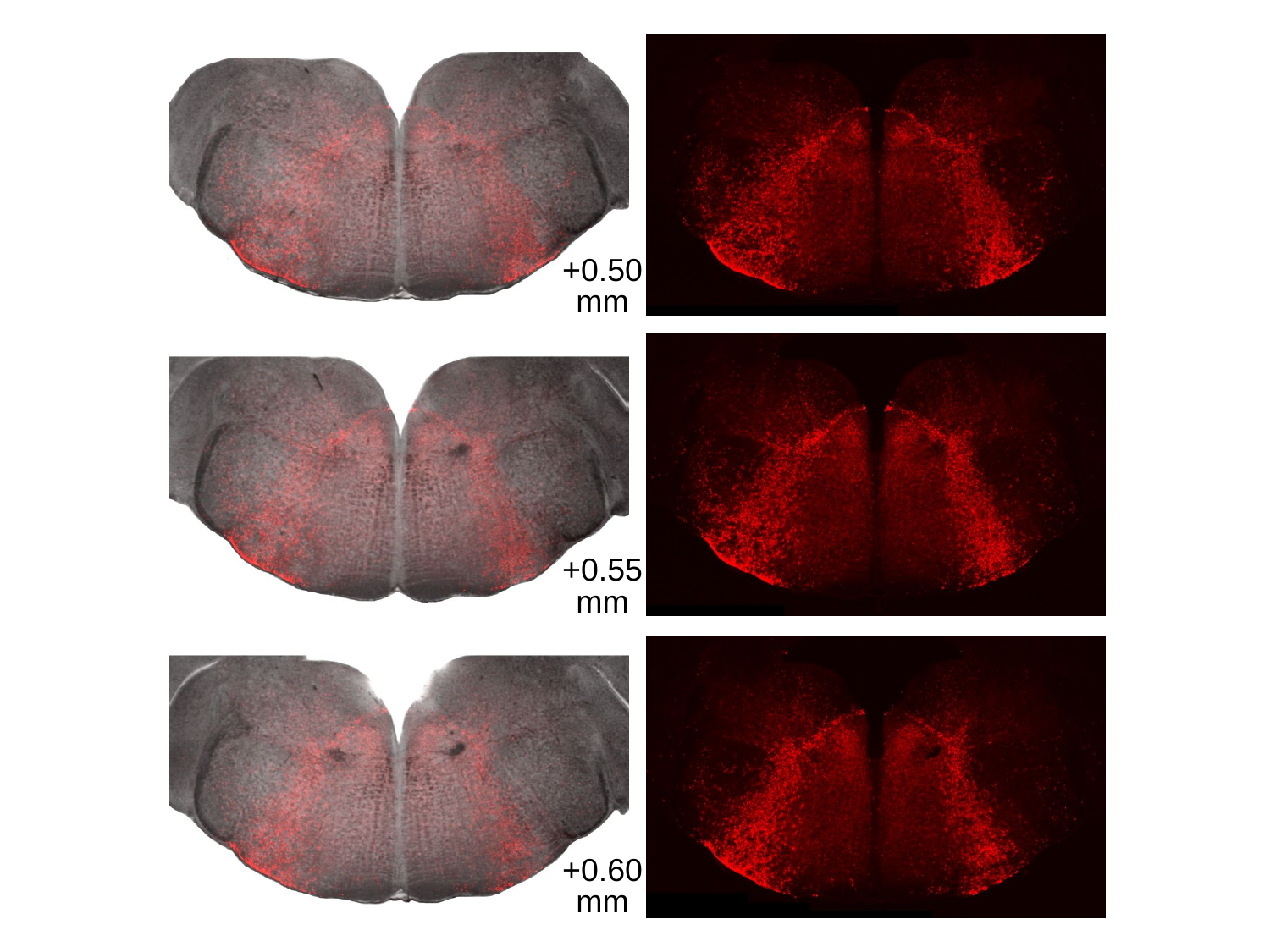

+0.50
mm
+0.55
mm
+0.60
mm
